# Longitudinal Repeatome Remodeling in Peripheral Blood Following Parkinson’s Disease Diagnosis

**DOI:** 10.3390/genes17050577

**Published:** 2026-05-18

**Authors:** Jerzy K. Kulski, Sulev Koks

**Affiliations:** 1 School of Biomedical Science, Discipline of Microbiology and Immunology, The University of Western Australia, Crawley, WA 6009, Australia; 2 Personalised Medicine Centre, Health Futures Institute, Murdoch University, Perth, WA 6150, Australia; 3 Perron Institute for Neurological and Translational Science, Perth, WA 6009, Australia

**Keywords:** peripheral blood repeat transcriptomics, Parkinson disease, transposable elements, small RNA pseudogenes, repeatome, longitudinal biomarkers, immune regulation

## Abstract

Background/Objectives: Parkinson’s disease (PD) is associated with systemic molecular alterations that extend beyond the central nervous system, including changes in peripheral blood transcriptomic profiles. While prior studies have focused predominantly on coding-gene expression, the longitudinal behavior of the peripheral blood repeatome following clinical diagnosis remains poorly characterized. Here, we investigated temporal remodeling of repetitive-element transcription over 36 months post-diagnosis by integrating repeat subfamily- and locus-specific analyses. Methods: Repeatome expression was quantified using SalmonTE and DESeq2 in peripheral blood RNA-seq data from 1560 PD and control individuals at diagnostic baseline (BL) and four follow-up visits (6, 12, 24, and 36 months). Differential expression was assessed at the subfamily level, with additional locus-specific validation in a representative subset. Results: A total of 259 repeat subfamilies were differentially expressed (padj < 0.05), of which 224 (86.5%) were already detected at baseline. Enrichment of differential expression was significantly higher at baseline than at later visits (odds ratio = 30.9, *p* < 2.2 × 10^−16^), with limited additional divergence over time. Longitudinal analyses revealed non-linear trajectories in selected repeat families, including Alu and SVA subfamilies. Locus-specific analysis identified 237 significantly regulated elements, demonstrating heterogeneous, site-specific transcriptional changes, including clusters of differentially expressed loci and instances within PD-relevant genomic regions (e.g., *SNCA* and *IKZF2*). Conclusions: Peripheral blood repeatome expression differs between PD and control groups, with the dominant signal established at clinical diagnosis and modest longitudinal modulation thereafter. Integration of locus-level analysis indicates that subfamily level patterns arise from discrete genomic events rather than uniform regulation. These findings support a model of systemic, immune-associated transcriptomic remodeling in circulating blood cells and position the peripheral repeatome as a dynamic framework for biomarker discovery and future mechanistic investigation.

## 1. Introduction

Parkinson’s disease (PD) is a progressive neurodegenerative disorder with a multifactorial etiology involving aging, genetic susceptibility, environmental exposures, and immune dysregulation [1,2,3]. Clinically, PD is characterized primarily by motor symptoms, including bradykinesia, rigidity, and tremor, together with diverse non-motor manifestations such as cognitive decline and sleep disturbances [4]. Although the precise molecular mechanisms underlying disease onset and progression remain incompletely understood, hallmark pathological features include degeneration of dopaminergic neurons in the substantia nigra pars compacta and widespread accumulation of α-synuclein and tau within Lewy body pathology in both the central and peripheral nervous systems [5,6,7]. More than 90 genetic loci have been associated with PD risk through genome-wide association studies, including *GBA*, *SNCA*, *MAPT*, *PRKN*, and *LRRK2* [8,9]. Increasing evidence also implicates epigenetic alterations, including DNA methylation and histone modifications, in PD-associated molecular dysregulation [10,11]. Transcriptomic studies across multiple biospecimens, including post-mortem brain tissue, cerebrospinal fluid (CSF), plasma, serum, and whole blood, have further expanded our understanding of disease-associated molecular pathways [10,11,12,13,14]. Integration of transcriptomic data with expression quantitative trait locus (eQTL) and transcriptome-wide association analyses has identified numerous genes associated with PD susceptibility and longitudinal disease-related molecular signatures [15,16].

Peripheral blood transcriptomes have emerged as a valuable source of molecular biomarkers and systemic biological insight in PD, reflecting immune dysregulation, metabolic alterations, and broader transcriptomic plasticity beyond the central nervous system [14,17,18,19,20,21,22]. Whole-blood RNA profiles may capture dynamic changes in circulating immune cells and stress-responsive transcriptional programs during the post-diagnostic phase of the disease. For example, Craig et al. [18] reported altered leukocyte and lymphocyte transcriptomic signatures in both prodromal individuals and clinically diagnosed PD patients. Type I interferon signaling has also emerged as an important component of neuroinflammatory and peripheral immune responses associated with PD [23,24]. In this context, the Parkinson’s Progression Markers Initiative (PPMI) provides a valuable public resource comprising long-term, multicohort, longitudinal clinical and molecular data for biomarker discovery and disease monitoring [25]. Established in 2010, PPMI includes clinically diagnosed PD patients, genetically at-risk individuals, prodromal participants, and healthy controls, together with extensive clinical, imaging, genomic, and transcriptomic datasets. Whole-blood RNA-seq data from more than 1560 participants are publicly available and provide an important framework for investigating longitudinal molecular remodeling following clinical diagnosis [18,26,27].

Beyond reanalyses of PD-associated gene expression, regulatory variation, and DNA methylation patterns [10,18,28], transcriptomic variation arising from genomic repetitive elements, collectively termed the repeatome [29], has attracted increasing attention in recent years [30,31]. The repeatome includes diverse classes of repetitive sequences, including transposable elements (TEs) such as Long Interspersed Nuclear Elements (LINEs) [32], Short Interspersed Nuclear Elements (SINEs; e.g., Alu and MIR), endogenous retroviruses (ERVs), SINE-VNTR-Alu (SVAs) [33,34], pseudogenes derived from small non-coding RNAs (e.g., rRNA, snRNA, and tRNA), long non-coding RNAs, and tandem repeat sequences such as simple and satellite repeats [26,35,36]. Once widely regarded as “junk DNA,” these elements are now recognized as important contributors to chromatin organization, transcriptional regulation, innate immune signaling, and transcriptomic plasticity in both health and disease [28,37]. Although only a small fraction of transposable elements is transcriptionally active under most physiological conditions, repeat expression may increase in response to developmental programs, cellular stress, infection, aging, and disease-associated chromatin remodeling [38,39]. Repeatome transcription is highly heterogeneous across tissues [40], including the brain, central nervous system, and peripheral blood in neurodegenerative and aging-related contexts [31,41,42,43].

Several repeat families are functionally linked to immune- and stress-responsive transcriptional pathways. LINE-1 (L1), endogenous retroviral (ERV), and other long terminal repeat (LTR) elements may modulate type I interferon responses and inflammatory signaling by acting as endogenous viral mimics or sources of double-stranded RNA (dsRNA), thereby engaging innate immune sensing pathways in both the brain and peripheral blood [44,45,46]. Alu elements are particularly abundant in leukocytes and may contribute to dsRNA-associated stress responses and inflammasome activation [47,48]. SVA elements, a primate-specific composite TE family, have also been implicated in transcriptional modulation within immune cells [49]. In addition, small RNA-derived pseudogenes such as 7SK and 7SL, which share structural and evolutionary relationships with Alu-like sequences, participate in transcriptional elongation and RNA trafficking, respectively, and their dysregulation has been reported under cellular stress and disease-associated conditions [50,51,52]. Tandem repeats, including satellite and simple repeat sequences, may further influence transcriptional activity through effects on chromatin compaction, nucleolar organization, and genome architecture [53].

Despite growing evidence linking repeatome transcription to immune signaling, cellular stress responses, and neurodegenerative disease-associated molecular states, the longitudinal dynamics of repeat element expression in peripheral blood following clinical PD diagnosis remain poorly defined. In this study, we applied SalmonTE and DESeq2 [54] to quantify repeatome expression across five clinical visits spanning 36 months in PD patients and healthy controls. We hypothesized that repeatome expression is differentially regulated in PD relative to controls, with a substantial component of dysregulation established at diagnostic baseline and evolving over time in a non-linear manner. Our aims were to characterize longitudinal repeatome remodeling, identify temporal transcriptional shifts following diagnosis, and assess whether peripheral repeatome expression reflects systemic transcriptomic adaptation and immune-associated regulatory plasticity during PD progression.

## 2. Materials and Methods

### 2.1. PPMI Study Cohort and Whole-Blood RNA-Seq Data

Whole-blood RNA-seq data were obtained from the Parkinson’s Progression Markers Initiative (PPMI) cohort at five clinical timepoints: baseline (BL) at diagnosis and follow-up visits at 6 months (V02), 12 months (V04), 24 months (V06), and 36 months (V08), as previously described [27,36]. Baseline (BL) was defined as the study entry visit corresponding to the time of clinical Parkinson’s disease (PD) diagnosis, with subsequent visits representing longitudinal follow-up over 36 months. The baseline diagnostic visit (time 0) was used as the anchor point (“diagnostic baseline”) for all longitudinal comparisons. The subsequent 3-year interval, spanning V02 to V08, represents the post-diagnostic phase of longitudinal follow-up, during which disease progression may vary between individuals in both degree and direction.

Clinical metadata were available for BL, V04, V06, and V08, whereas V02 represented an RNA-seq-only timepoint without corresponding clinical assessment. Clinical covariates available from the PPMI cohort included age, sex, visit ID, Hoehn–Yahr staging, UPDRS scores, medication use, and selected cognitive and molecular biomarkers (Table S1). The 36-month interval captures post-diagnostic longitudinal clinical progression.

The dataset included both healthy control (CO) participants and clinically confirmed PD cases. Detailed procedures for blood collection, RNA extraction, library preparation, and sequencing have been published elsewhere [18]. The PPMI study was approved by institutional review boards at contributing clinical sites and conducted in accordance with the Declaration of Helsinki [21]. All participants in the PPMI public dataset provided written informed consent.

### 2.2. Repeatome Quantification and SalmonTE Pipeline

Repeatome and TE expression were quantified using the SalmonTE pipeline (SalmonTE v0.4.0) [54]. Raw FASTQ files were preprocessed with Trimmomatic to remove adapters and low-quality bases. Processed reads were pseudoaligned against a curated repeat subfamily reference derived from Repbase [55] and RepeatMasker consensus sequences [56], with classification based on the Dfam annotation system [57]. SalmonTE pseudoaligned reads to 5947 repeat element subfamilies, including interspersed repeats, DNA transposons, LINEs, SINEs (e.g., Alu), endogenous retroviruses (ERVs), small RNA pseudogenes (e.g., 7SK, Y, 7SL, HY), SVAs, tandem repeats, and other repetitive elements. The pipeline generated count matrices for downstream analysis, including normalized expression values and baseMean estimates using DESeq2 (Table S2).

### 2.3. Differential Expression Analysis

Differential expression (DE) analyses were performed using DESeq2 (v1.36.0) on SalmonTE count matrices. Three analytical frameworks were applied:

(1) Between-group comparisons: PD vs. CO at each visit.
(2) Within-group longitudinal comparisons: PD vs. BL and CO vs. BL at each follow-up timepoint.
(3) Cross-timepoint (Venn) analyses: identification of shared and unique significantly expressed elements across visits.

Normalization across samples and timepoints was performed using DESeq2’s median-of-ratios method, which accounts for library size and compositional differences. Potential batch effects were minimized using a consistent processing pipeline across all samples within the PPMI dataset and, where applicable, incorporation of relevant covariates into the statistical model. Statistical significance was defined as an adjusted *p*-value (padj < 0.05) following Benjamini–Hochberg correction to control the false discovery rate in the context of large-scale repeatome profiling (Table S2).

### 2.4. Locus-Specific Differential Expression Analysis of Transposable Elements

To evaluate whether repeat expression patterns observed at the subfamily level reflect locus-specific transcriptional changes, we performed differential expression analysis at the level of individual transposable element (TE) loci using a subset of baseline (BL) whole-blood RNA-seq samples from female participants. Locus-resolved TE expression counts were obtained from preprocessed alignment-based quantification (as provided), with each TE locus treated as an independent feature defined by genomic coordinates and RepeatMasker annotation. Differential expression analysis was conducted using DESeq2, applying standard normalization and dispersion estimation procedures. Statistical significance was determined using the Wald test with multiple-testing correction via the Benjamini–Hochberg method, and loci with adjusted *p*-values (padj < 0.05) were considered significant. Annotated TE loci were categorized into major classes (e.g., Alu, LINE-1, ERV/LTR, and DNA transposon-derived elements) based on element naming conventions. Genomic positions (chromosome, start, end) were retained to enable regional and gene-context analyses. Gene symbols associated with locus-specific TE insertions were manually assigned to broad functional categories (e.g., immune/inflammatory, neuronal/synaptic, metabolic/mitochondrial, transcription/chromatin, RNA processing) based on curated gene function descriptions. For visualization, locus-level results were summarized using volcano plots and genomic position-based scatter plots, highlighting spatial clustering and mixed-direction expression patterns within defined chromosomal intervals.

This analysis was exploratory and intended to provide locus-level validation of subfamily level trends. Given the use of bulk RNA-seq data and restriction to a subset of samples, the results should be interpreted cautiously and do not establish causal or regulatory relationships between TE loci and nearby genes.

### 2.5. Time Series and Subfamily Level Analyses

To characterize temporal patterns of repeat expression, DE results were aggregated at the class, family, and subfamily levels. Line plots of baseMean and log_2_ fold-change values across timepoints were generated using the ggplot2 package. Heatmaps were produced using the pheatmap and ComplexHeatmap R packages. For each family and subfamily, summaries were tabulated according to statistical significance (padj < 0.05), direction of change (up- or down-regulated), and timepoint. Venn diagrams comparing significantly expressed repeat elements between groups were generated using jvenn (https://www.bioinformatics.com.cn/static/others/jvenn_en/example.html, accessed on 12 October 2025), based on elements reaching padj < 0.05 at any post-baseline visit.

### 2.6. Power Analysis

Statistical power for detecting differential expression was estimated using the pwr package in R. Power was calculated under a two-sample *t*-test framework with alpha = 0.05 and effect sizes (Cohen’s d) ranging from 0.2 to 0.5, using the actual sample sizes for each timepoint. These estimates were used to evaluate observed versus expected significance rates across TE classes and to contextualize the proportion of DE elements relative to detectable effect sizes.

### 2.7. Statistical Comparisons and Enrichment Tests

Differences in the numbers of significantly expressed repeats between the PD and CO groups were assessed using Fisher’s exact tests or Chi-squared tests, depending on cell counts in contingency tables. Multiple-testing correction was applied using the Benjamini–Hochberg FDR. Enrichment of repeat families or subfamilies was evaluated using contingency tables with FDR-adjusted *p*-values. Observed-vs-expected significance rates across repeat classes were assessed using power-adjusted proportions. All statistical analyses were performed in R v4.2.2.

### 2.8. Data Visualization and Figure Generation

Most analyses and figures were performed in R (v4.2.2) using the packages ggplot2, pheatmap, ComplexHeatmap, and VennDiagram, with charts generated in Excel (v16.102.3). Custom R scripts for figure generation are included in Supplementary Materials (Supplementary_Materials3_RScripts). Tables were constructed using baseR, the tidyverse, or Excel.

### 2.9. Data Availability

Raw and processed RNA-seq data used in this study were obtained from the PPMI dataset (https://www.ppmi-info.org/access-data-specimens/download-data, accessed 19 January 2021). SalmonTE count matrices, analysis code, and Supplementary Materials generated by Salmon TE and DE analyses are available upon reasonable request.

## 3. Results

### 3.1. Clinical PPMI Cohort Characteristics

Clinical metadata extracted from the public PPMI dataset confirmed that the longitudinal RNA-seq cohort comprised 1560 individuals at baseline, with progressive attrition at subsequent follow-up visits over 36 months (Table 1). The overall mean age of participants was approximately 61 years, with comparable ages between PD and control groups (PD: 61.4 years; controls: 60.7 years). The cohort was predominantly male (64.6%), with females representing 35.4% of participants. Clinical variables available for baseline and follow-up visits included Hoehn–Yahr stage, UPDRS severity scores, medication use, and selected cognitive and biomarker measures, whereas V02 represented an RNA-seq-only timepoint without matched clinical assessment (Table S1).

### 3.2. PPMI Cohort and Peripheral Blood Repeatome Overview

Peripheral blood repeatome expression was profiled in Parkinson’s disease (PD) patients and matched healthy controls (CO) at the diagnostic baseline (BL) and four follow-up visits over 36 months (6, 12, 24, and 36 months; V02–V08; Table 1). Sample sizes ranged from 193 to 835 individuals per group across timepoints, providing >97% statistical power to detect modest expression differences (log_2_ fold-change ≥0.2).

Using SalmonTE, a total of 5947 expressed repeat subfamilies were quantified across all samples. Of these, 259 subfamilies (4.4%) were significantly differentially expressed (padj < 0.05) between PD and CO at one or more timepoints (Table 2, Tables S2 and S3).

### 3.3. Diagnostic Baseline (BL) Dominates Intergroup Repeatome Differences

The majority of repeatome differences between PD and CO were present at the diagnostic BL. Specifically, 224 of 259 significant subfamilies (86.5%) were already differentially expressed at BL (Table 2, Table 3 and Table S3). Fisher’s exact test demonstrated strong enrichment of significant subfamilies at BL compared with all follow-up visits (*p* < 2.2 × 10^−16^; OR = 30.9), indicating that intergroup differences were most pronounced at the time of clinical diagnosis.

Across subsequent visits (V02–V08), only 35 repeated significant observations (11 unique subfamilies) remained differentially expressed beyond BL, indicating that most detectable intergroup differences were concentrated at BL, with more limited persistence or re-emergence over time.

**Figure 1:**
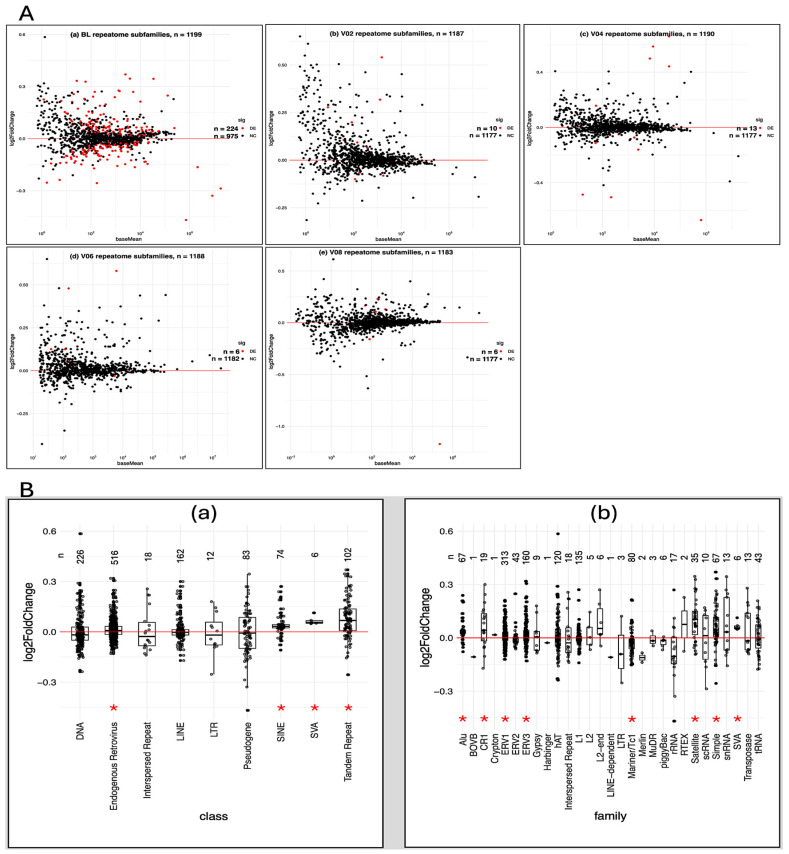
Peripheral blood repeatome expression in PD versus controls (CO). (**A**) MA plots of repeatome subfamily expression across five timepoints (BL, V02, V04, V06, and V08). Each panel (**a**–**e**) displays the log_2_ fold-change (PD vs. CO; *y*-axis) plotted against the mean normalized expression (baseMean; *x*-axis). Differentially expressed subfamilies (padj < 0.05) are shown in red, whereas non-significant TE subfamilies (padj ≥ 0.05) are shown in black. The number of significant and non-significant elements is indicated in each panel. Positive log_2_ fold-change values indicate higher expression in PD, whereas negative values indicate higher expression in COs. These plots summarize temporal patterns of repeatome dysregulation over the 36-month follow-up. (**B**) Differential expression of repeat classes and families at baseline (BL). (**a**) Log_2_ fold-change (log_2_FC) distributions for 9 repeat classes; (**b**) distributions for 29 repeat families. Each boxplot displays the median (horizontal line), interquartile range (box), and variability (whiskers) of expressed subfamily elements. Red asterisks indicate statistically significant differential expression (*p* < 0.05). The number of contributing subfamily elements (*n*) is shown above each box.

**Figure 2:**
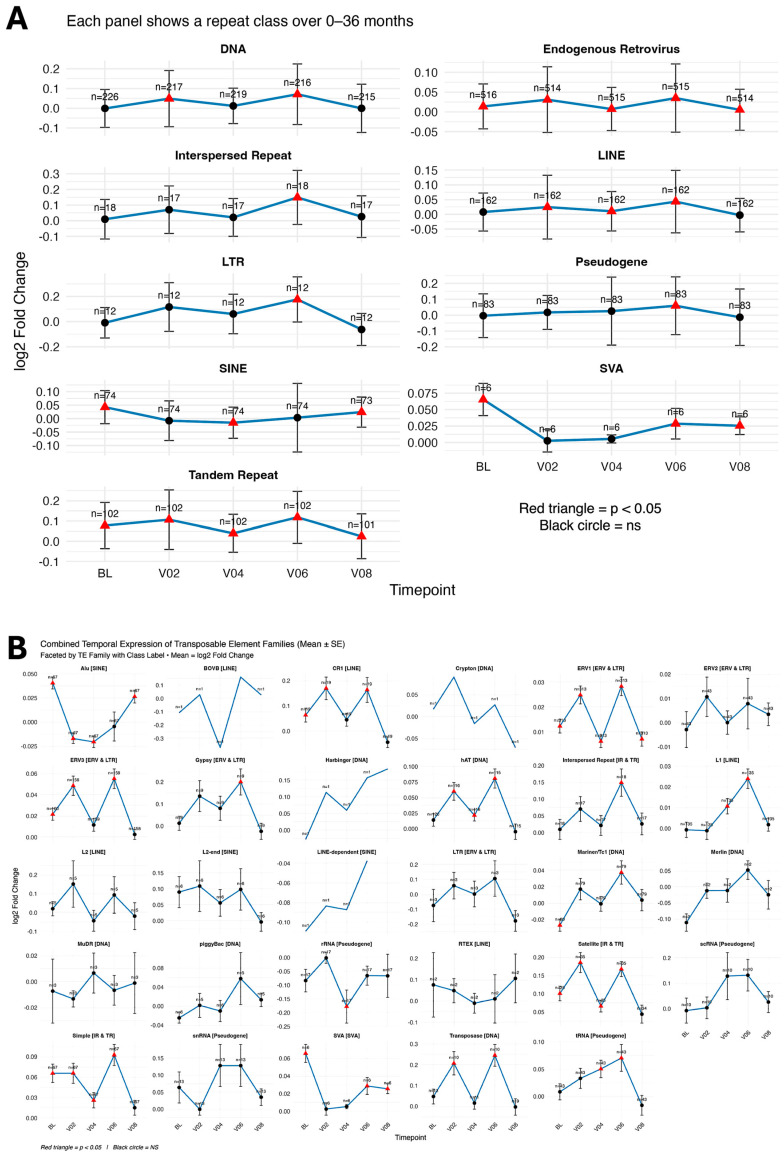
Temporal remodeling of repeat classes and family expression over 36 months (PD vs. CO). (**A**) Expression trajectories of nine major repeat classes (DNA transposons, ERV/LTRs, LINEs, SINEs, SVAs, pseudogenes, and other tandem or interspersed repeats). Points represent mean log_2_ fold-change at BL (0 months), V02 (6 months), V04 (12 months), V06 (24 months), and V08 (36 months). Vertical bars indicate standard deviation (SD). Red triangles mark significant differential expression (*p* < 0.05), and black circles indicate non-significant values. Sample sizes (*n*) are shown above each point. (**B**) Temporal expression profiles of 29 repeat families grouped by class (e.g., Alu [SINE], ERV1 [ERV/LTR], L1 [LINE]). Points represent mean log_2_ fold-change at each timepoint, with vertical bars indicating standard error (SE). Sample sizes (*n*) are shown above each point. Red triangles denote significant changes (*p* < 0.05), and black circles denote non-significant changes. Panels A and B use panel-specific y-axis scales to preserve resolution of temporal changes across repeat groups with differing dynamic ranges; therefore, comparisons should focus on temporal patterns rather than absolute magnitudes between panels. These trajectories highlight the heterogeneous, class-dependent behavior of repeat elements during disease progression.

At the repeat class level, significant BL differences were observed in ERVs, SINEs, SVAs, and tandem repeats. At the repeat family level (29 families), significant differences (*p* < 0.05) were identified in Alu, CR1, ERV1, ERV3, Mariner/TC, SVA, satellite repeats, and simple repeats (Table 3; Figure 1). Temporal visualization of repeat classes and families across the 36-month period is shown in Figure 2. Temporal patterns rather than absolute magnitudes are presented across panels to highlight the heterogeneous, class-dependent behavior of repeat elements during disease progression.

#### Subfamily Level Temporal Architecture of Repeatome Expression

Figure S1 summarizes mean normalized expression values, log_2_ fold-change trajectories, and heatmaps for significantly expressed repeat subfamilies across the 36-month longitudinal window. At diagnostic BL, these subfamilies exhibited heterogeneous expression patterns, including both upregulation and downregulation relative to controls (Table 3). Among 120 ERV-associated signals (including duplicated observations across timepoints), 61 subfamilies (including HERV18, HERVEa, LTR22E, and HERVK3I) were downregulated, whereas 59 subfamilies (including MLT1E1A, LTR9, MLT1J, and LTR24) were upregulated (Table S4), indicating a broadly balanced but subtype-specific pattern of ERV regulation.

**Figure 3:**
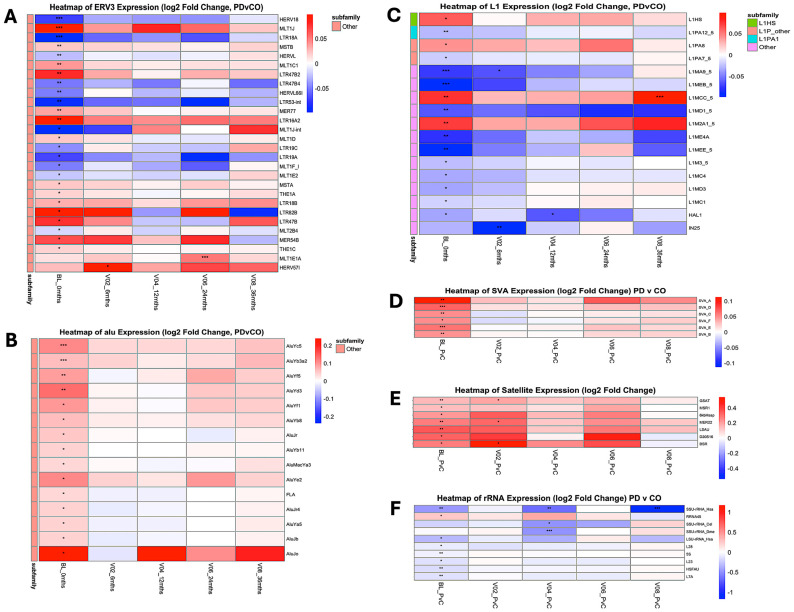
Heatmaps of differential expression for representative repeat subfamilies in PD vs. CO. (**A**–**F**) Heatmaps showing log_2_ fold-change values (PD vs. CO) at baseline (BL) and follow-up visits V02 (6 months), V04 (12 months), V06 (24 months), and V08 (36 months). Panels represent selected repeat subfamilies: (**A**) 28 ERV1 elements; (**B**) 15 Alu elements; (**C**) 17 L1 elements; (**D**) 6 SVA elements; (**E**) 7 satellite tandem repeats; and (**F**) 10 rRNA elements. Asterisks denote statistical significance based on adjusted *p*-values: padj < 0.05 (*), padj < 0.01 (**), and padj < 0.001 (***).

In contrast, Alu, SVA, and satellite tandem repeat subfamilies were consistently upregulated at the diagnostic BL (Table 3). All 15 significant Alu subfamilies and all six significant SVA subfamilies exhibited non-linear temporal trajectories, characterized by reduced expression at V02 followed by progressive re-elevation toward V08. These patterns are illustrated in Figure 3, which presents representative heatmaps illustrating longitudinal expression patterns for ERV3, LINE-1, Alu, SVA, satellite tandem repeats, and rRNA-associated subfamilies across all timepoints.

### 3.4. Longitudinal Repeatome Remodeling Across Diagnostic and Post-Diagnostic Phases

**Figure 4:**
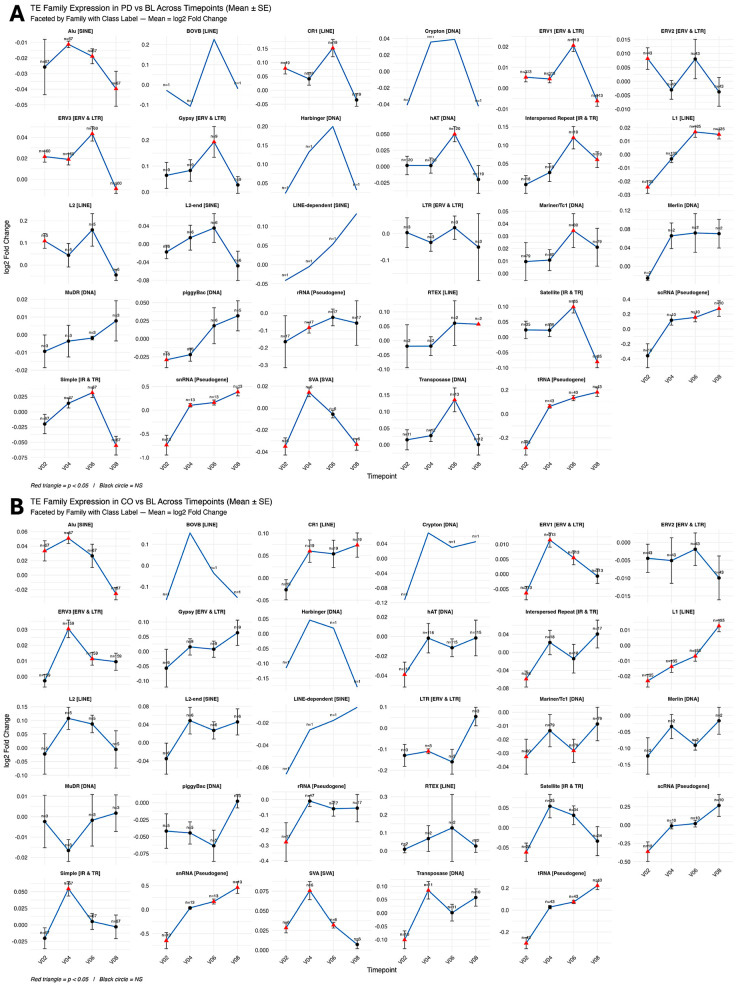
Longitudinal expression of 29 transposable element (TE) families relative to baseline (BL). (**A**) PD vs. BL. (**B**) controls (CO) vs. BL. Each panel shows the expression trajectory of a TE family, labeled by its corresponding class (e.g., Alu [SINE], ERV1 [ERV/LTR], L1 [LINE]). Values represent mean log_2_ fold-change relative to BL at four follow-up timepoints: V02 (6 months), V04 (12 months), V06 (24 months), and V08 (36 months). Vertical bars indicate standard error (SE), and sample sizes (*n*) are shown above each point. Red triangles indicate significant differential expression (*p* < 0.05), and black circles indicate non-significant changes. These trajectories illustrate class-dependent and family-specific patterns of TE regulation over time.

Longitudinal within-group analyses (PD and CO relative to their respective BLs) revealed widespread temporal variation in repeatome expression across the 36-month follow-up period. Across all timepoints, 304 repeat subfamilies (6.3%) in PD and 312 subfamilies (6.6%) in CO exhibited significant temporal differential expression (Table 2 and Table S3). Overall, both cohorts displayed non-linear temporal variation, with increased numbers of differentially expressed subfamilies at V02, reduced differences at mid-timepoints (V04–V06), and a subsequent increase at V08 (Figure 4).

At the repeat class level, ERVs, SINEs, SVAs, and small RNA-derived pseudogenes showed recurrent temporal modulation across multiple timepoints in both PD and CO, whereas other repeat classes exhibited more restricted or transient variation (Figure 4 and Figure S2).

#### 3.4.1. Alu Repeat Remodeling

**Figure 5:**
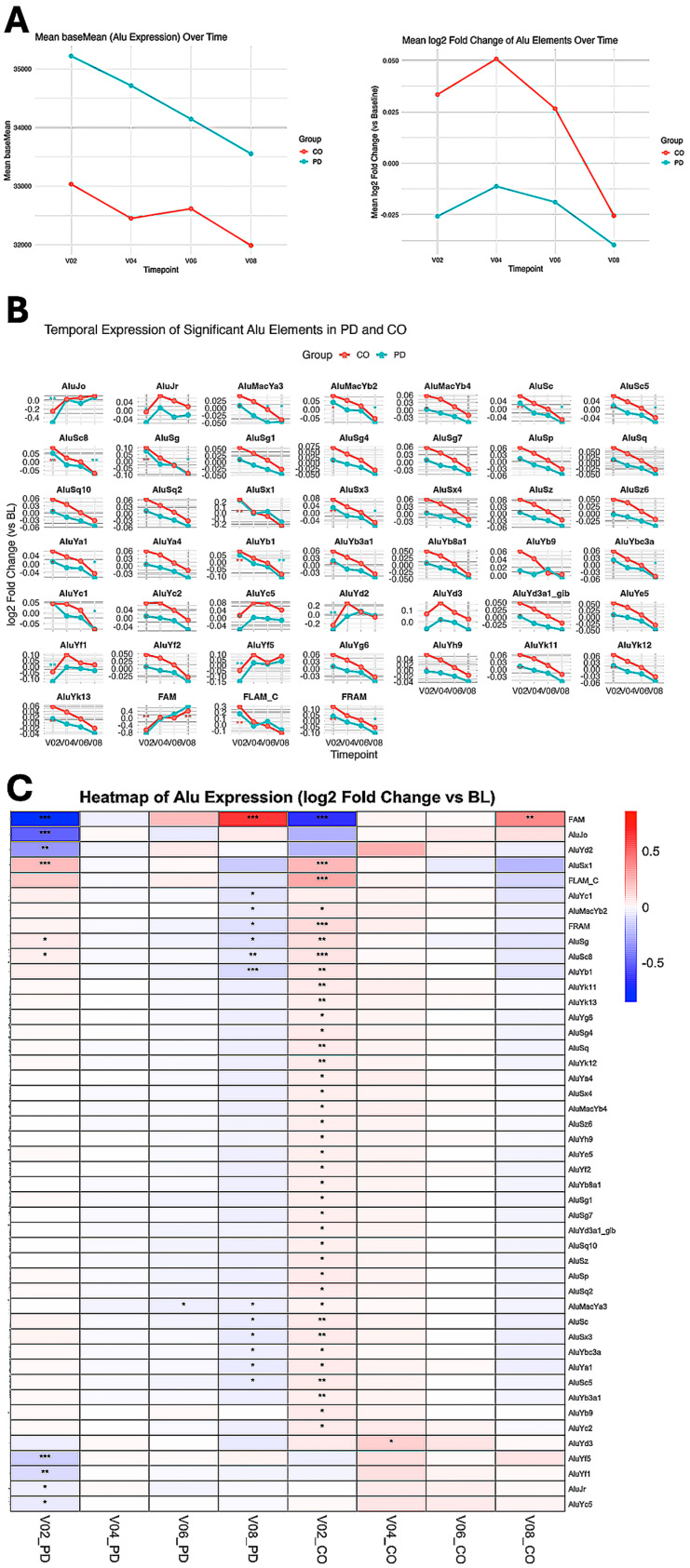
Temporal remodeling of Alu expression in peripheral blood from PD and controls (CO) (**A**–**C**). (**A**) Mean basemean expression (left) and mean log_2_ fold-change (right) for all expressed Alu elements at four follow-up timepoints (V02, V04, V06, V08) relative to baseline (BL) in PD (red) and CO (blue). Significant changes (adjusted *p* < 0.05) were observed at V04, V06, and V08 in PD and at V02, V04, and V08 in CO. (**B**) Line plots showing log_2_ fold-change trajectories for 46 Alu subfamilies across four timepoints relative to BL in PD and CO. Each facet corresponds to a distinct Alu subtype. Only subfamilies with at least one significant change (adjusted *p* < 0.05 at ≥1 timepoint) are shown. Points are colored by group (PD or CO); statistical significance is indicated by asterisks (padj < 0.05: *, <0.01: **). The trajectories reveal heterogeneous Alu regulation, including progressively downregulated elements (e.g., AluYb1) and group-specific temporal patterns. (**C**) Heatmap of log_2_ fold-changes for the same 46 Alu subfamilies in PD vs. BL and CO vs. BL comparisons across four timepoints. Asterisks denote statistical significance (padj < 0.05: *, <0.01: **, <0.001: ***).

A total of 46 Alu subfamilies showed significant temporal variation across the study period. Of these, 20 were significant in PD and 39 in CO, with 13 shared between groups. Temporal changes were observed predominantly at V02 and V08, with heterogeneous trajectories across subfamilies (Figure 5).

#### 3.4.2. Small RNA-Derived Pseudogene Remodeling

**Figure 6:**
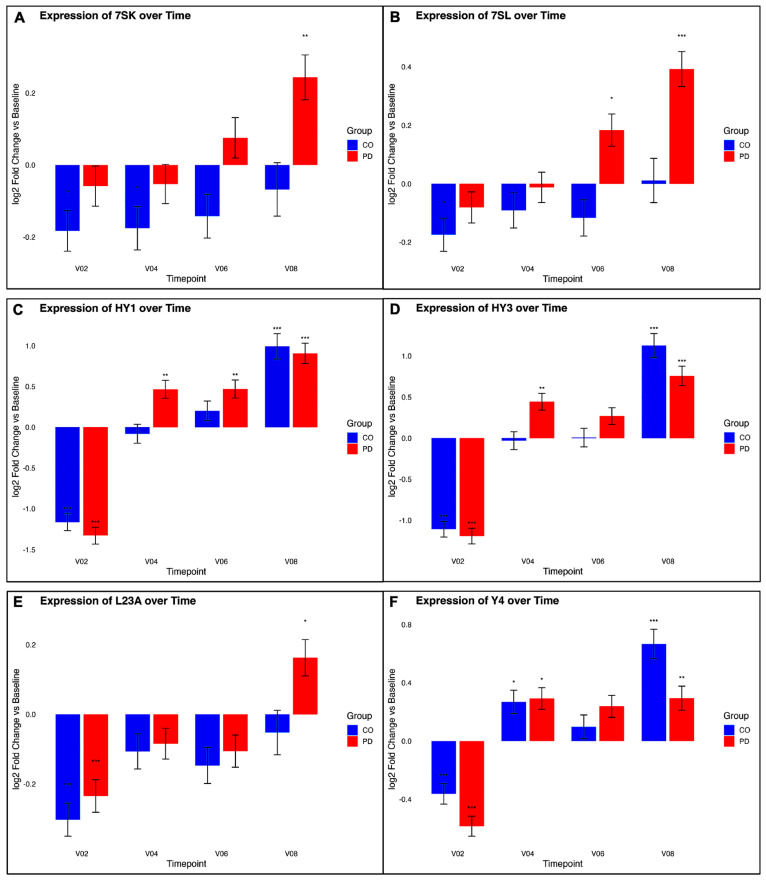
Longitudinal expression of selected small RNA-derived pseudogene elements in peripheral blood from PD and CO. (**A**–**F**) Each panel presents the temporal expression trajectory of a pseudogene family: (**A**) 7SK, (**B**) 7SL, (**C**) HY1, (**D**) HY3, (**E**) L23A and (**F**) Y4. Bar plots show log_2_ fold-change in expression relative to baseline (BL) at each timepoint (V02 = 6 months, V04 = 12 months, V06 = 24 months, V08 = 36 months). Error bars indicate the standard error of the log_2_ fold-change estimates (lfcSE). Colors distinguish controls (CO; blue) from PD (red). Asterisks denote statistical significance of differential expression compared to baseline within each group (* padj < 0.05, ** padj < 0.01, *** padj < 0.001).

Small RNA-derived pseudogene families exhibited time-dependent variation in both cohorts. In PD, six pseudogene families (7SK, 7SL, HY1, HY3, L23A, and Y4) showed increased expression at V08 relative to BL, whereas CO showed more stable or reduced temporal variation across the same period (Figure 6). Ribosomal RNA-derived pseudogenes showed reduced expression at later timepoints in PD.

#### 3.4.3. Other Repeat Families

Additional repeat families, including LINE-1 (L1), endogenous retroviruses (ERV1–3), DNA transposons, and SVAs, showed heterogeneous temporal expression patterns across both PD and CO groups. Most families exhibited increased differential expression at V02, reduced changes at V04–V06, and renewed variation at V08 (Figures S3 and S4).

### 3.5. Shared and Unique Repeat Signatures

**Figure 7:**
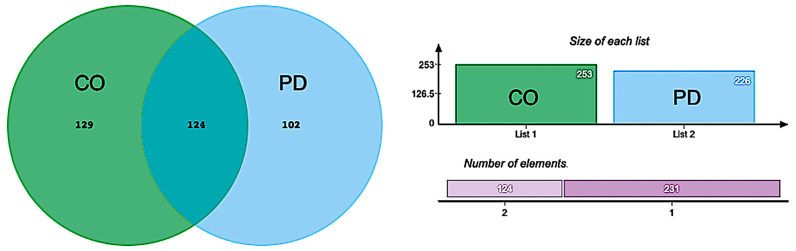
Overlap of significantly expressed repeat elements in PD and CO groups across four post-baseline timepoints. Venn diagram summarizing the overlap of significantly differentially expressed repeat elements (adjusted *p* < 0.05) identified in Parkinson’s disease (PD) and control (CO) groups at four post-baseline visits (V02, V04, V06, V08). Elements include transposable elements, small RNA pseudogenes, and simple or interspersed repeats. Each circle represents the set of significant elements unique to either PD or CO, with the intersecting region indicating elements shared between groups. Counts of total significant elements and shared elements are reported on the right-side of the Venn diagram. Repeat lists were derived from Table S5 (PD) and Table S6 (CO) and analyzed using the jvenn tool.

Venn analysis identified 355 significantly regulated repeat subfamilies across all timepoints, classified as PD-only (102), CO-only (129), or shared between groups (124) (Figure 7; Tables S5–S7).

Within the PD-only group, temporal expression patterns varied across subfamilies, with a proportion showing higher levels of significance at V02, reduced differences at mid-timepoints (V04–V06), and increased differences at V08. Of the 102 PD-only elements, 31 (30%) overlapped with PD vs. CO differentially expressed subfamilies, and 19 of these were upregulated (Table 4). In addition, 27 of the 31 overlapping subfamilies (87%) were already differentially expressed at V02.

In contrast, CO-only repeats showed time-dependent variation characterized predominantly by gradual changes across the 36-month period, whereas shared repeats exhibited heterogeneous longitudinal trajectories in both groups (Figure 7).

### 3.6. Locus-Specific Validation of Representative Repeat Expression

Locus-specific analysis was performed on a baseline female subset due to the availability of high-quality, consistently processed data for this group. This restriction was applied to minimize technical variability and ensure a controlled comparison at locus resolution, rather than to infer sex-specific biological effects.

This analysis identified 237 significantly differentially expressed loci, including 154 upregulated and 83 downregulated elements (Supplementary Table S8). Gene symbols associated with locus-specific TE insertions were also manually assigned to broad functional categories (e.g., immune/inflammatory, neuronal/synaptic, metabolic/mitochondrial, transcription/chromatin, RNA processing) based on curated gene function descriptions.

Alu elements comprised the largest fraction of significant loci (106/165 classified elements), with a strong bias toward upregulation (79 upregulated vs. 27 downregulated). These findings indicate that Alu expression is not uniformly regulated across subfamilies but instead reflects localized, locus-specific transcriptional remodeling within and outside gene regions. Notably, multiple significant Alu loci were clustered within a restricted region on chromosome 14 (~49.5–50.3 Mb), where adjacent elements displayed both upregulation and downregulation.

**Figure 8:**
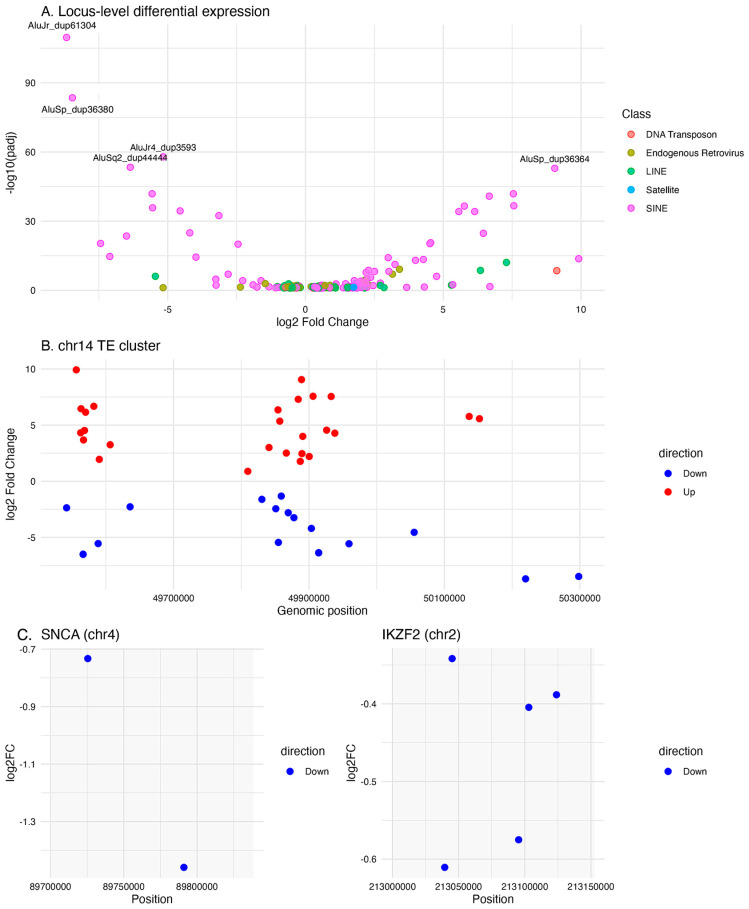
Locus-specific repeat expression reveals spatial clustering and gene-associated transcriptional context (**A**–**C**). (**A**) Volcano plot showing differential expression of transposable element (TE) loci at the diagnostic baseline in a female subset. Each point represents an individual TE locus, plotted as log_2_ fold-change versus −log_10_ adjusted *p*-value (padj), and colored by repeat class. Alu elements constitute the majority of significantly altered loci and show a predominance of upregulation, whereas LINE-1 and ERV/LTR elements display more heterogeneous patterns. Selected loci with high statistical significance are indicated. (**B**) Regional distribution of differentially expressed TE loci on chromosome 14 (~49.5–50.3 Mb), illustrating the coexistence of upregulated and downregulated elements within a confined genomic interval, consistent with localized transcriptional remodeling. (**C**) Genomic context of selected TE loci located within or proximal to Parkinson’s disease-associated gene regions, including *SNCA* (chr4) and *IKZF2* (chr2). TE loci are shown relative to gene boundaries, demonstrating that a subset of repeat elements is transcribed within gene-associated genomic regions. These associations are descriptive and do not imply functional regulatory relationships.

In contrast, L1 elements exhibited a more heterogeneous pattern (24 upregulated, 12 downregulated), while L2 elements showed a slight predominance of downregulation. In addition, several differentially expressed TE loci were located within or proximal to genes previously implicated in Parkinson’s disease biology. For example, two loci (MER103C_dup2389 and THE1D_dup3212) mapped within the genomic interval of *SNCA*, while seven additional loci (FLAM_C_dup3549, LTR33_dup1287, L3b_dup1750, ERVL-E-int_dup1426, MER5B_dup3667, MIRc_dup17289, and L3_dup7872) were located within the *IKZF2* gene region. These locus-resolved patterns, including spatial clustering and gene-associated transcriptional context, are illustrated in Figure 8 and suggest that a subset of repeat elements may be transcribed in genomic contexts relevant to PD-associated loci. However, given the locus-level resolution and the use of bulk RNA-seq data, these findings should be interpreted cautiously and do not establish functional regulatory relationships.

Overall, these results demonstrate that repeat expression patterns observed at the subfamily level arise from discrete, locus-resolved transcriptional events rather than uniform regulation across entire TE families.

### 3.7. Summary of Repeatome Patterns

Across all analyses, repeatome expression differences were predominantly present at the diagnostic baseline and showed variable temporal modulation over the 36-month follow-up period.

Observed patterns indicated consistent detection of Alu- and LINE-associated subfamilies across analytical comparisons, whereas ERV and SVA families showed more variable detection across timepoints and groups (Table 2 and Tables S3–S7). Alu elements and small RNA-derived pseudogenes exhibited more consistent longitudinal variation relative to other repeat classes, while ERV, LINE, SINE, and SVA families showed heterogeneous temporal profiles across both PD and CO cohorts.

Integration of Venn and longitudinal analyses indicated overlapping sets of repeat elements with early differential expression at BL and variable persistence across follow-up visits. Overall, these results describe time-dependent and group-specific variation in peripheral blood repeatome expression in PD and controls over a 36-month period following clinical diagnosis.

## 4. Discussion

This study provides a longitudinal analysis of peripheral blood repeatome expression in PD over a 36-month post-diagnostic interval. Whereas most previous studies of TE and repeat expression in neurodegenerative disease have been cross-sectional and limited to a single timepoint [35,40,43,58,59,60], the present analysis captures repeatome remodeling across five clinical visits from the diagnostic baseline to 36 months. This longitudinal design revealed that the majority of significant repeatome differences between PD and controls were already present at the diagnostic BL, with 224 of 259 significant subfamilies (86.5%) detected at study entry. In contrast, only a small proportion of repeat subfamilies showed persistent or re-emergent differences during follow-up, indicating that the dominant molecular signal was established at diagnosis and remained relatively stable over time. Importantly, statistical power remained high throughout the study despite reduced sample sizes at later visits, supporting the robustness of the observed temporal patterns.

A prominent finding was the coordinated longitudinal behavior of Alu subfamilies, which showed consistent upregulation at the diagnostic BL in PD relative to controls. Specifically, 15 of 67 Alu subtypes (22.4%) were significantly elevated at BL, followed by reduced expression at 6 months (V02) and progressive re-elevation by 36 months (V08), generating a clear non-linear U-shaped trajectory. This pattern may reflect elevated Alu-family transcription at the diagnostic BL followed by transient suppression and subsequent reactivation during the post-diagnostic period. However, rather than implying direct disease-specific TE activation, these longitudinal changes may more conservatively represent adaptive remodeling of repetitive-element transcription within heterogeneous circulating blood cell populations, potentially reflecting systemic immune and transcriptomic plasticity during disease progression. The contrasting temporal profiles observed in healthy controls further suggest that repeatome regulation in peripheral blood may capture broader differences in immune-state remodeling between PD and non-PD individuals. Similar differential Alu regulation has been reported in other neurodegenerative conditions, including Creutzfeldt–Jakob disease, supporting the broader relevance of repeatome remodeling in neurological disease states [61].

A second notable longitudinal pattern was the late upregulation at V08 of several small RNA-derived pseudogene families, including 7SL, 7SK, HY1, HY3, and Y4. Similar observations have been infrequently reported in repeatome studies [35,50,52], although recent analyses of PPMI samples by Kern et al. [26] identified significant temporal variation across multiple small RNA classes. Together with the Alu findings, these results suggest that small RNA pseudogene- and SINE-associated transcription may reflect systemic molecular adaptation within peripheral blood during the post-diagnostic phase of PD. At present, it remains unclear whether these changes represent direct contributors to disease-associated biology or secondary responses to immune and transcriptomic remodeling. Future analyses incorporating locus-level quantification and more frequent longitudinal sampling will be important for resolving these possibilities.

SVA retrotransposons showed similarly dynamic temporal behavior. Six SVA subtypes (A–F) were significantly elevated at the diagnostic BL in PD relative to controls, consistent with previous studies linking SVA activity to immune and neurodegenerative molecular states [33,34]. As observed for Alu subfamilies, SVA expression followed a non-linear trajectory over the 36-month period, with reduced expression at V02 followed by re-elevation at later visits. These findings are consistent with previous work from our group demonstrating that SVAs can act as cis-regulatory hubs within immune-associated genomic regions, including the major histocompatibility complex (MHC), where they influence local TE and gene transcriptional networks [59,62,63,64]. Although the present data were derived from peripheral blood and do not directly reflect central nervous system activity, the observed longitudinal SVA trajectories support the broader concept that repeat elements may participate in dynamic, immune-associated transcriptional regulation during the post-diagnostic course of PD.

Additional repeat classes also showed distinct temporal profiles. Most L1 elements and DNA transposons were reduced at the diagnostic BL in PD, with only modest and generally non-significant variation over the subsequent 36 months in both PD and control groups. A notable exception was the ancient LINE-1 subfamily *L1MCC_5*, which showed elevated expression at BL, progressive decline through intermediate visits, and re-elevation at V08. This distinct non-linear trajectory contrasts with the more stable profiles observed for most other L1-associated subfamilies and may indicate subtype-specific sensitivity to longitudinal changes in immune state, chromatin accessibility, or systemic transcriptional remodeling [32,44,65]. In contrast, ERV subfamilies represented a substantial proportion of the overlapping PD-associated repeat signatures, consistent with growing evidence that ERV-associated transcription may contribute to immune-responsive molecular states in neurodegenerative disease [66,67]. Taken together, these findings support a model in which different repeat families exhibit family- and subtype-specific temporal remodeling rather than uniform directional regulation across the repeatome.

Locus-specific analysis provided additional resolution to the repeatome patterns observed at the subfamily level, demonstrating that differential expression arises from discrete genomic loci rather than uniform regulation across entire TE families. In a baseline female subset, Alu elements comprised most significantly to the altered loci and exhibited a strong bias toward upregulation, consistent with the subfamily level enrichment observed at diagnosis. However, these changes were not uniformly distributed, with both upregulated and downregulated loci co-occurring within restricted genomic intervals, including clusters on chromosome 14. This spatial heterogeneity supports a model of localized transcriptional remodeling rather than global activation of repeat families. Future analyses incorporating both sexes will be required to evaluate sex-dependent repeatome regulation at the locus-specific level.

Importantly, a subset of differentially expressed loci mapped within or proximal to genes relevant to Parkinson’s disease biology, including *SNCA* and *IKZF2* [5,7,8,9]. Broad functional annotation of genes proximal to differentially expressed TE loci indicated enrichment across immune, neuronal, and transcriptional regulatory categories, supporting the hypothesis that locus-specific repeat expression occurs within biologically relevant genomic contexts. While these observations do not establish functional regulatory relationships, they suggest that repeat transcription may occur within genomic contexts linked to immune or neurodegenerative pathways. Given the use of bulk RNA-seq and the restriction to a subset of samples, these findings should be interpreted cautiously. Nevertheless, the concordance between subfamily level trends and locus-resolved signals supports the view that peripheral blood repeatome expression reflects a structured and context-dependent layer of transcriptional plasticity rather than stochastic transcriptional noise. These findings are also consistent with accumulating evidence that transposable element activation contributes to neurodegenerative processes through immune activation, genomic instability, and epigenetic dysregulation [13,31,41,58,60,66,67,68].

Mechanistically, differential repeat expression in peripheral blood may reflect activation of innate immune pathways, including double-stranded RNA sensing and type I interferon signaling [23,24,44,45,46,48]. Elements such as Alu- and ERV-derived transcripts can act as endogenous immune stimulators [64,66,67], potentially contributing to systemic inflammatory states observed in PD. While causal relationships cannot be established in this study, these pathways provide a plausible framework linking repeatome dysregulation to immune-associated disease processes.

The present findings support the concept that peripheral blood repeat transcription is not random background noise but instead reflects a heterogeneous layer of context-dependent regulatory potential within circulating blood cells [10,14,18,35,43,68]. Elements such as SVAs, Alus, MIRs, ERVs, and selected LINE-derived transcripts may contribute to local transcriptional regulation, innate immune signaling, or chromatin-state responsiveness, whereas many other expressed repeats may remain functionally neutral [37,39,43,69]. At the same time, several limitations should be acknowledged. First, the use of bulk peripheral blood RNA-seq does not allow direct resolution of cell-type-specific repeat expression and may reflect shifts in leukocyte composition over time. Second, reduced participant numbers at later visits, particularly V08, may limit statistical resolution for late longitudinal effects despite high overall analytical power. Third, matched epigenetic datasets, including DNA methylation and chromatin accessibility profiles, were not available for direct integration. Future studies incorporating locus-level repeat quantification, cell-type deconvolution, and multimodal epigenomic datasets will be essential to distinguish biologically meaningful repeat activity from background transcription and to clarify how peripheral repeatome remodeling relates to systemic immune adaptation during the post-diagnostic course of PD. The availability of age, sex, clinical severity, and medication-related covariates from the PPMI cohort strengthens interpretation of the longitudinal RNA-seq findings, although future models should also incorporate these variables directly into repeatome-specific regression analyses.

An additional important consideration is that analyses performed at the repeat class or family level may mask substantial locus-specific transcriptional heterogeneity, which is increasingly recognized as central to TE biology [70]. This is particularly relevant in regions such as the MHC, where our previous analyses in the PPMI cohort identified localized clusters of differential TE transcription [62,63], and similar locus-specific patterns may occur elsewhere in the genome. Future integration with single-cell RNA-seq approaches and computational cell-type deconvolution will be essential for resolving cell-type-specific repeatome signatures associated with immune-state variation and age-related hematological shifts [18,43,67,71,72]. Ultimately, higher-resolution locus-level mapping combined with targeted functional assays will be required to determine whether these repeatome changes contribute directly to PD-associated biology or primarily reflect downstream cellular state transitions.

## 5. Conclusions

This longitudinal analysis of peripheral blood repeatome expression demonstrates that repetitive-element transcription differs between Parkinson’s disease (PD) and healthy controls across the post-diagnostic 36-month interval. A substantial proportion of repeatome differences were already present at the diagnostic baseline and remained detectable over time, with Alu, ERV, LINE, SVA, and small RNA-derived pseudogene subfamilies showing the most prominent group-specific patterns.

The distinct temporal trajectories and subtype-specific profiles observed between PD and control groups support the concept that peripheral blood repeatome expression captures systemic transcriptomic and immune-associated remodeling during the post-diagnostic phase of PD. Rather than implying direct pathogenic mechanisms, these findings more conservatively indicate that repeat elements may serve as sensitive markers of changing cellular and regulatory states in circulating blood cells. More detailed analyses of repeat element expression at the level of loci and cell types are required as essential follow-up studies.

## Figures and Tables

**Table 1:** Cohort structure and selected clinical characteristics of the PPMI longitudinal RNA-seq cohort.

Visit	Group	*N* (RNA-Seq)	Mean Age (Years)	Male (%)	Female (%)	Clinical Assessment
BL	CO	725	60.7	64.6	35.4	Yes
BL	PD	835	61.4	64.6	35.4	Yes
V02	CO	388	–	–	–	RNA-seq only
V02	PD	475	–	–	–	RNA-seq only
V04	CO	356	61.3	–	–	Yes
V04	PD	529	61.3	–	–	Yes
V06	CO	319	61.2	–	–	Yes
V06	PD	531	61.2	–	–	Yes
V08	CO	193	61.3	–	–	Yes
V08	PD	366	61.3	–	–	Yes

V02 represents an RNA-seq-only visit without matched clinical assessment in the original PPMI clinical dataset.

**Table 2:** Number and percentage of significantly differentially expressed repeat subfamilies across longitudinal timepoints and cohort comparisons (padj < 0.05).

Cohort	Timepoint	TE Expressed (*n*)	Expressed (%)	TE Total (*n*)	Total (%)
PD vs. CO	BL	224	86.5	1199	18.7
PD vs. CO	V02	10	3.9	1187	0.8
PD vs. CO	V04	13	5.0	1190	1.1
PD vs. CO	V06	6	2.3	1188	0.5
PD vs. CO	V08	6	2.3	1183	0.5
PD vs. CO (total)		259	100.0	5947	4.4
PD vs. BL	V02	179	58.8	1196	15
PD vs. BL	V04	6	2.0	1200	0.5
PD vs. BL	V06	26	8.6	1201	2.2
PD vs. BL	V08	93	30.6	1197	7.8
PD vs. BL (total)		304	100.0	4794	6.3
CO vs. BL	V02	179	57.4	1192	15.1
CO vs. BL	V04	84	26.9	1191	7.1
CO vs. BL	V06	2	0.6	1189	0.2
CO vs. BL	V08	47	15.1	1186	4
CO vs. BL (total)		312	100.0	4758	6.6

**Table 3:** A total of 235 (padj < 0.05) significantly expressed transposable elements (TEs) and repeat elements categorized into 21 family groups and 9 class groups over 3 years of follow-up in PD versus control groups.

Family	Class	Up-	Down-	Total (*n*)	BL (*n*)	Other	All TEs	% of All
		Regulated (*n*)	Regulated (*n*)			Visits (*n*)	at BL (*n*)	TEs at BL
Alu	SINE	15	0	15	15	0	67	22.4
CR1	LINE	1	1	2	2	0	19	10.5
ERV1	ERV	38	36	74	73	7	313	23.3
ERV2	ERV	2	9	11	11	1	43	25.6
ERV3	ERV	16	12	28	26	2	180	14.4
hAT	DNA	3	7	10	9	4	120	7.5
IR	IR	1	3	4	4	0	18	22.2
L1	LINE	4	13	17	16	4	135	11.9
L2	LINE	0	1	1	1	1	5	20
L2-end	SINE	1	0	1	1	0	6	16.7
LTR	LTR	0	2	2	2	0	3	66.7
Mariner/Tc1	DNA	2	7	9	8	1	80	10
Merlin	DNA	0	1	1	1	0	2	50
MuDR	DNA	1	0	1	1	0	3	33.3
rRNA	Pseudo	1	9	10	8	4	17	47.1
Satellite	TR	7	0	7	7	3	35	20
scRNA	Pseudo	2	3	5	3	2	10	30
Simple	TR	16	4	20	20	2	67	29.9
snRNA	Pseudo	3	2	5	5	3	13	38.5
SVA	SVA	6	0	6	6	0	6	100
tRNA	Pseudo	3	3	6	5	1	43	11.6
Total		122	113	235	224	35	1185	18.9

Pseudo, pseudogene-derived repeats; IR, interspersed repeats; TR, tandem repeats. “Total (*n*)” represents the number of unique repeat subfamilies significantly differentially expressed (padj < 0.05), whereas the value including duplicates (*n* = 259) reflects repeated detection across multiple timepoints. “Other Visits” includes repeat subfamilies identified at follow-up timepoints (V02–V08) and may include duplicates due to overlap across visits. “All TEs (*n*)” denotes the total number of annotated repeat subfamilies at baseline, as reported in Table S2.

**Table 4:** Enrichment of significantly expressed repeat subfamilies in PD versus CO using Fisher’s exact test.

TE_Subfamily	Significance in PD (*n*)	Total_TEs (*n*)	Significance in CO (*n*)	Fisher’s Exact *p*-Value	FDR_Adjusted *p*-Value
**Alu**	20	67	39	**0.0016**	**0.03**
CR1	2	19	2	1.0000	1.00
ERV1	47	313	39	0.4165	1.00
ERV2	5	43	8	0.5486	1.00
ERV3	17	160	26	0.1893	0.90
LTR	1	9	1	1.0000	1.00
hAT	13	120	10	0.6618	1.00
interspersed	0	18	1	1.0000	1.00
L1	46	135	43	0.7958	1.00
L2-end	1	6	0	1.0000	1.00
Mariner/Tc1	9	80	12	0.6405	1.00
piggyBac	1	6	1	1.0000	1.00
rRNA	8	17	15	**0.0255**	0.24
Satellite	4	35	5	1.0000	1.00
scRNA	6	10	7	1.0000	1.00
Simple	12	67	5	0.1175	0.74
snRNA	9	13	10	1.0000	1.00
SVA	1	6	4	0.2424	0.92
tRNA	24	43	25	1.0000	1.00

Significant enrichment of repeat subfamilies in PD versus control (CO) groups was assessed using Fisher’s exact test. “Significant in PD (*n*)” and “Significant in CO (*n*)” indicate the number of repeat subfamilies differentially expressed (padj < 0.05) in each group. “Total TEs (*n*)” represents the total number of annotated subfamilies within each category. Raw *p*-values were adjusted for multiple testing using the Benjamini–Hochberg method. Bold values indicate statistically significant enrichment after FDR correction (adjusted *p* < 0.05).

## Data Availability

The original contributions presented in this study are included in the article/Supplementary Materials. Further inquiries can be directed to the corresponding authors.

## Supplementary Materials

The online version contains supplementary materials available at https://www.mdpi.com/article/10.3390/genes17050577/s1. Supplementary Material 1. Figure S1: Six Supplementary Figure S1a–s of differential expression of repeatome family and subfamily elements in peripheral blood across five timepoints (BL to V08) presented as line plots showing average expression and log2 fold change and heatmaps of log2 fold change in expression; a, 15 Alu; b, two CR1 DNA; c, 74 ERV1; d, 11 ERV2; e, 28 ERV3; f, 10 hAT DNA; g, 17 L1; h, one L2; i, one L2-end; j, two LTR; k, 9 Mariner_Tc1 DNA; l, 11 other DNA elements; m, 6 SVA; n, 7 satellite tandem repeats; o, 20 simple tandem repeats; p, 10 rRNA pseudogenes; q, five scRNA pseudogenes; r, five snRNA pseudogenes; s, six tRNA pseudogenes; Figure S2: Temporal expression dynamics of repeatome classes in peripheral blood cells of PD v BL (a) and CO v BL (b) groups. Each panel represents a distinct transposable element class (e.g., DNA transposons, ERV & LTR, LINEs, SINEs, SVAs, pseudogenes, and tandem/interspersed repeats). Points indicate the mean log_2_ fold change in expression at each time point (V02-6 months, V04-12 months, V06-24 months, and V08-36 months), with vertical bars showing the standard deviation (SD). Red triangles denote statistically significant differential expression (*p* < 0.05), while black circles indicate non-significant changes. Sample size (*n*) is displayed above each point. These trends summarize the evolving expression landscape of major repeat classes across time; Figure S3a–m: Temporal changes in expression of different repeat subfamilies in peripheral blood cells of PD vs. BL and CO vs. BL groups at four timepoints (V02, V04, V06, V08) relative to baseline (BL). Line plots show the log_2_ fold change in expression of the following repeat subfamilies: a, Alu; b, CR1; c, ERV1; d, ERV2; e, ERV3; f, hAT; g, L1; h, Mariner_Tc1; i, other-DNA; j, satellite; k, simple repeats; l, SVA; and m, tRNA. Each facet represents a different Repeat subfamily member. Only elements that showed a statistically significant change (adjusted *p* < 0.05) at one or more timepoints are included; Figure S4a–p: Heatmaps for differential expression patterns of 15 different repeat subfamilies in peripheral blood cells of PD vs. BL and CO vs. BL groups at visits V02 (6 months), V04 (12 months), V06 (24 months), and V08 (36 months): a, Alus; b, CR1s, c, ERV1s, d, ERV2s; e, ERV3s; f, hATs; g, L1s; h, Mariner_Tc1s; i, Other-DNA elements; j, rRNAs, k, Satellites; l, scRNAs; m, Simple repeats; n, snRNAs; o, SVAs; and p, tRNAs. Heatmaps show log_2_ fold change in expression for each element at each timepoint with a significant change in expression at least at one visit; Supplementary Material 2. Table S1: Key clinical covariates available from the PPMI cohort; Table S2: PD versus CO: 5947 PPMI salmon differentially expressed TEs across five timepoints; Table S3: PD versus CO: Repeat subfamily (name) elements (*n* = 259) expressed (Log2foldchange) significantly (padj < 0.05) at least; Table S4: PD versus CO: 120 PPMI differentially expressed ERVs across five timepoints at padj < 0.05; Table S5: PD versus BL_All_TEs_Combined_4791; Table S6: CO versus BL_All_TEs_Combined_4755; Table S7: Venn analysis with significantly expressed TEs detected and annotated by SalmonTE software in the PDs and COs at 4 timepoints relative to BL data: Table S8: Locus-specific differential expression of transposable elements in baseline female subset. Supplementary Material 3. R_Scripts.

## Abbreviations

The following abbreviations are used in this manuscript:

7SK	Small non-coding RNA 7SK
7SL	Small non-coding RNA, 7SL, component of signal recognition particle
Alu	Arthrobacter luteus
ATAC-seq	Assay for Transposase-Accessible Chromatin using sequencing
BL	Baseline (time of initial sample collection)
CO	Healthy control participants
CNS	Central nervous system
CR1	Chicken repeat 1
CSF	Cerebrospinal fluid
DE	Differential expression
dsRNA	Double-stranded RNA
eQTL	Expression quantitative trait locus
ERV	Endogenous retrovirus
FDR	False discovery rate
GBA	Acid beta-glucocerebrosidase
HY1/HY3	Human Y small RNA pseudogenes
IFN-I	Interferon type 1
LINE/L1	Long Interspersed Nuclear Element/LINE-1
LRKK2	Leucine-rich repeat kinase 2
MAPT	Microtubule-associated protein tau gene
MIR	Mammalian-wide interspersed repeat
MLT	Mammalian LTR retro-transposon
padj	Adjusted *p*-value
PD	Parkinson’s disease
PPMI	Parkinson’s Progression Markers Initiative
PRKN	Parkin gene or protein
rRNA	Ribosomal RNA
RNA-seq	RNA sequencing
scRNA	Small cytoplasmic RNA
Seq	Sequence
snRNA	Small nuclear RNA
SNCA	Synuclein alpha gene
sncRNA	Small noncoding RNA
SINE	Short Interspersed Nuclear Element
SVA	SINE-VNTR-Alu composite element
TE	Transposable element
tRNA	Transfer RNA
V02	Visit 2 (6 months from baseline)
V04	Visit 4 (12 months from baseline)
V06	Visit 6 (24 months from baseline)
V08	Visit 8 (36 months from baseline)
vs.	Versus
LTR	Long terminal repeat element
Y1/Y3/Y4	Y subfamily of small non-coding RNAs and components of Ro60 ribonucleoprotein particle
