# Supplementary material for: Longitudinal Repeatome Remodeling in Peripheral Blood Following Parkinson’s Disease Diagnosis
Source: Genes (Basel). 2026 May 18;17(5):577. doi: 10.3390/genes17050577 (PMC13206192; doi:10.3390/genes17050577)
Supplement: Supplementary file 1 [file genes-17-00577-s001.zip › 1.Supplementary Material1_Figures/Supplementary Figures S1-S4.docx]

**S1a**


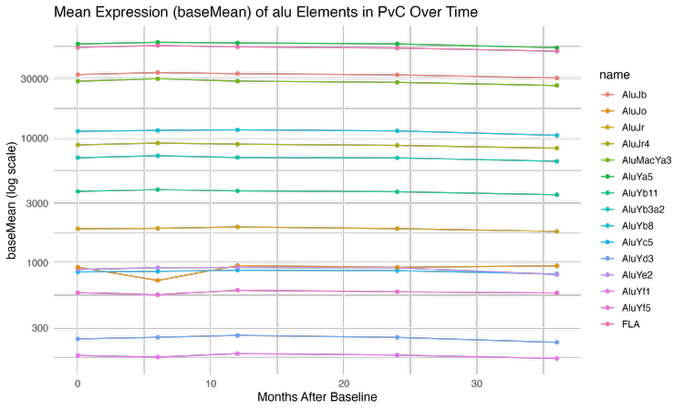

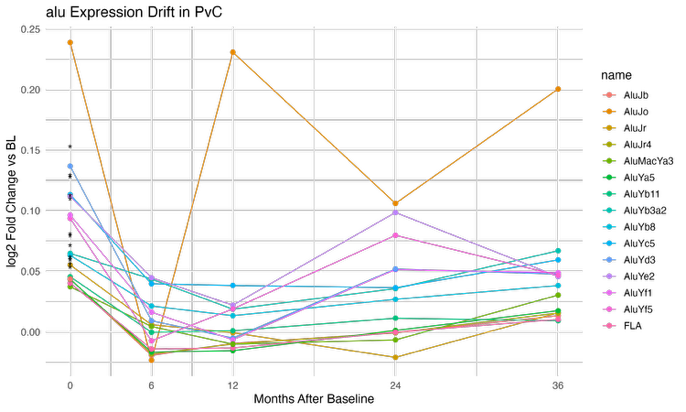


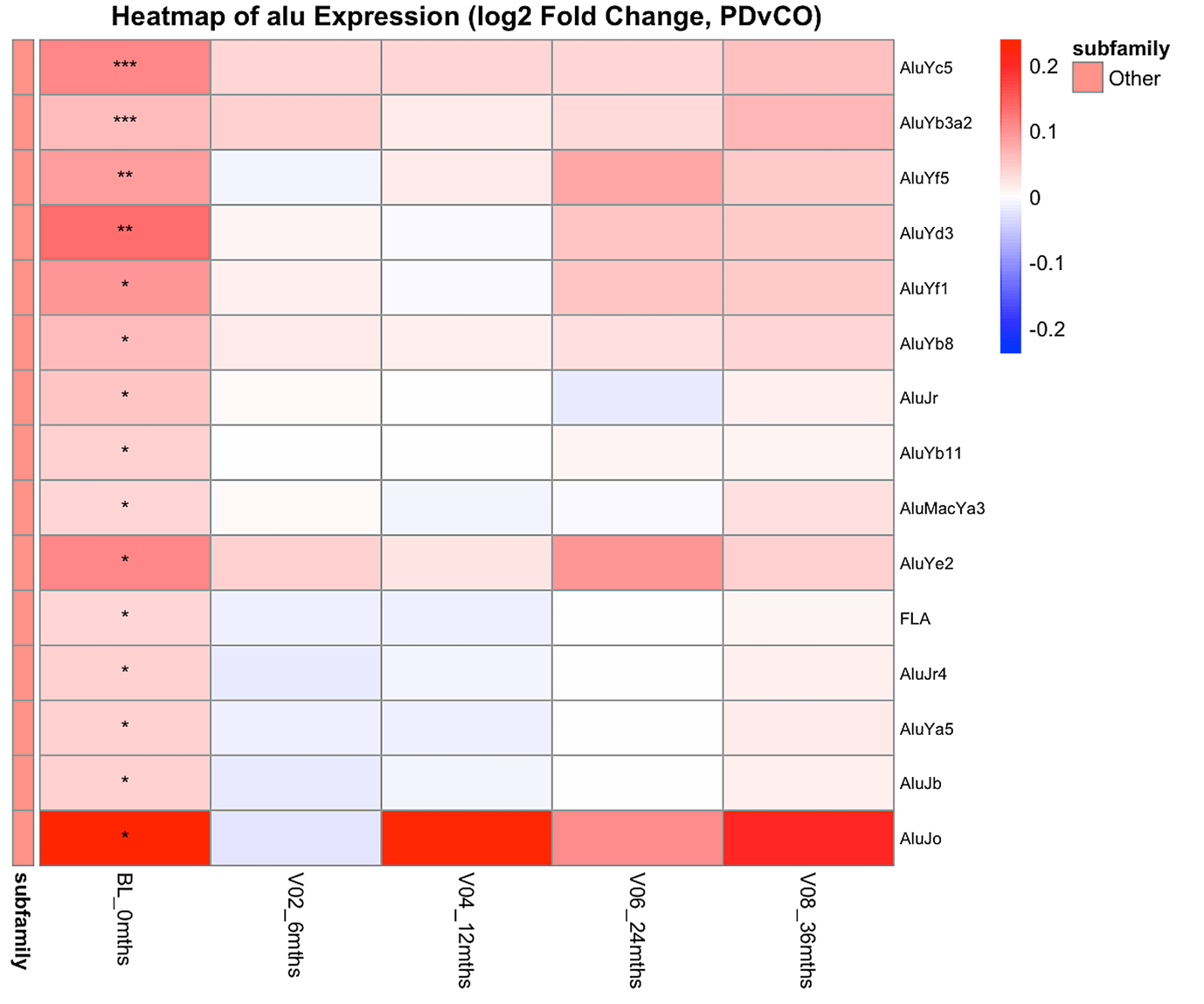


**Figure S1a.**
Differential expression for fifteen Alu SINE elements in peripheral blood cells of Parkinson’s disease (PD) patients versus healthy controls (CO) across five timepoints: baseline (BL), 6 months (V02), 12 months (V04), 24 months (V06), and 36 months (V08).
Top left: Line plot showing the average expression (baseMean, log₁₀ scale) of the fifteen Alu elements across timepoints (x-axis: months after BL).
Top right: Line plot showing log₂ fold change (y-axis) in Alu expression relative to baseline over the same timepoints.
Bottom: Heatmap of log₂ fold change in expression for each Alu element at each timepoint.
Asterisks indicate statistical significance: padj < 0.05 (*), padj < 0.01 (**), padj < 0.001 (***).

**S1b**


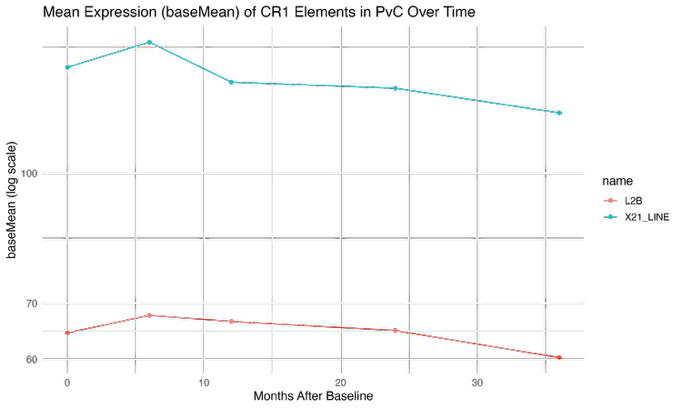

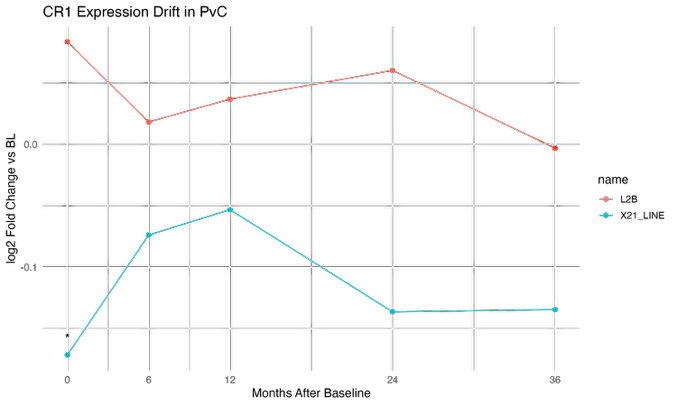


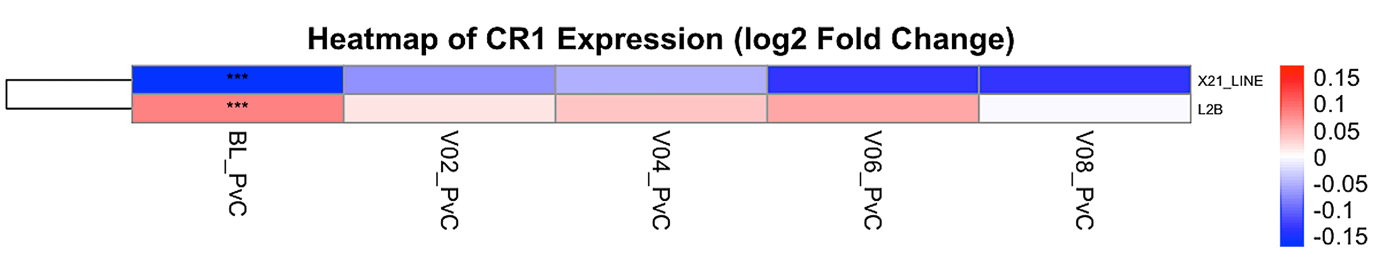


**Figure S1b.**
Differential expression of two CR1 DNA elements in peripheral blood cells of Parkinson’s disease (PD) patients versus healthy controls across five timepoints: baseline (BL), 6 months (V02), 12 months (V04), 24 months (V06), and 36 months (V08).
Top left: Line plot showing the average expression (baseMean, log₁₀ scale) of the two CR1 elements across timepoints (x-axis: months after BL).
Top right: Line plot showing log₂ fold change (y-axis) in CR1 expression relative to baseline over the same timepoints.
Bottom: Heatmap of log₂ fold change in expression for each CR1 element at each timepoint.
Asterisks indicate statistical significance: padj < 0.05 (*), padj < 0.01 (**), padj < 0.001 (***).

**S1c**


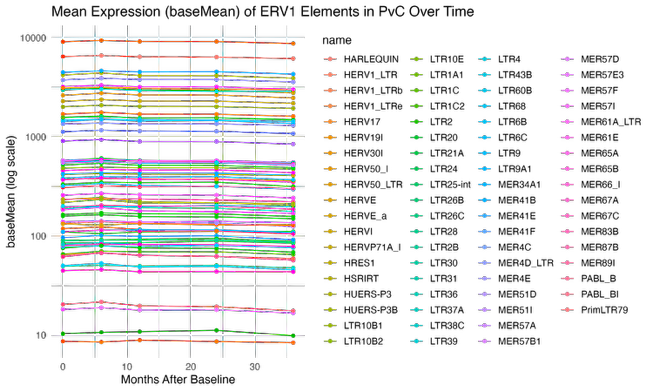

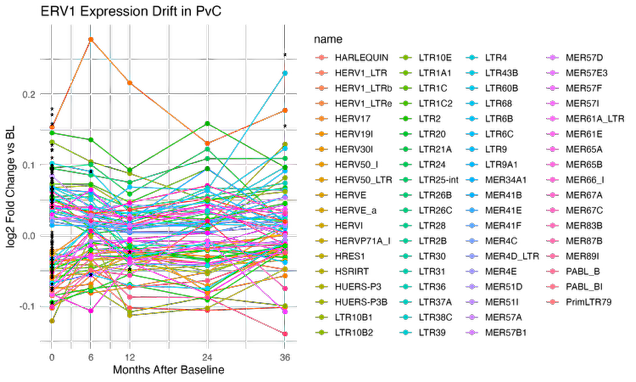


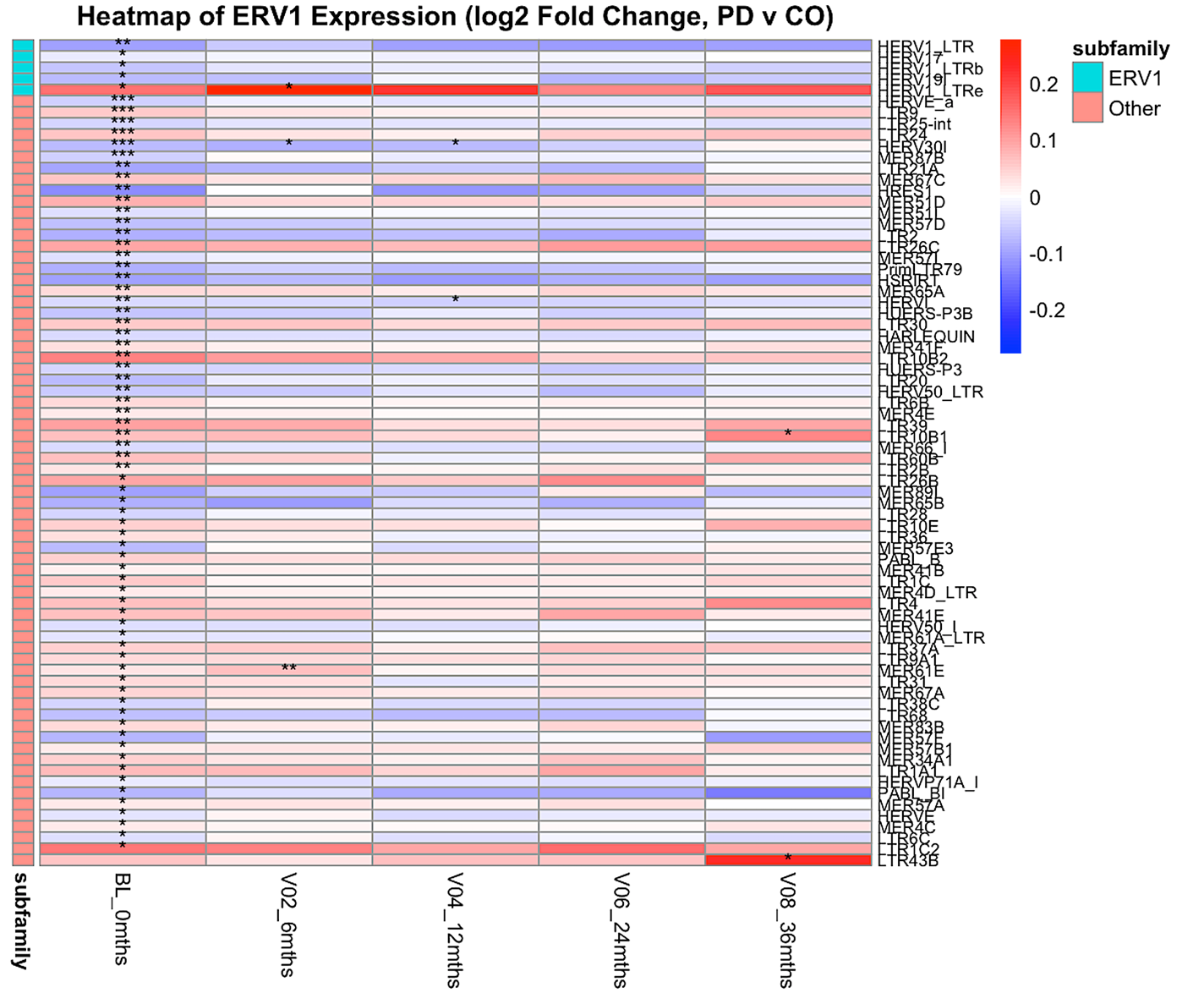


**Figure S1c.**
Differential expression of 74 ERV1 elements in peripheral blood cells of Parkinson’s disease (PD) patients versus healthy controls across five timepoints: baseline (BL), 6 months (V02), 12 months (V04), 24 months (V06), and 36 months (V08).
Top left: Line plot showing the average expression (baseMean, log₁₀ scale) of the 74 ERV1 elements across timepoints (x-axis: months after BL).
Top right: Line plot showing log₂ fold change (y-axis) in ERV1 expression relative to baseline over the same timepoints.
Bottom: Heatmap of log₂ fold change in expression for each ERV1 element at each timepoint.
Asterisks indicate statistical significance: padj < 0.05 (*), padj < 0.01 (**), padj < 0.001 (***).

**S1d**


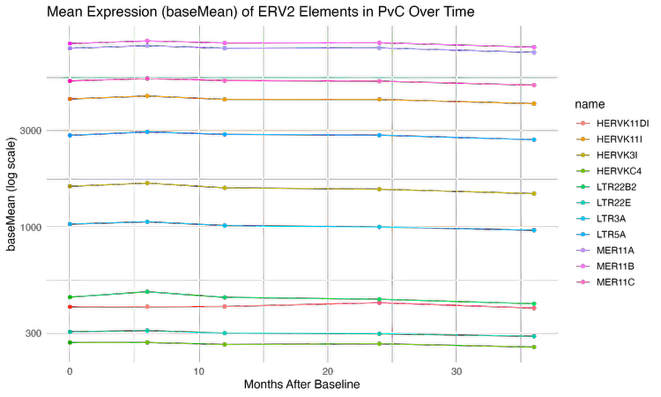

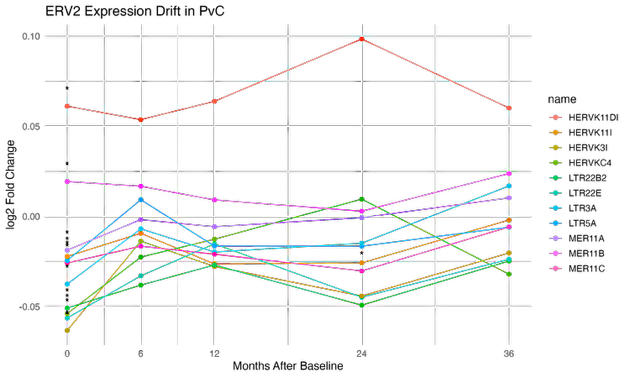


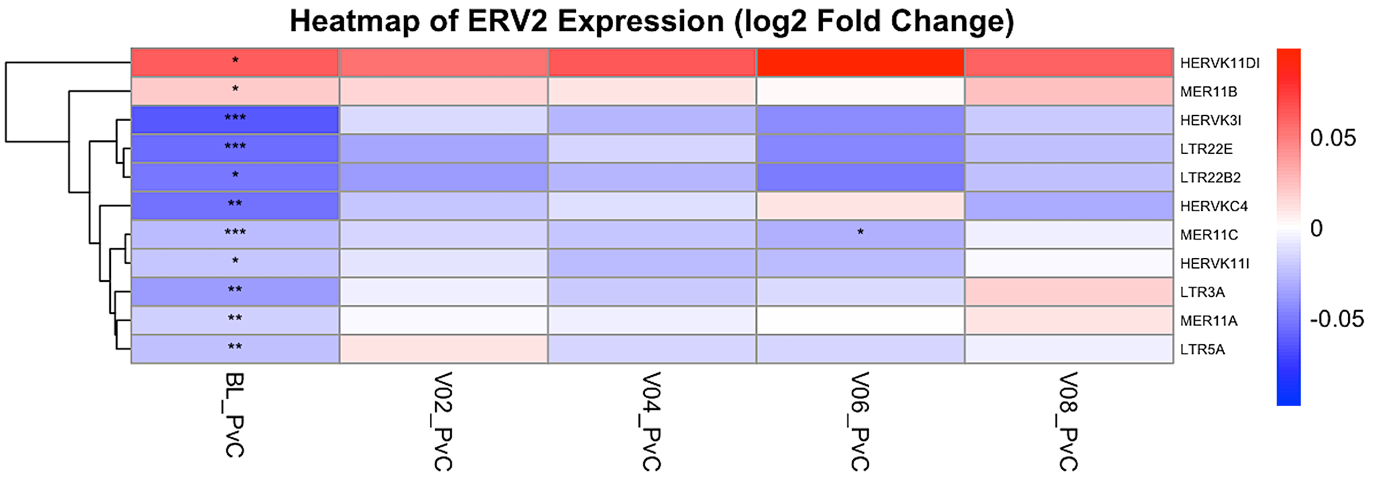


**Figure S1d.**
Differential expression of 11 ERV2 elements in peripheral blood cells of Parkinson’s disease (PD) patients versus healthy controls across five timepoints: baseline (BL), 6 months (V02), 12 months (V04), 24 months (V06), and 36 months (V08).
Top left: Line plot showing the average expression (baseMean, log₁₀ scale) of the 11 ERV2 elements across timepoints (x-axis: months after BL).
Top right: Line plot showing log₂ fold change (y-axis) in ERV2 expression relative to baseline over the same timepoints.
Bottom: Heatmap of log₂ fold change in expression for each ERV2 element at each timepoint.
Asterisks indicate statistical significance: padj < 0.05 (*), padj < 0.01 (**), padj < 0.001 (***).

**S1e**


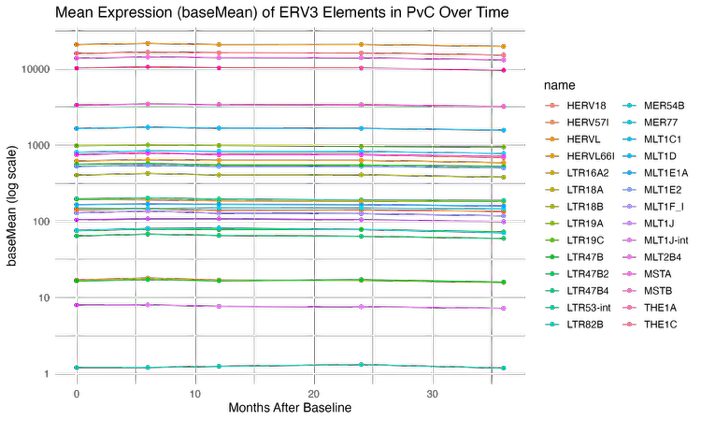

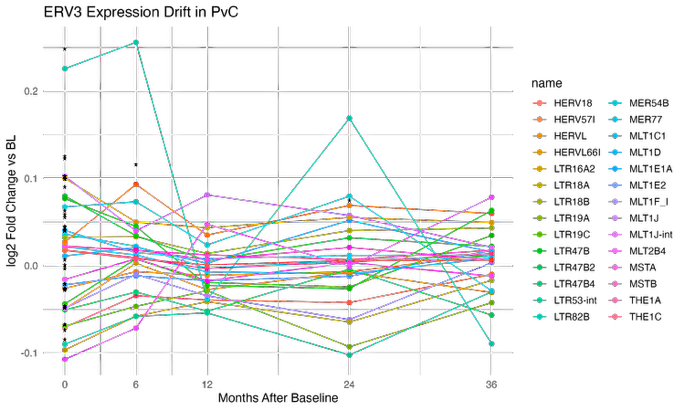


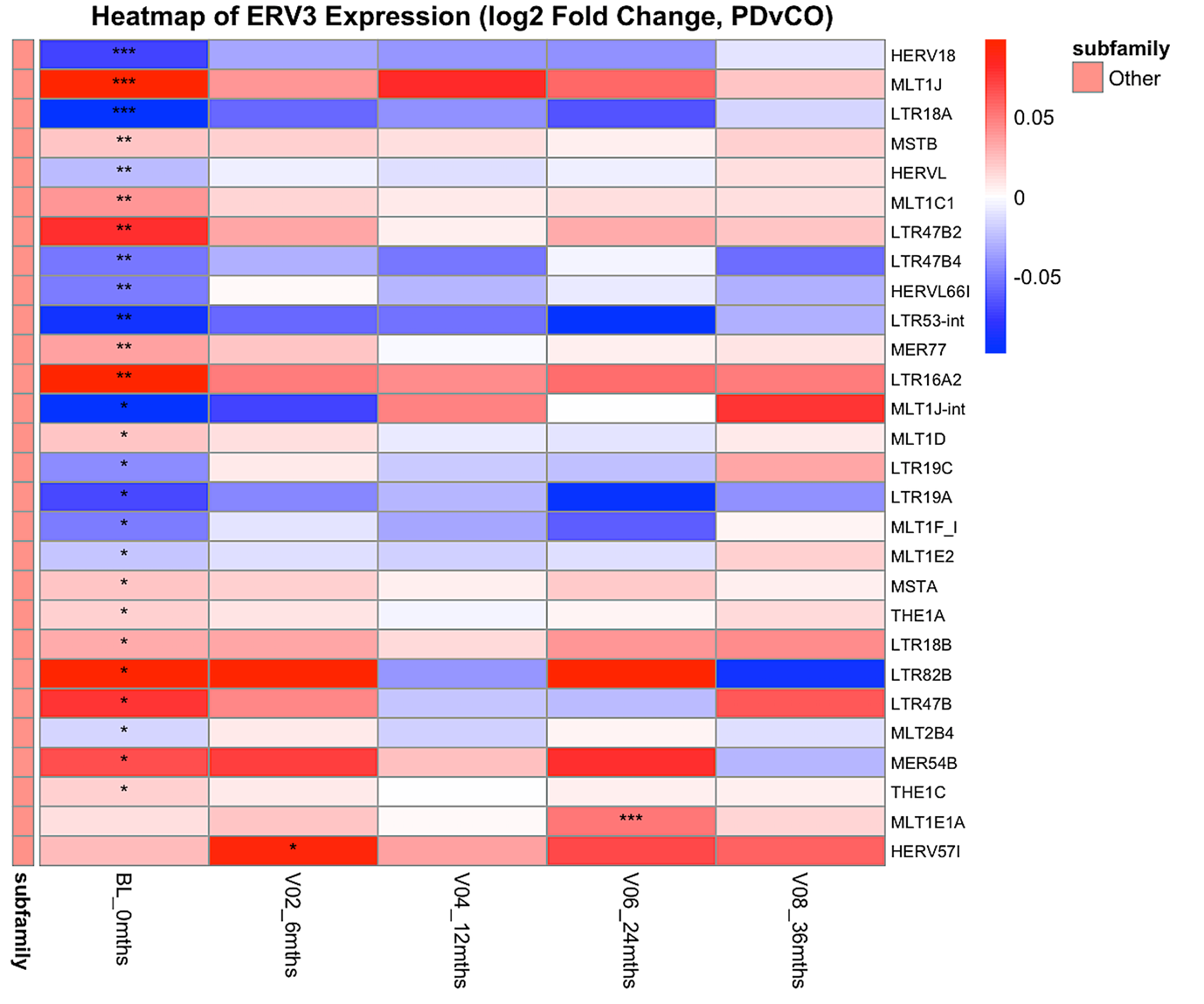


**Figure S1e.**
Differential expression of 28 ERV3 elements in peripheral blood cells of Parkinson’s disease (PD) patients versus healthy controls across five timepoints: baseline (BL), 6 months (V02), 12 months (V04), 24 months (V06), and 36 months (V08).
Top left: Line plot showing the average expression (baseMean, log₁₀ scale) of the 28 ERV3 elements across timepoints (x-axis: months after BL).
Top right: Line plot showing log₂ fold change (y-axis) in ERV3 expression relative to baseline over the same timepoints.

**S1f**
Bottom: Heatmap of log₂ fold change in expression for each ERV3 element at each timepoint.
Asterisks indicate statistical significance: padj < 0.05 (*), padj < 0.01 (**), padj < 0.001 (***).


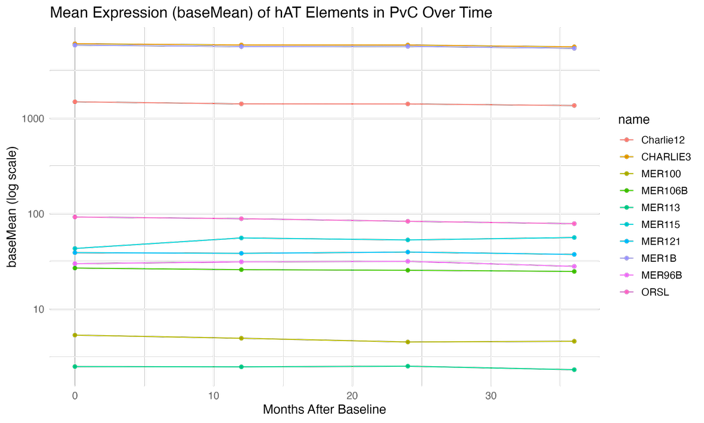

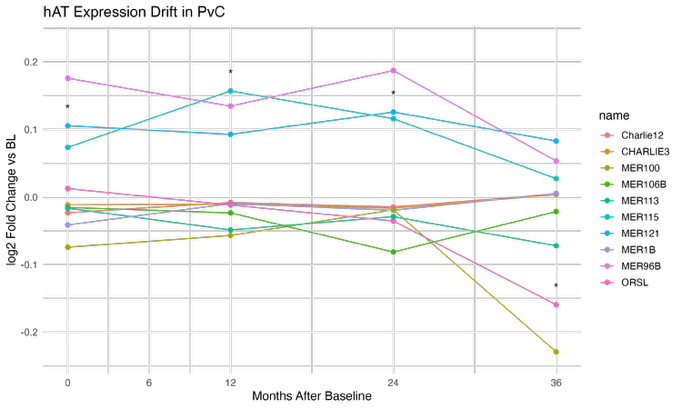


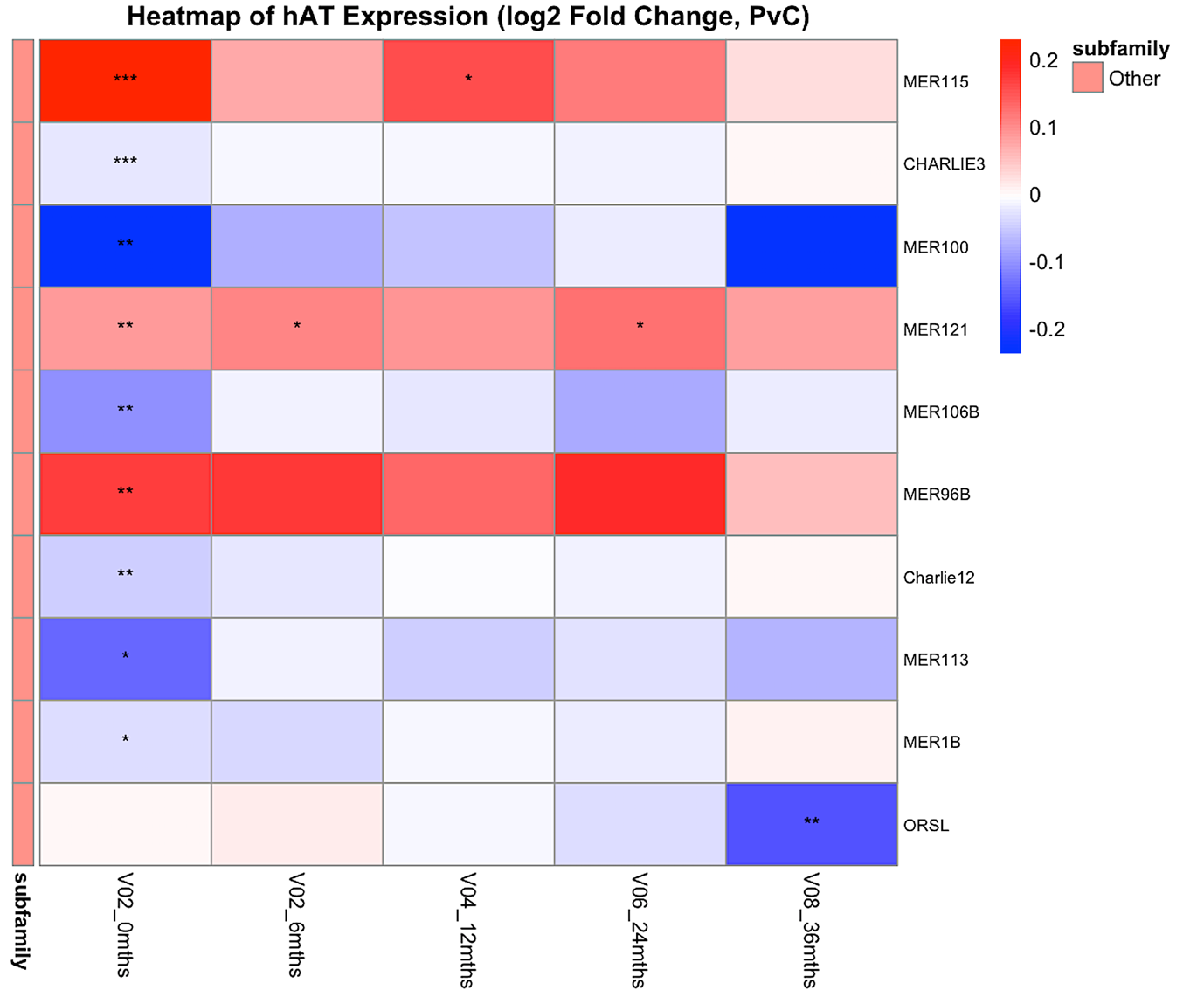


**Figure S1f.**
Differential expression of 10 hAT DNA elements in peripheral blood cells of Parkinson’s disease (PD) patients versus healthy controls across five timepoints: baseline (BL), 6 months (V02), 12 months (V04), 24 months (V06), and 36 months (V08).
Top left: Line plot showing the average expression (baseMean, log₁₀ scale) of the 10 hAT DNA elements across timepoints (x-axis: months after BL).
Top right: Line plot showing log₂ fold change (y-axis) in hAT expression relative to baseline over the same timepoints.
Bottom: Heatmap of log₂ fold change in expression for each hAT element at each timepoint.
Asterisks indicate statistical significance: padj < 0.05 (*), padj < 0.01 (**), padj < 0.001 (***).

**S1g**


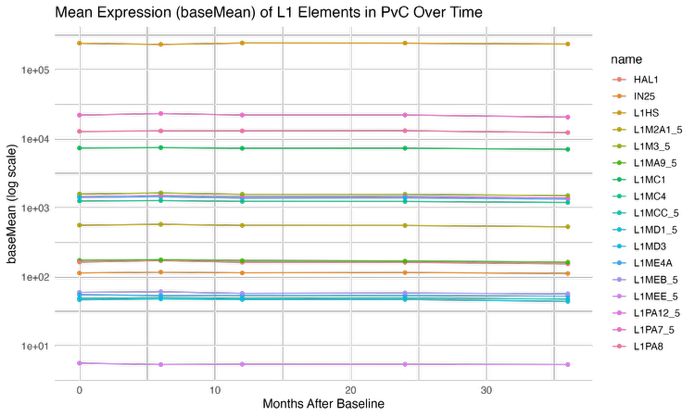

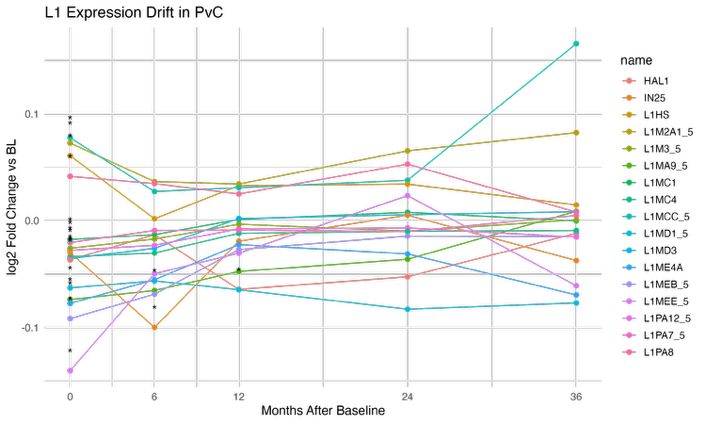


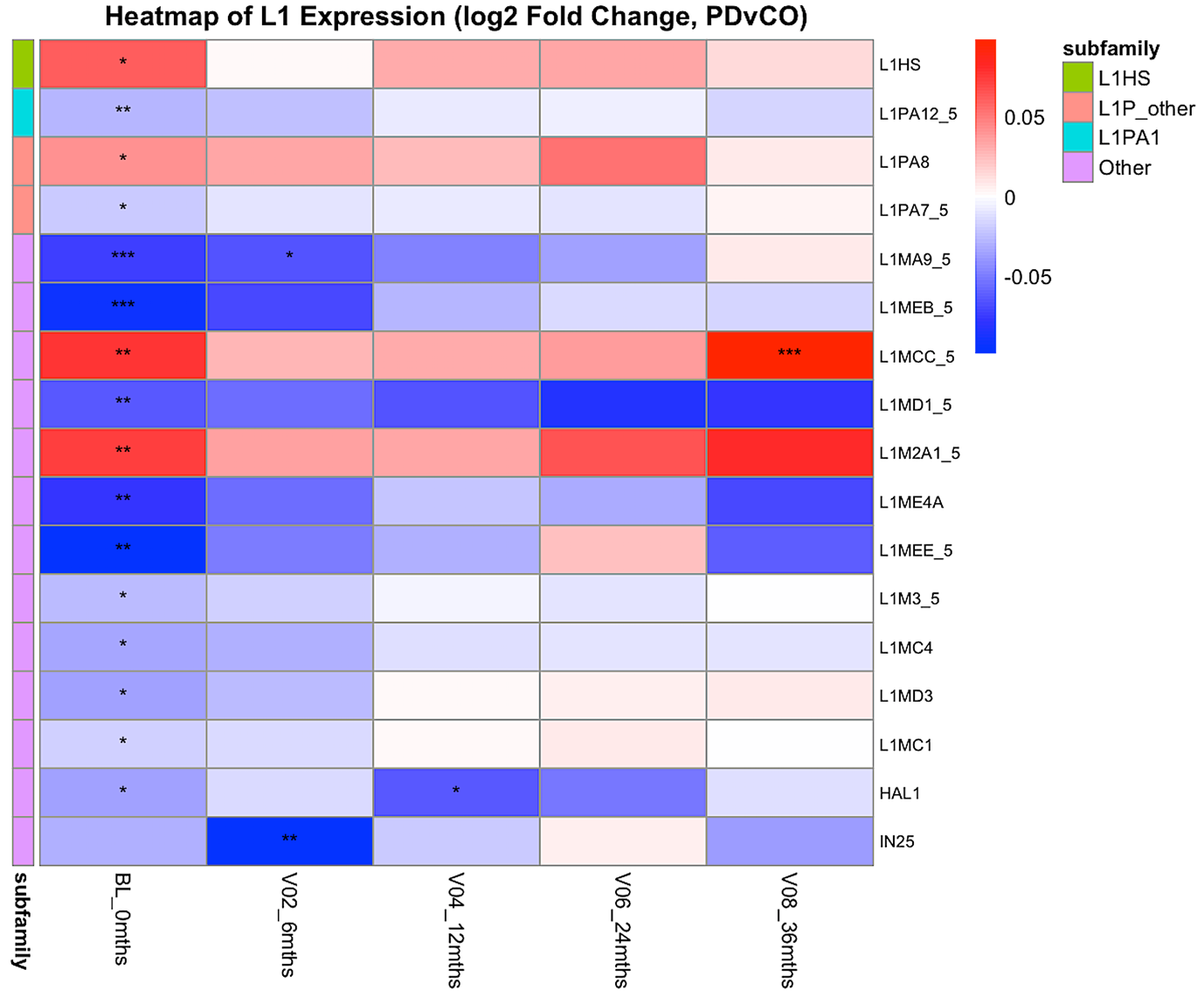


**Figure S1g.**
Differential expression of 17 L1 elements in peripheral blood cells of Parkinson’s disease (PD) patients versus healthy controls across five timepoints: baseline (BL), 6 months (V02), 12 months (V04), 24 months (V06), and 36 months (V08).
Top left: Line plot showing the average expression (baseMean, log₁₀ scale) of the 17 L1 elements across timepoints (x-axis: months after BL).
Top right: Line plot showing log₂ fold change (y-axis) in L1 expression relative to baseline over the same timepoints.
Bottom: Heatmap of log₂ fold change in expression for each L1 element at each timepoint.
Asterisks indicate statistical significance: padj < 0.05 (*), padj < 0.01 (**), padj < 0.001 (***).

**S1h**


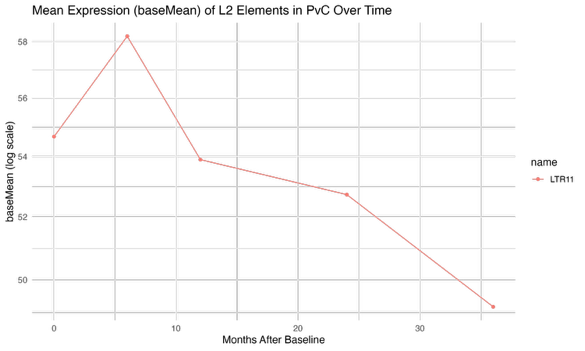

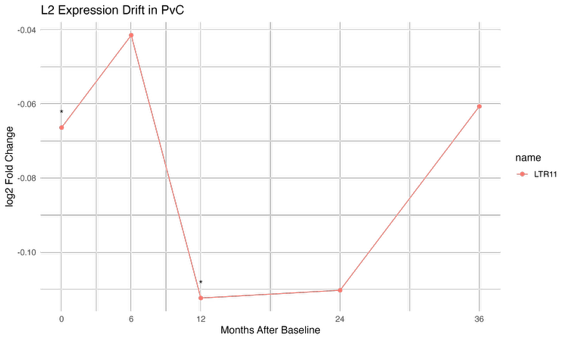


**Figure S1h.**
Differential expression of one L2 element, LTR11, in peripheral blood cells of Parkinson’s disease (PD) patients versus healthy controls across five timepoints: baseline (BL), 6 months (V02), 12 months (V04), 24 months (V06), and 36 months (V08).Top left: Line plot showing the average expression (baseMean, log₁₀ scale) of LTR11 across timepoints (x-axis: months after BL). Top right: Line plot showing log₂ fold change (y-axis) in LTR11 expression relative to baseline over the same timepoints. Asterisks indicate statistical significance: padj < 0.05 (*), padj < 0.01 (**), padj < 0.001 (***).

**S1i**


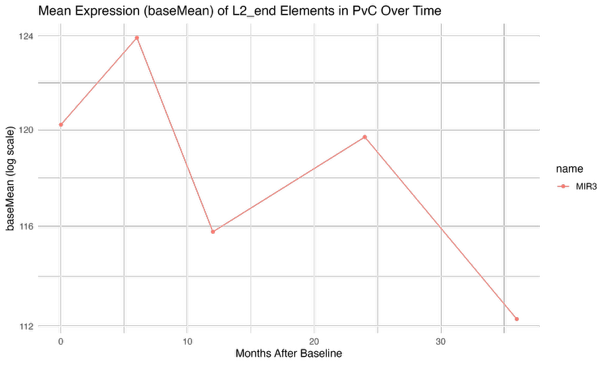

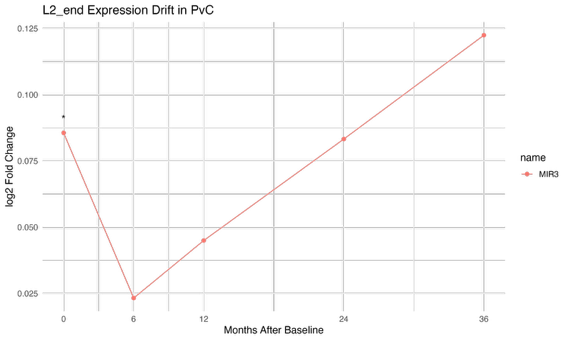


**Figure S1i.**
Differential expression of one L2-end element, MIR3, in peripheral blood cells of Parkinson’s disease (PD) patients versus healthy controls across five timepoints: baseline (BL), 6 months (V02), 12 months (V04), 24 months (V06), and 36 months (V08). Top left: Line plot showing the average expression (baseMean, log₁₀ scale) of MIR3 across timepoints (x-axis: months after BL). Top right: Line plot showing log₂ fold change (y-axis) in MIR3 expression relative to baseline over the same timepoints. Statistical significance (padj = 0.033) was at BL (0 months).

**S1j**


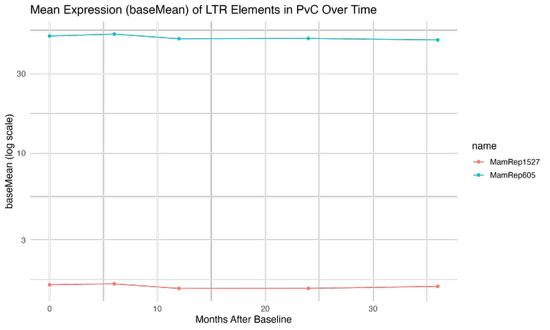

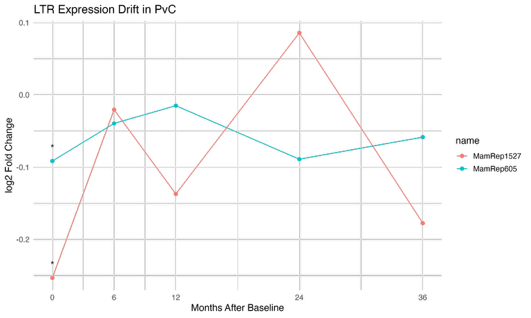


**Figure S1j.**
Differential expression of two LTR elements (MamRep1527, MamRep605)) in peripheral blood cells of Parkinson’s disease (PD) patients versus healthy controls across five timepoints: baseline (BL), 6 months (V02), 12 months (V04), 24 months (V06), and 36 months (V08). Top left: Line plot showing the average expression (baseMean, log₁₀ scale) of two LTR elements across timepoints (x-axis: months after BL). Top right: Line plot showing log₂ fold change (y-axis) in L1 expression relative to baseline over the same timepoints. Asterisks indicate statistical significance: padj < 0.05 (*).

**S1k**


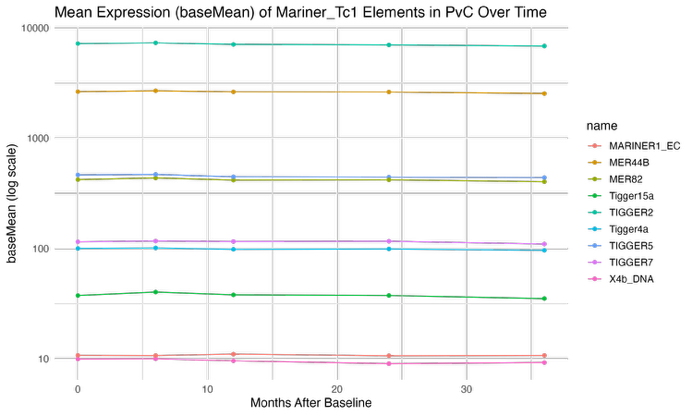

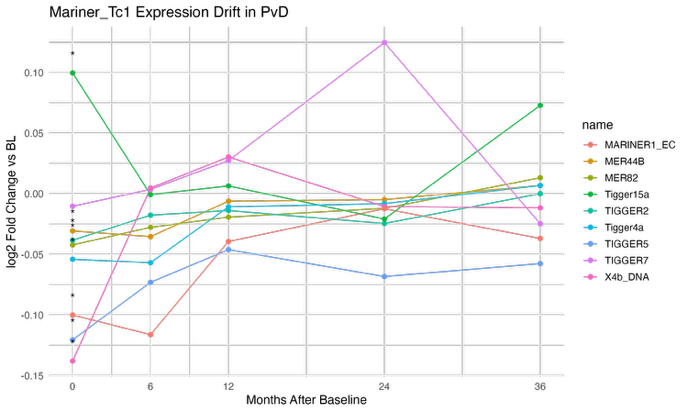


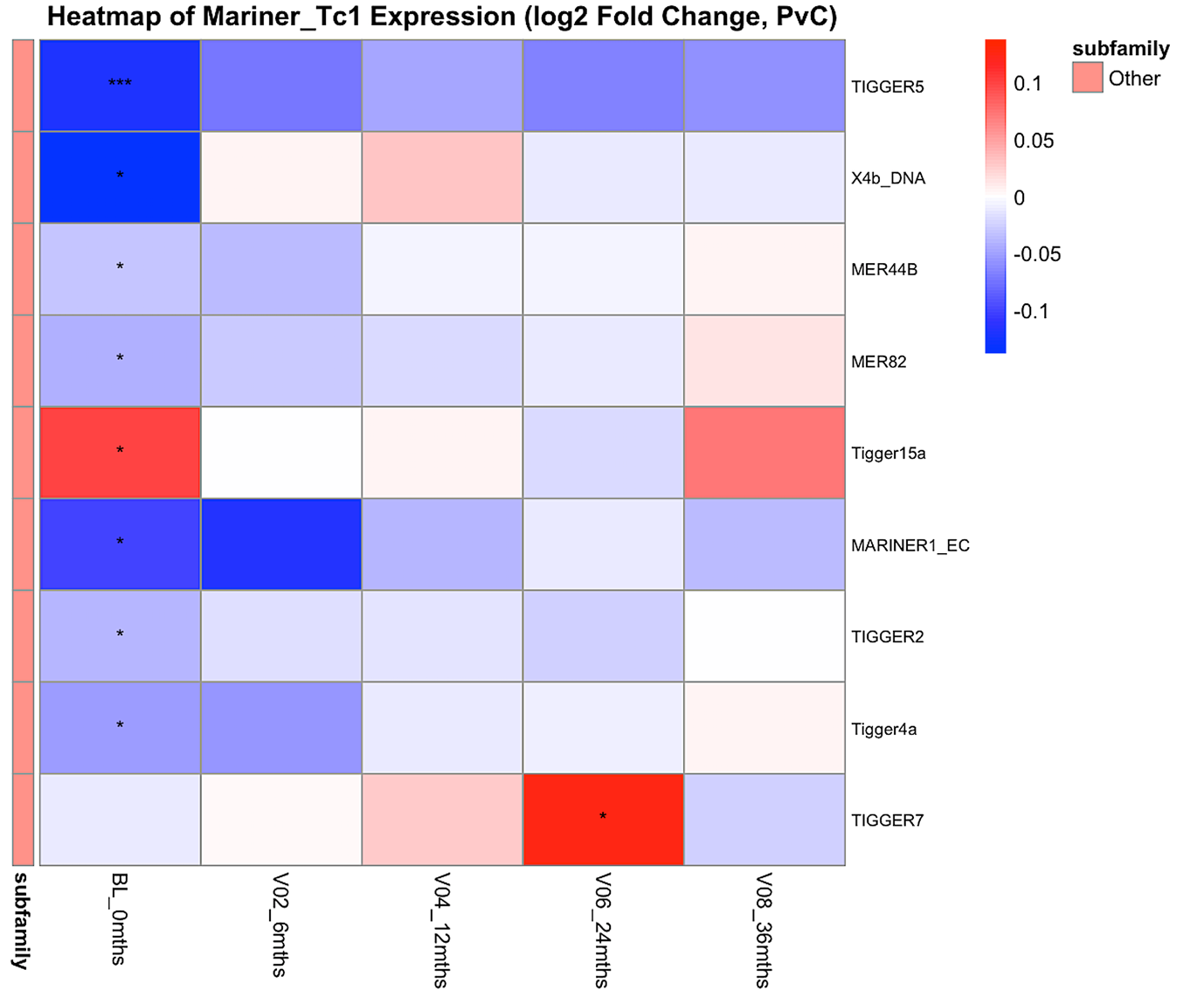


**Figure S1k.**
Differential expression of 9 Mariner_Tc1 DNA elements in peripheral blood cells of Parkinson’s disease (PD) patients versus healthy controls across five timepoints: baseline (BL), 6 months (V02), 12 months (V04), 24 months (V06), and 36 months (V08).
Top left: Line plot showing the average expression (baseMean, log₁₀ scale) of the 9 Mariner_Tc1 elements across timepoints (x-axis: months after BL).
Top right: Line plot showing log₂ fold change (y-axis) in Mariner_Tc1 expression relative to baseline over the same timepoints.
Bottom: Heatmap of log₂ fold change in expression for each Mariner_Tc1 element at each timepoint.
Asterisks indicate statistical significance: padj < 0.05 (*), padj < 0.01 (**), padj < 0.001 (***).

**S1l**


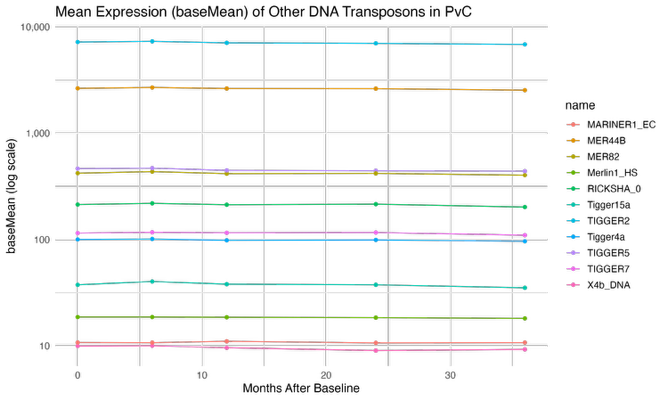

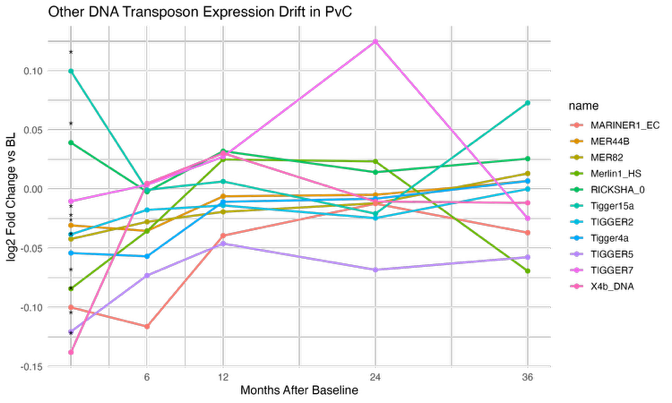


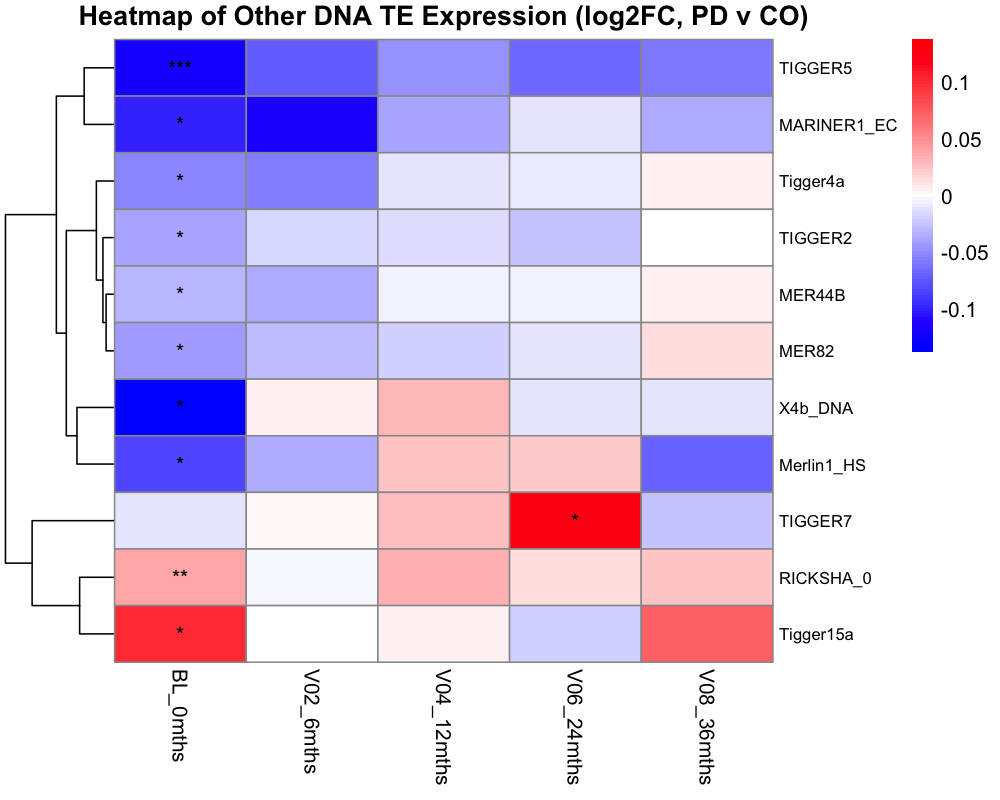


**Figure S1l.**
Differential expression of 11 Other DNA elements in peripheral blood cells of Parkinson’s disease (PD) patients versus healthy controls across five timepoints: baseline (BL), 6 months (V02), 12 months (V04), 24 months (V06), and 36 months (V08).
Top left: Line plot showing the average expression (baseMean, log₁₀ scale) of the 11 Other DNA elements across timepoints (x-axis: months after BL).
Top right: Line plot showing log₂ fold change (y-axis) in Other DNA expression relative to baseline over the same timepoints.
Bottom: Heatmap of log₂ fold change in expression for each Other DNA element at each timepoint.
Asterisks indicate statistical significance: padj < 0.05 (*), padj < 0.01 (**), padj < 0.001 (***).

**S1m**


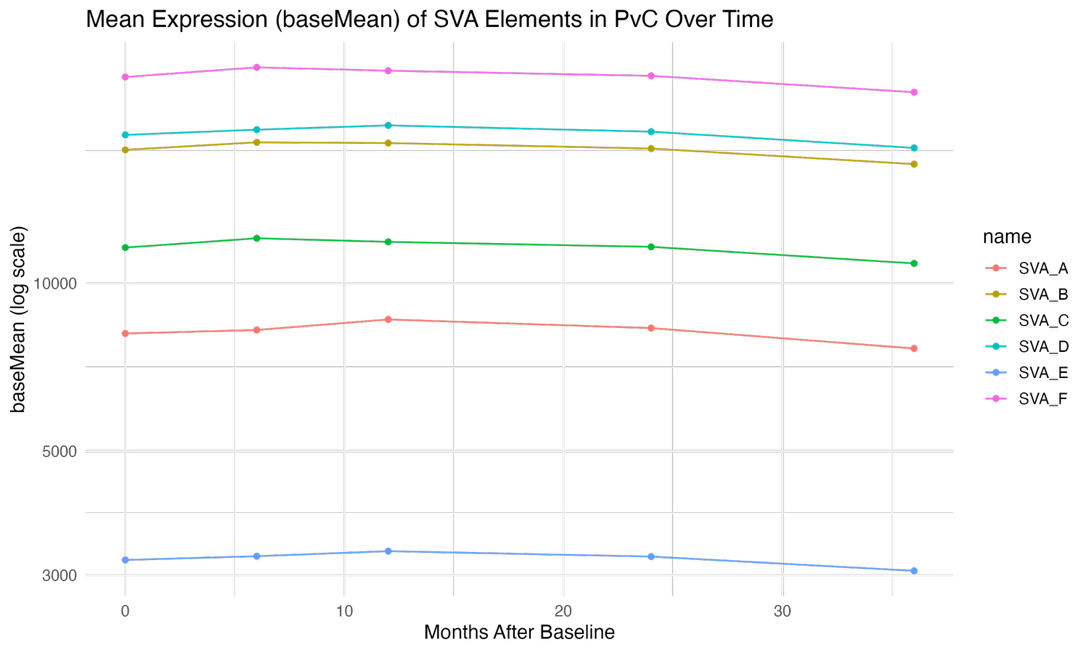


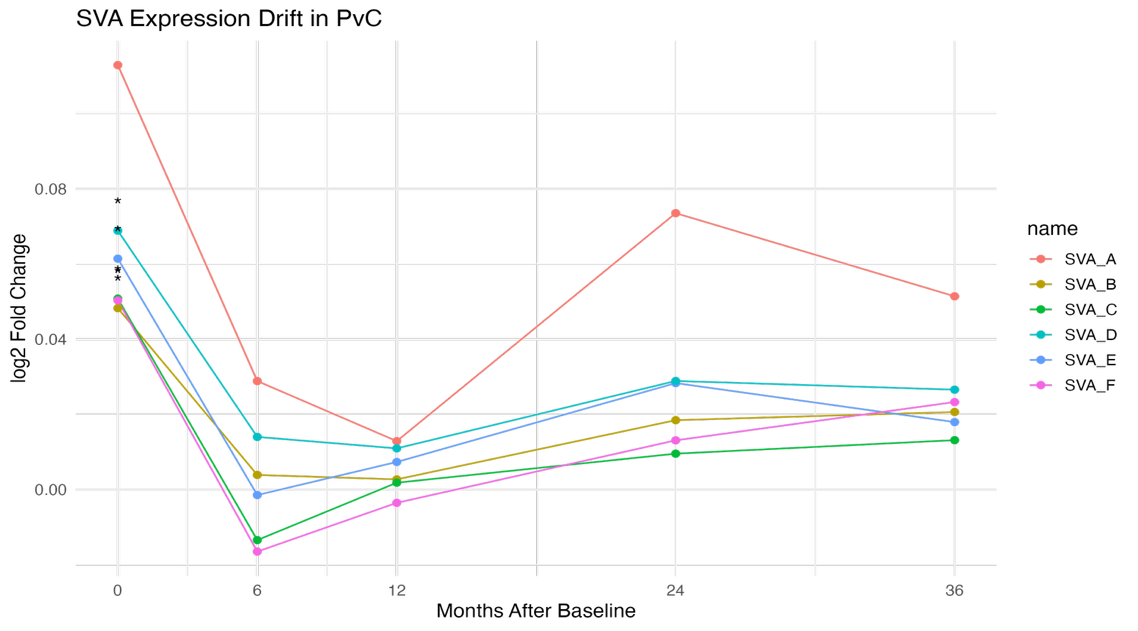


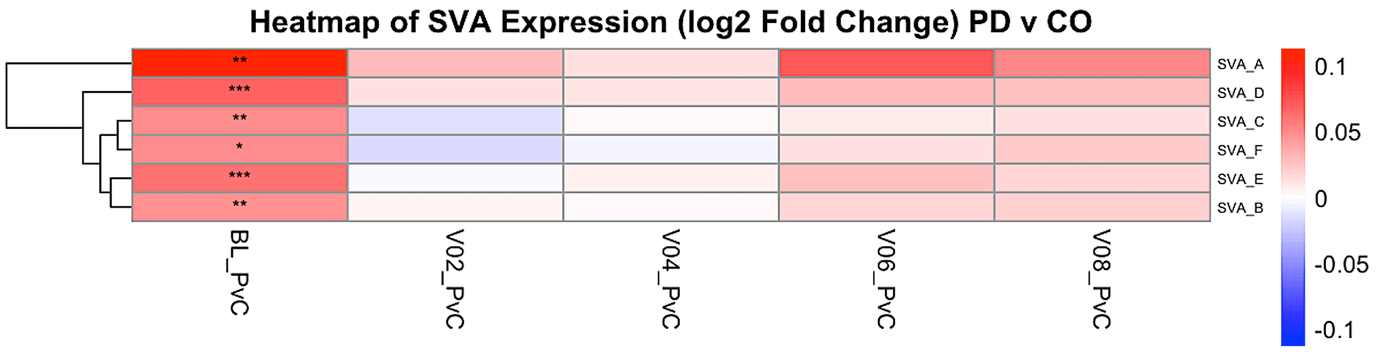


**Figure S1m.**
Differential expression of 6 SVA elements in peripheral blood cells of Parkinson’s disease (PD) patients versus healthy controls across five timepoints: baseline (BL), 6 months (V02), 12 months (V04), 24 months (V06), and 36 months (V08).
Top: Line plot showing the average expression (baseMean, log₁₀ scale) of the 6 SVA elements across timepoints (x-axis: months after BL).
Middle: Line plot showing log₂ fold change (y-axis) in SVA expression relative to baseline over the same timepoints.
Bottom: Heatmap of log₂ fold change in expression for each SVA element at each timepoint.
Asterisks indicate statistical significance: padj < 0.05 (*), padj < 0.01 (**), padj < 0.001 (***).

**S1n**


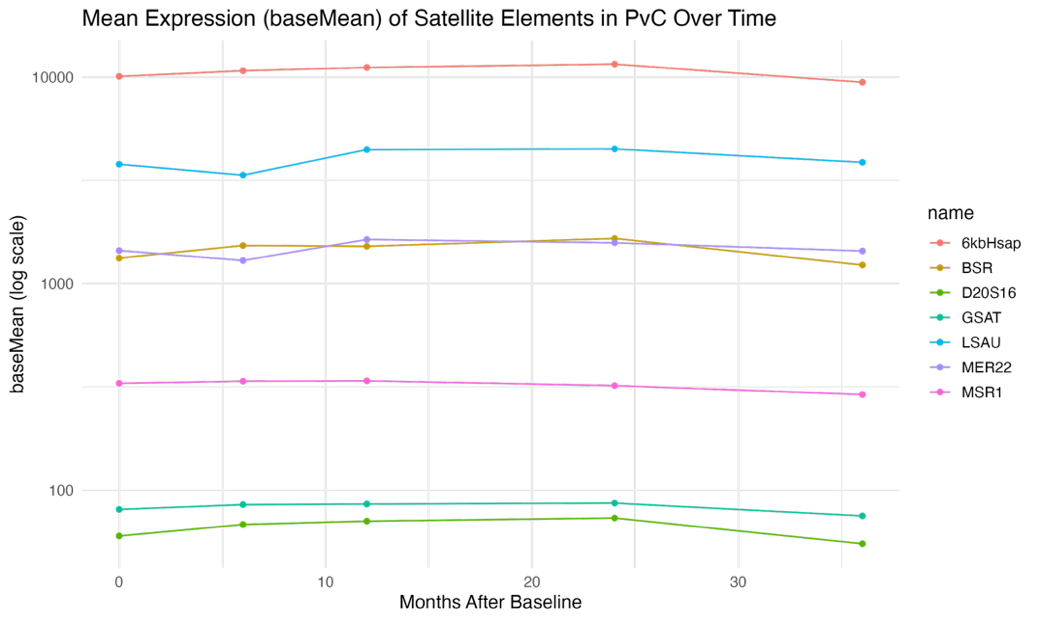


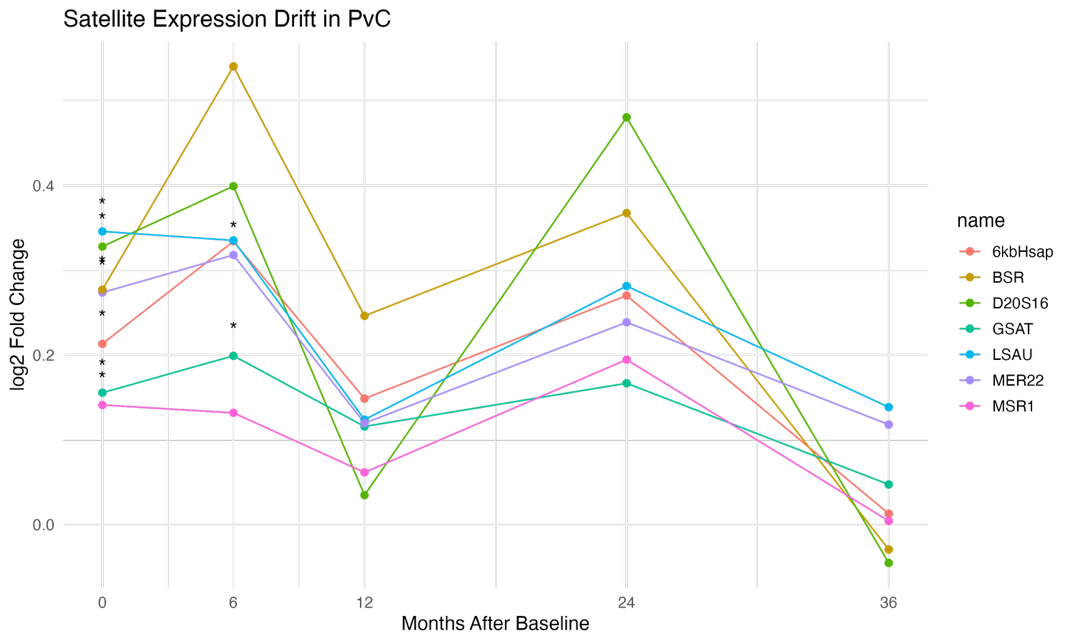


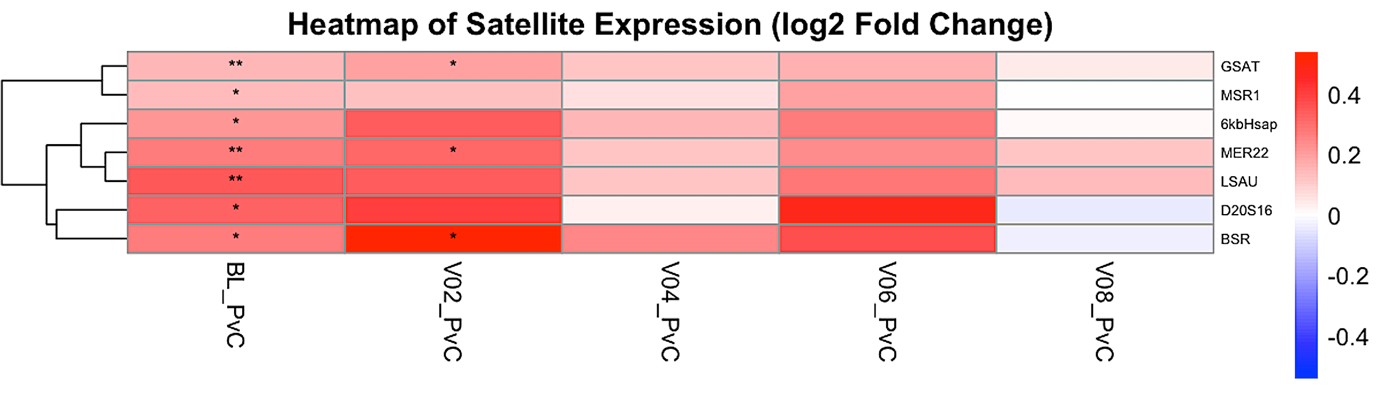


**Figure S1n.**
Differential expression of 7 Satellite Tandem Repeats in peripheral blood cells of Parkinson’s disease (PD) patients versus healthy controls across five timepoints: baseline (BL), 6 months (V02), 12 months (V04), 24 months (V06), and 36 months (V08).
Top: Line plot showing the average expression (baseMean, log₁₀ scale) of the 7 Satellites across timepoints (x-axis: months after BL).
Middle: Line plot showing log₂ fold change (y-axis) in Satellite expression relative to baseline over the same timepoints.
Bottom: Heatmap of log₂ fold change in expression for each Satellite element at each timepoint.
Asterisks indicate statistical significance: padj < 0.05 (*), padj < 0.01 (**), padj < 0.001 (***).

**S1o**


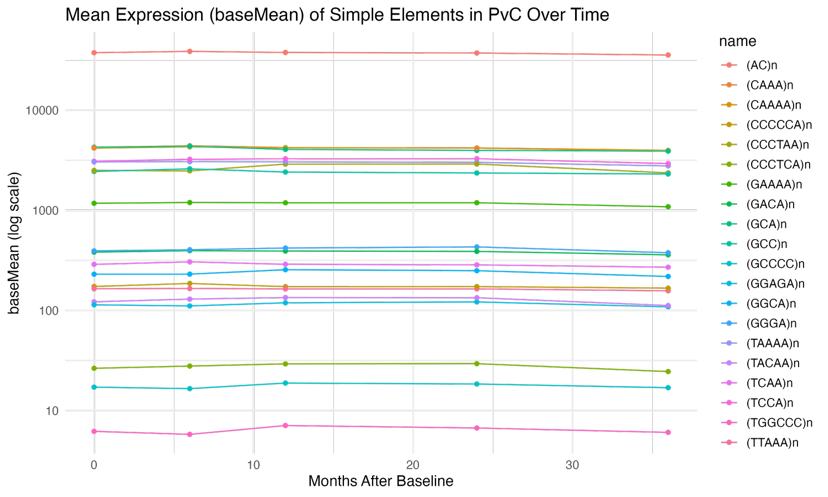


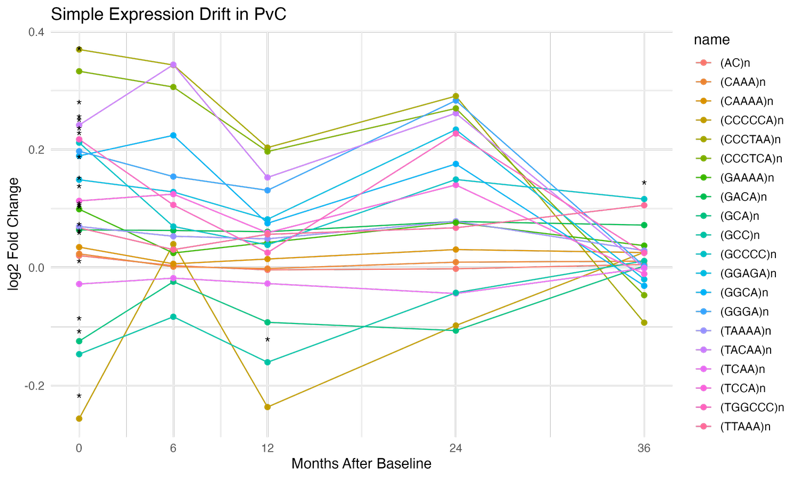


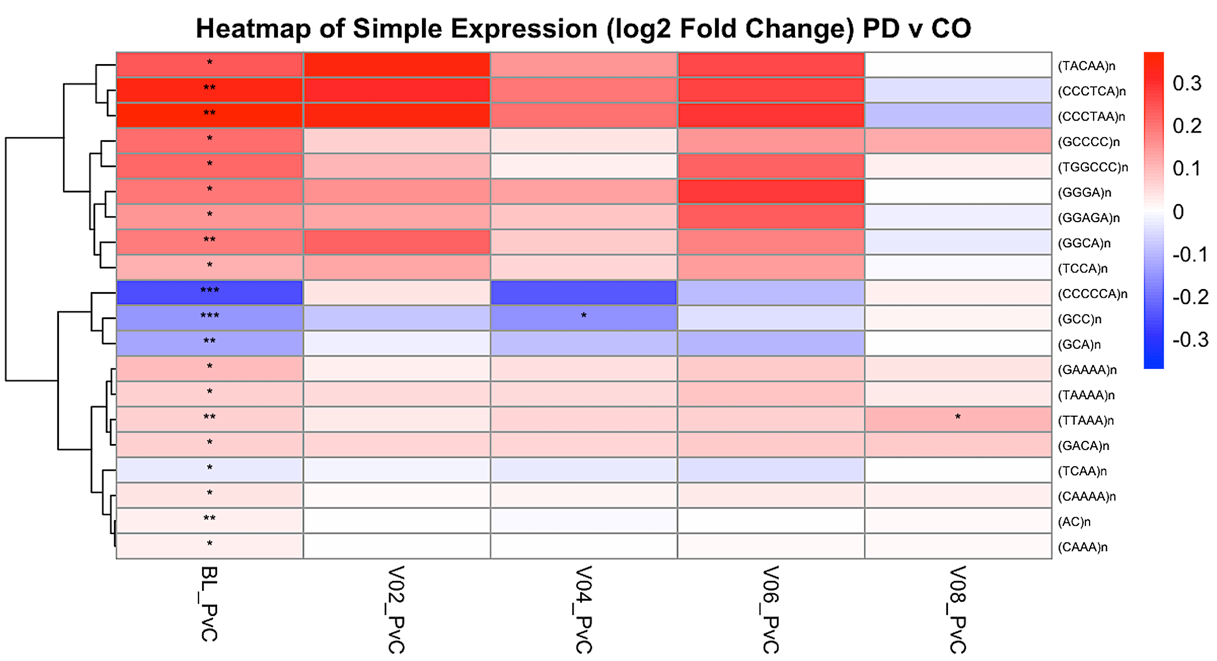


**Figure S1o.**
Differential expression of 20 Simple Tandem Repeats in peripheral blood cells of Parkinson’s disease (PD) patients versus healthy controls across five timepoints: baseline (BL), 6 months (V02), 12 months (V04), 24 months (V06), and 36 months (V08).
Top: Line plot showing the average expression (baseMean, log₁₀ scale) of the 20 Simple Tandem Repeats across timepoints (x-axis: months after BL).
Middle: Line plot showing log₂ fold change (y-axis) in Simple Tandem Repeats expression relative to baseline over the same timepoints.
Bottom: Heatmap of log₂ fold change in expression for each Simple Tandem Repeat at each timepoint.
Asterisks indicate statistical significance: padj < 0.05 (*), padj < 0.01 (**), padj < 0.001 (***).

**S1p**


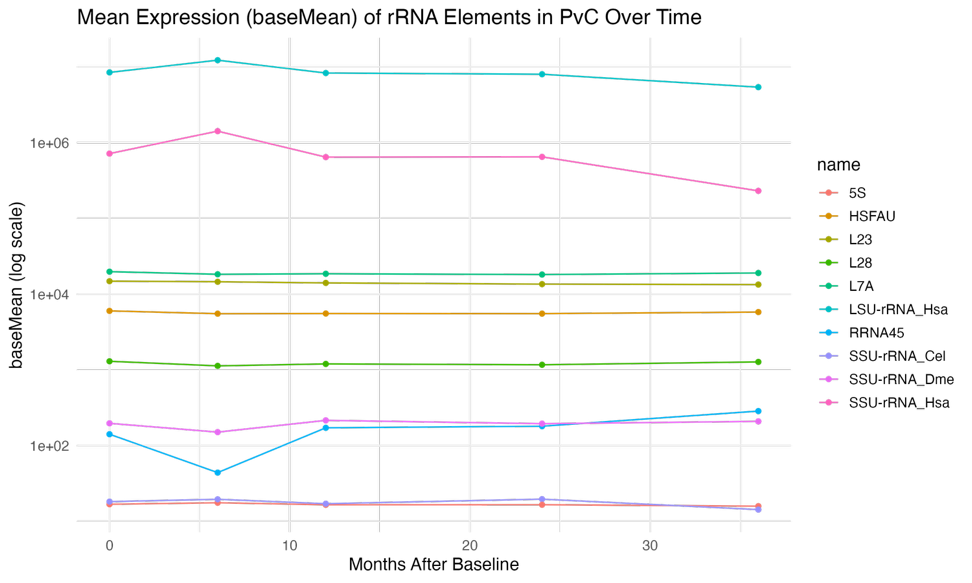

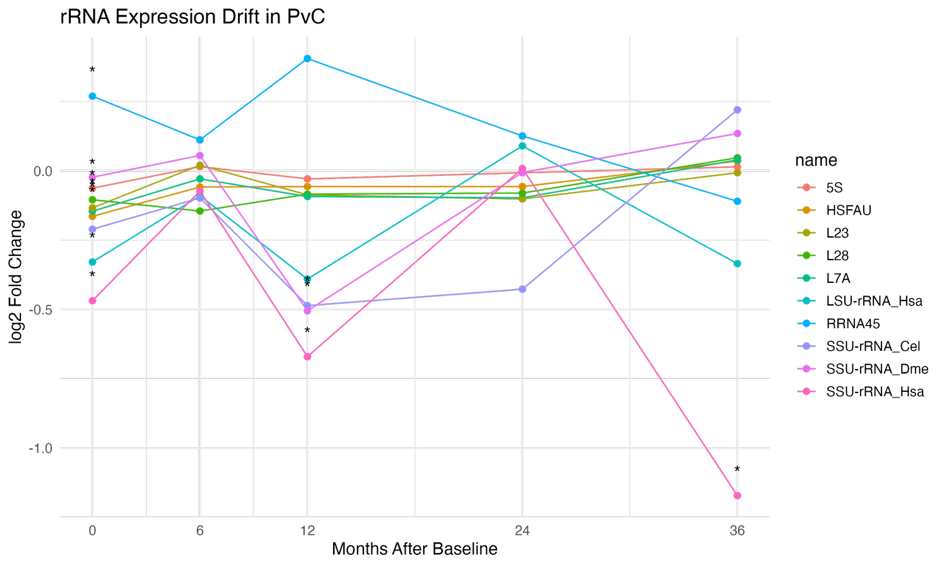


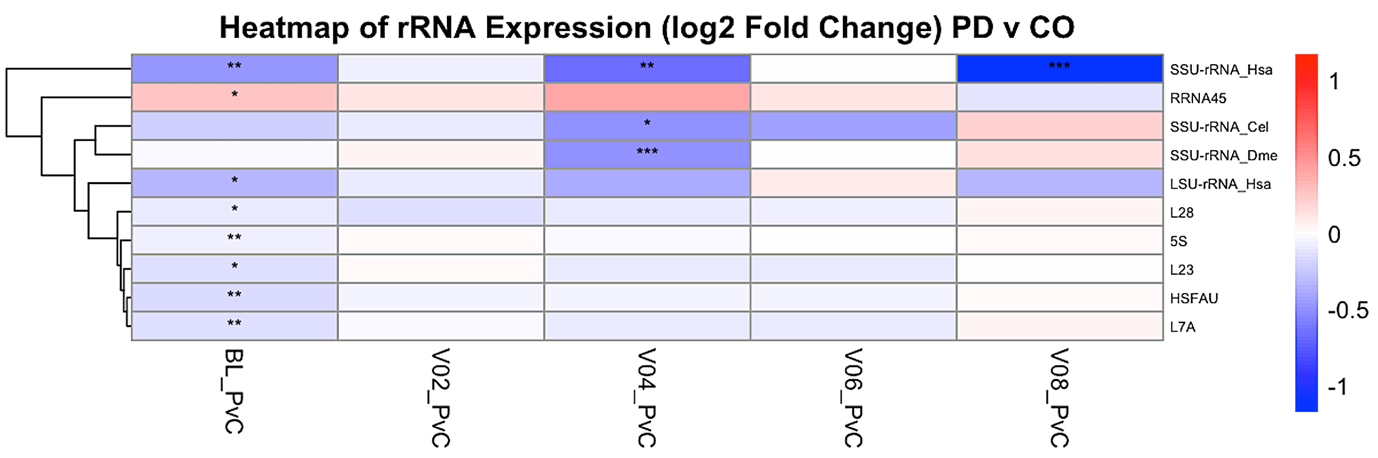


**Figure S1p.**
Differential expression of 10 rRNA Pseudogenes in peripheral blood cells of Parkinson’s disease (PD) patients versus healthy controls across five timepoints: baseline (BL), 6 months (V02), 12 months (V04), 24 months (V06), and 36 months (V08).
Top: Line plot showing the average expression (baseMean, log₁₀ scale) of the 10 rRNA across timepoints (x-axis: months after BL).
Middle: Line plot showing log₂ fold change (y-axis) in rRNA expression relative to baseline over the same timepoints.
Bottom: Heatmap of log₂ fold change in expression for each rRNA at each timepoint.
Asterisks indicate statistical significance: padj < 0.05 (*), padj < 0.01 (**), padj < 0.001 (***).

**S1q**


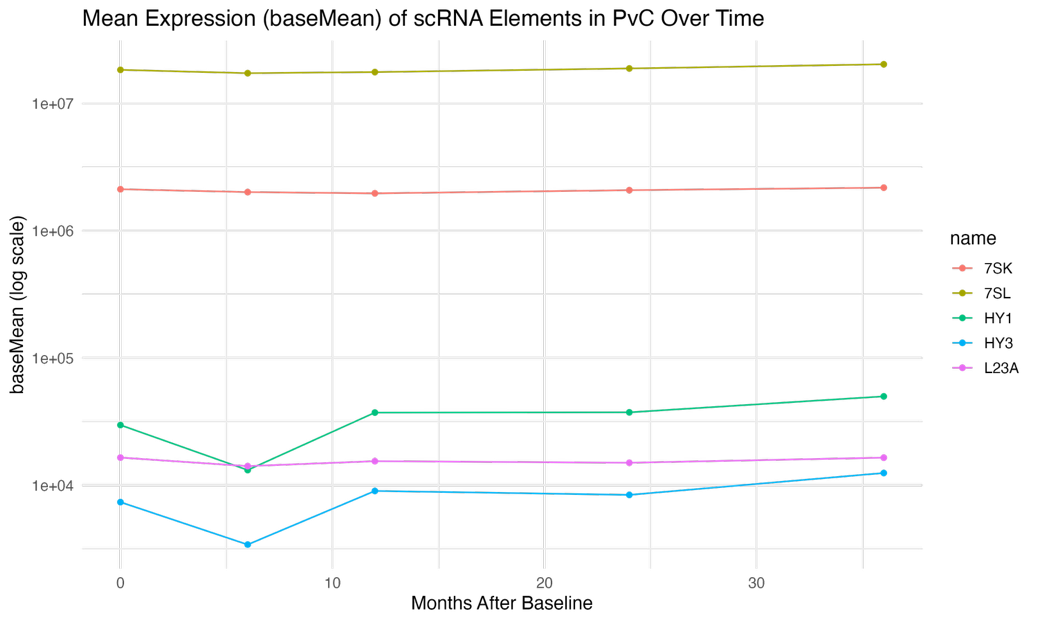


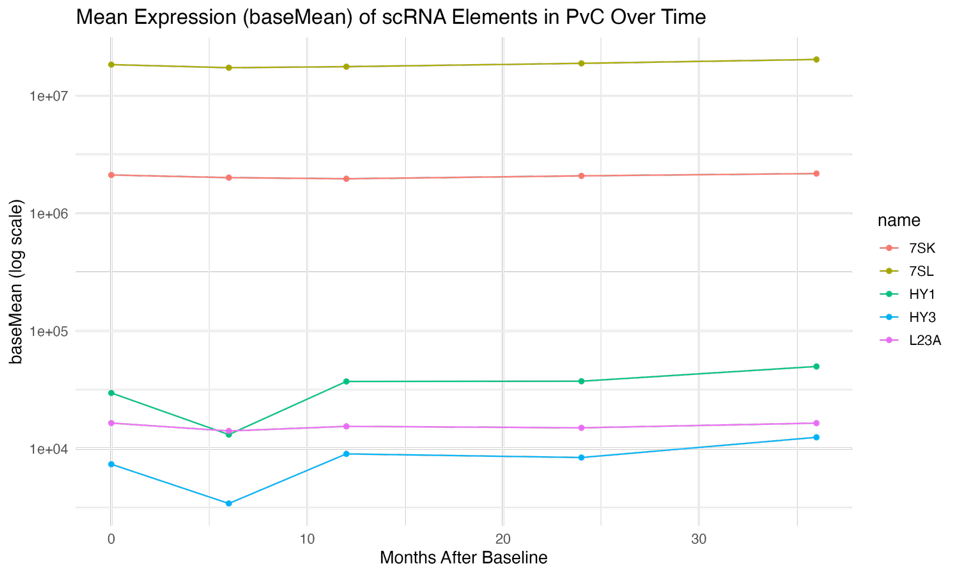


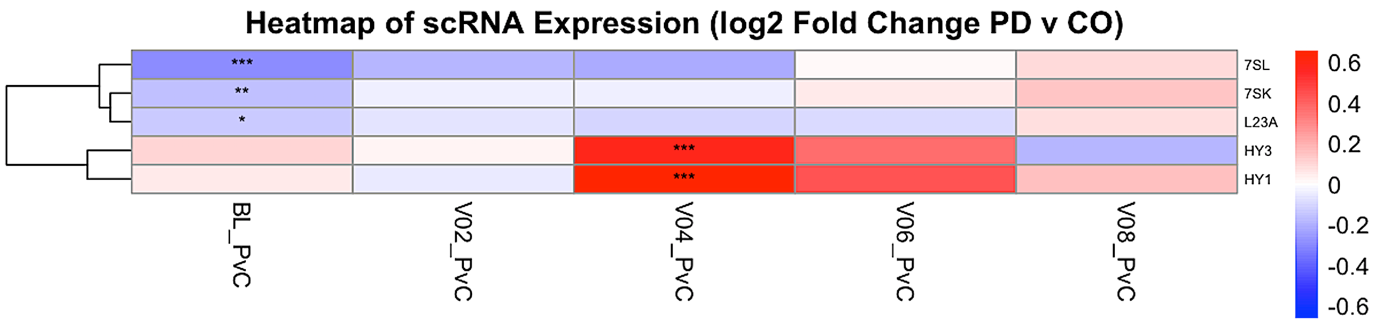


**Figure S1q.**
Differential expression of five scRNA Pseudogenes in peripheral blood cells of Parkinson’s disease (PD) patients versus healthy controls across five timepoints: baseline (BL), 6 months (V02), 12 months (V04), 24 months (V06), and 36 months (V08).
Top: Line plot showing the average expression (baseMean, log₁₀ scale) of the five scRNA across timepoints (x-axis: months after BL).
Middle: Line plot showing log₂ fold change (y-axis) in scRNA expression relative to baseline over the same timepoints.
Bottom: Heatmap of log₂ fold change in expression for each scRNA at each timepoint.
Asterisks indicate statistical significance: padj < 0.05 (*), padj < 0.01 (**), padj < 0.001 (***).

**1Sr**


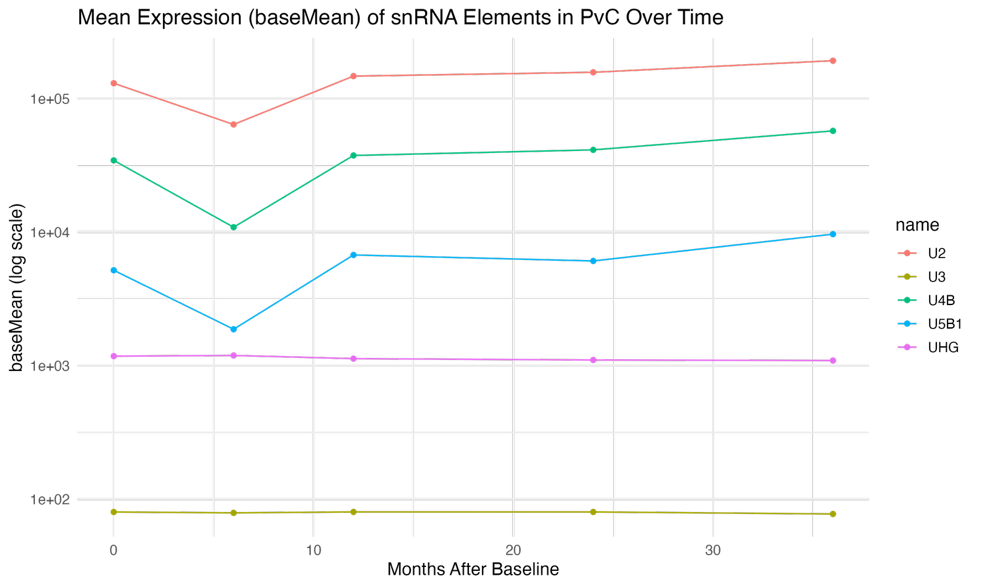


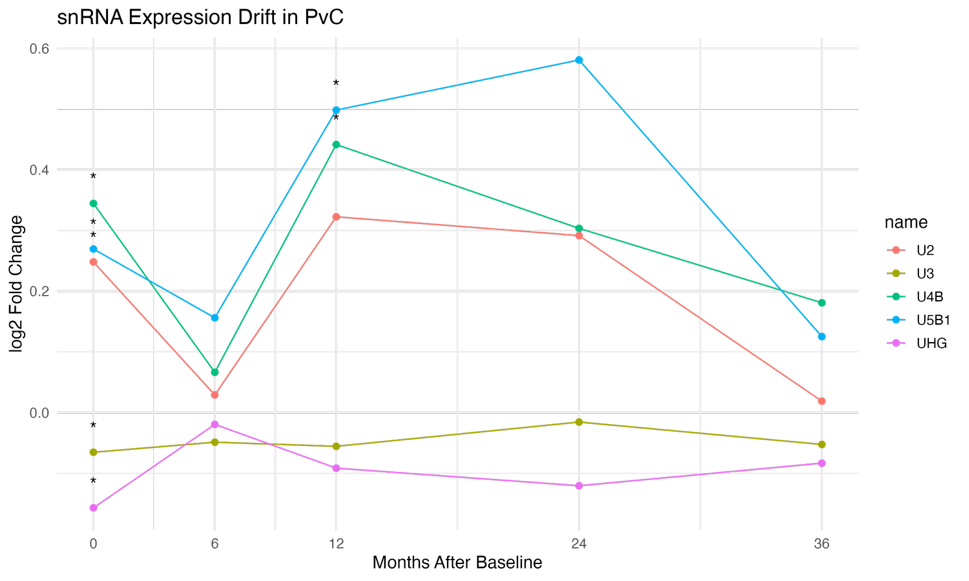


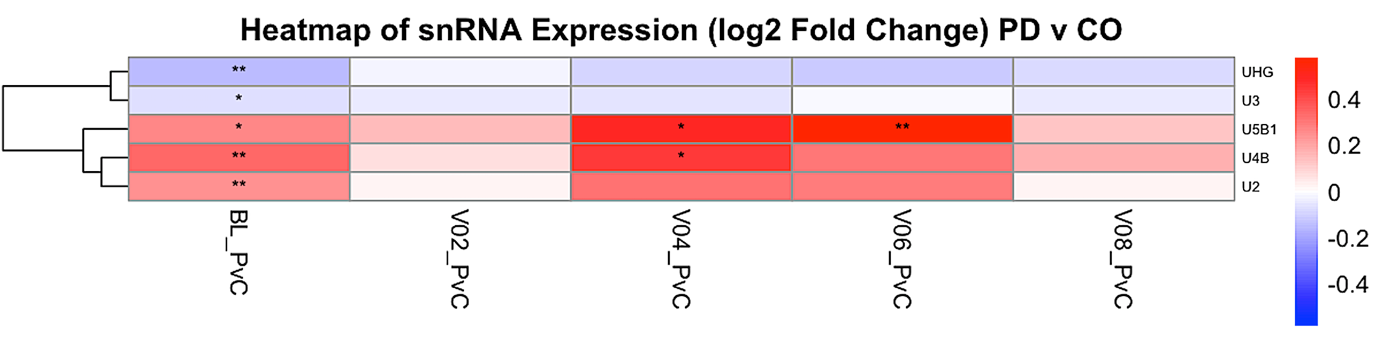


**Figure S1r.**
Differential expression of five snRNA Pseudogenes in peripheral blood cells of Parkinson’s disease (PD) patients versus healthy controls across five timepoints: baseline (BL), 6 months (V02), 12 months (V04), 24 months (V06), and 36 months (V08).
Top: Line plot showing the average expression (baseMean, log₁₀ scale) of the five snRNA across timepoints (x-axis: months after BL).
Middle: Line plot showing log₂ fold change (y-axis) in snRNA expression relative to baseline over the same timepoints.
Bottom: Heatmap of log₂ fold change in expression for each snRNA at each timepoint.
Asterisks indicate statistical significance: padj < 0.05 (*), padj < 0.01 (**).

**S1s**


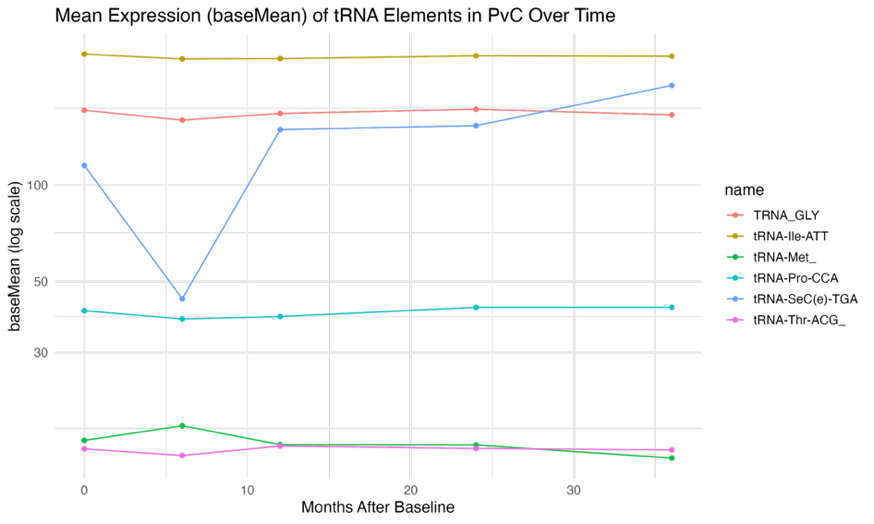


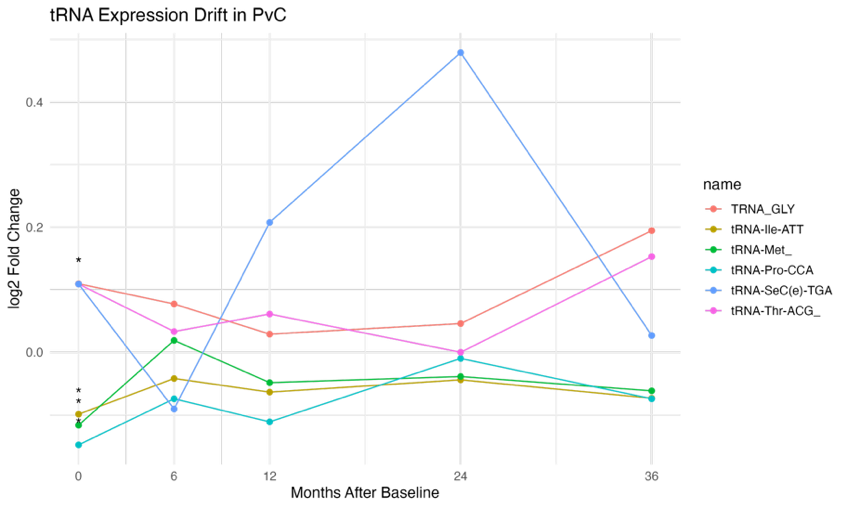


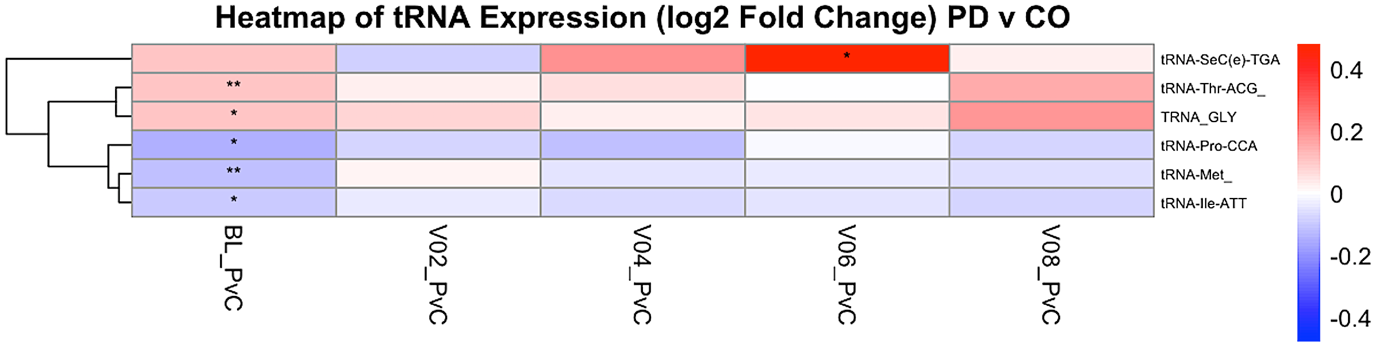


**Figure 1s.**
Differential expression of six tRNA Pseudogenes in peripheral blood cells of Parkinson’s disease (PD) patients versus healthy controls across five timepoints: baseline (BL), 6 months (V02), 12 months (V04), 24 months (V06), and 36 months (V08).
Top: Line plot showing the average expression (baseMean, log₁₀ scale) of the six tRNA across timepoints (x-axis: months after BL).
Middle: Line plot showing log₂ fold change (y-axis) in tRNA expression relative to baseline over the same timepoints.
Bottom: Heatmap of log₂ fold change in expression for each tRNA at each timepoint.
Asterisks indicate statistical significance: padj < 0.05 (*), padj < 0.01 (**).

**Fig. S2.**

1. **PD v BL. Repeat Class Expression Across Four Post-Baseline Timepoints, V02, V04, V06, V08.**


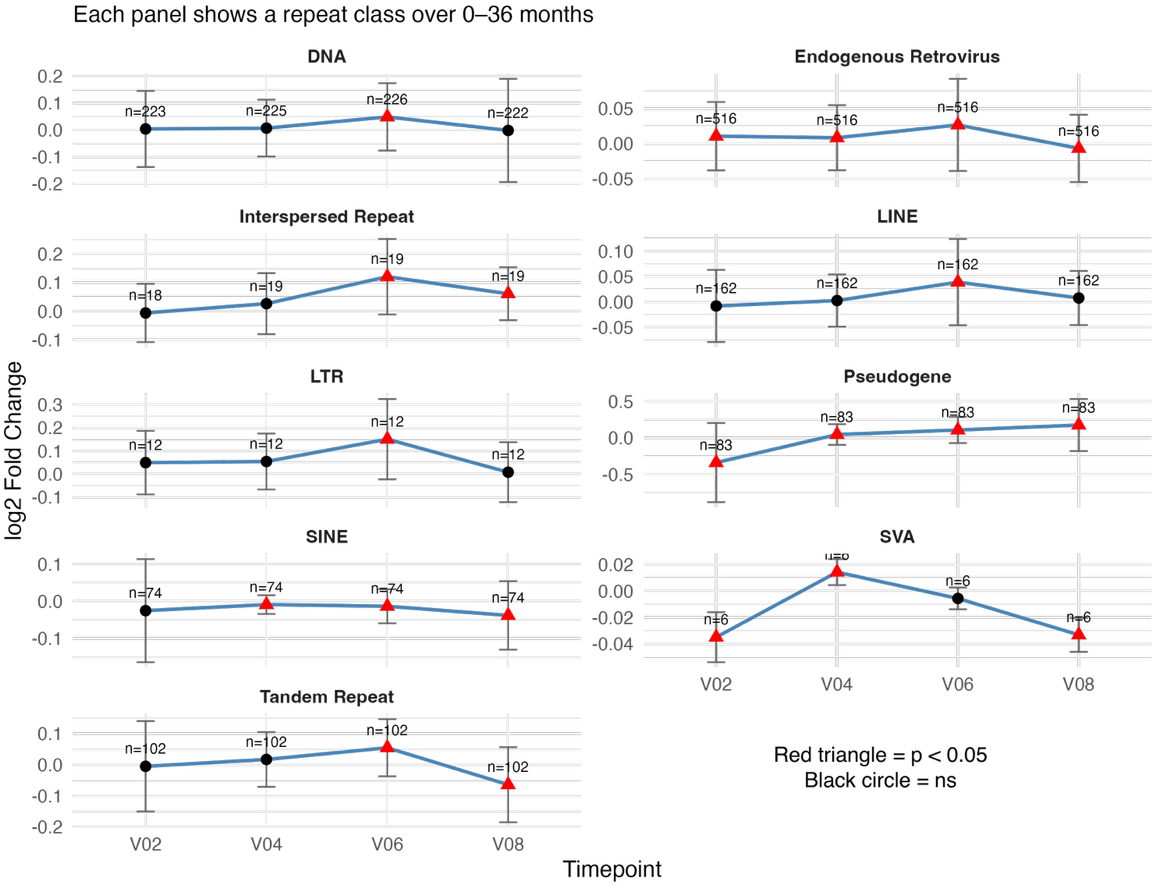


1. **CO v BL. Repeat Class Expression Across Four Post-Baseline Timepoints, V02, V04, V06, V08**.

**
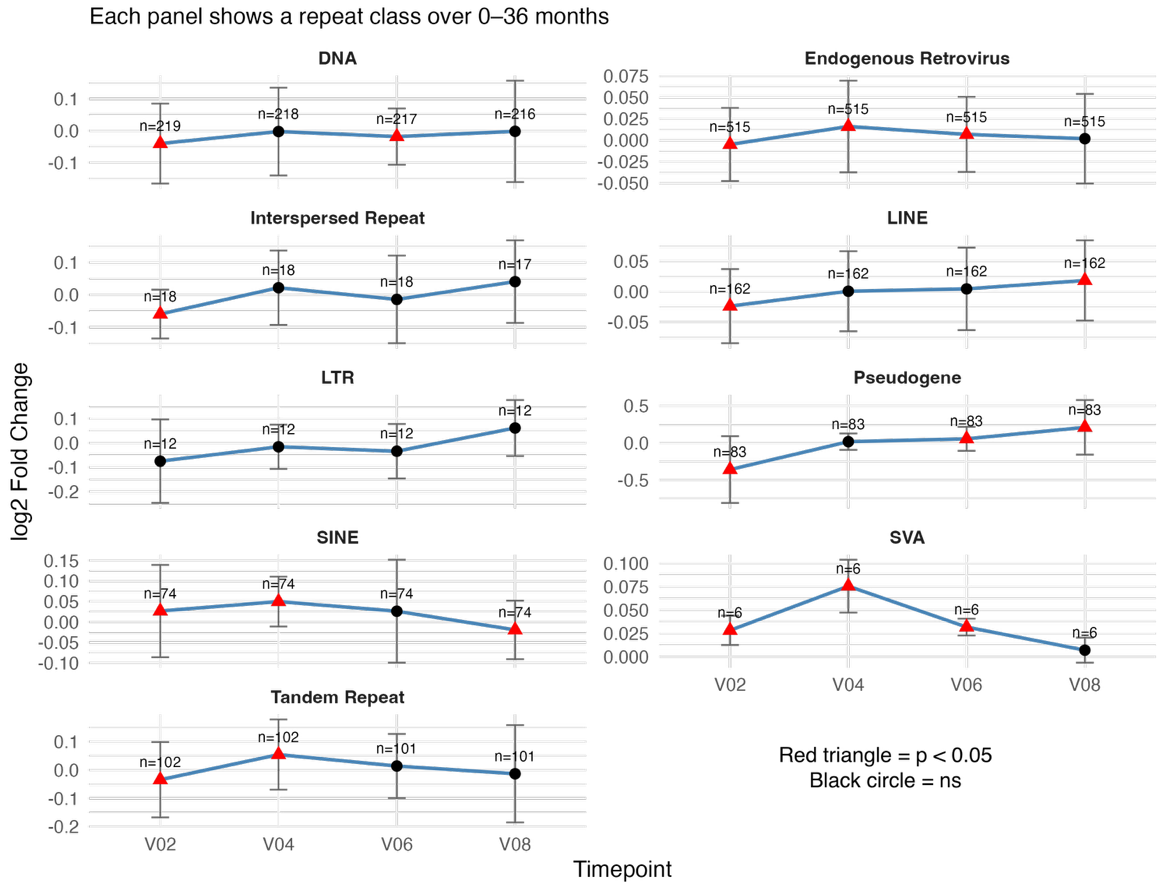
**

**Figure S2.** Temporal expression dynamics of repeatome classes in peripheral blood cells of PD v BL (a) and CO v BL (b) groups. Each panel represents a distinct transposable element (TE) class (e.g., DNA transposons, ERV & LTR retrotransposons, LINEs, SINEs, SVAs, pseudogenes, and tandem/interspersed repeats). Points indicate the mean log₂ fold change in expression at each time point (V02-6 months, V04-12 months, V06-24 months, and V08-36 months), with vertical bars showing the standard deviation (SD). Red triangles denote statistically significant differential expression (p < 0.05), while black circles indicate non-significant changes. Sample size (n) is displayed above each point. These trends summarize the evolving expression landscape of major repeat classes across time.

**Figure S3a-m**. Temporal changes in expression of different repeat subfamilies in peripheral blood cells of PD v BL and CO v BL groups at four timepoints (V02, V04, V06, V08) relative to baseline (BL). Line plots show the log₂ fold change in expression of the following Repeat subfamilies: a, Alu; b, CR1; c, ERV1; d, ERV2; e, ERV3; f, hAT; g, L1; h, Mariner_Tc1; I, Other-DNA; j, Satellite; k, Simple repeats; l, SVA; and m, tRNA. Each facet represents a different Repeat subfamily member. Only elements that showed a statistically significant change (adjusted p < 0.05) at one or more timepoints are included. Data points are colored by group (PD or CO), with asterisks indicating statistical significance (padj < 0.05: *, < 0.01: **, < 0.001: ***). Line plots of log₂ fold change were generated using R ggplot2.

S3A. Alu


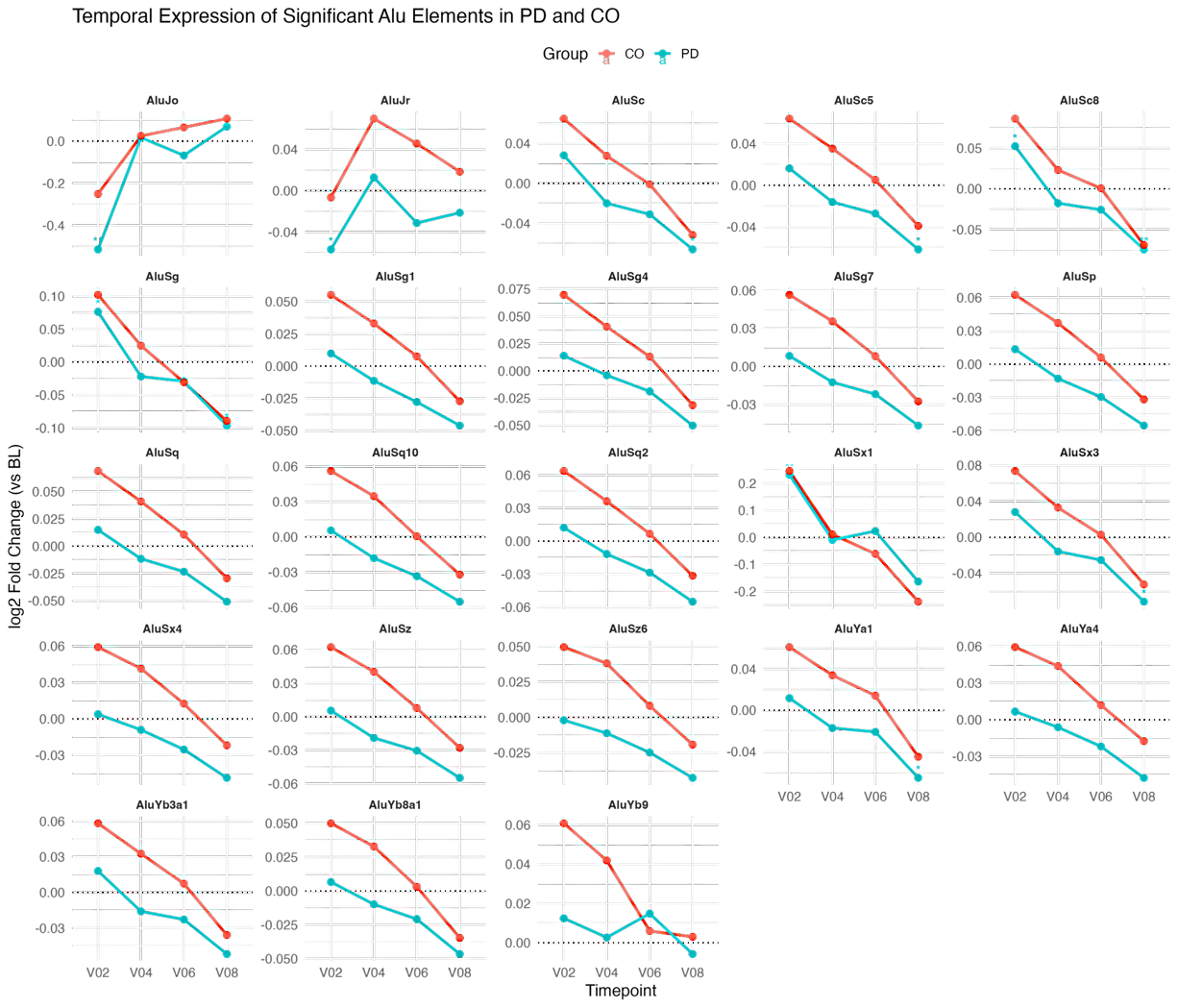


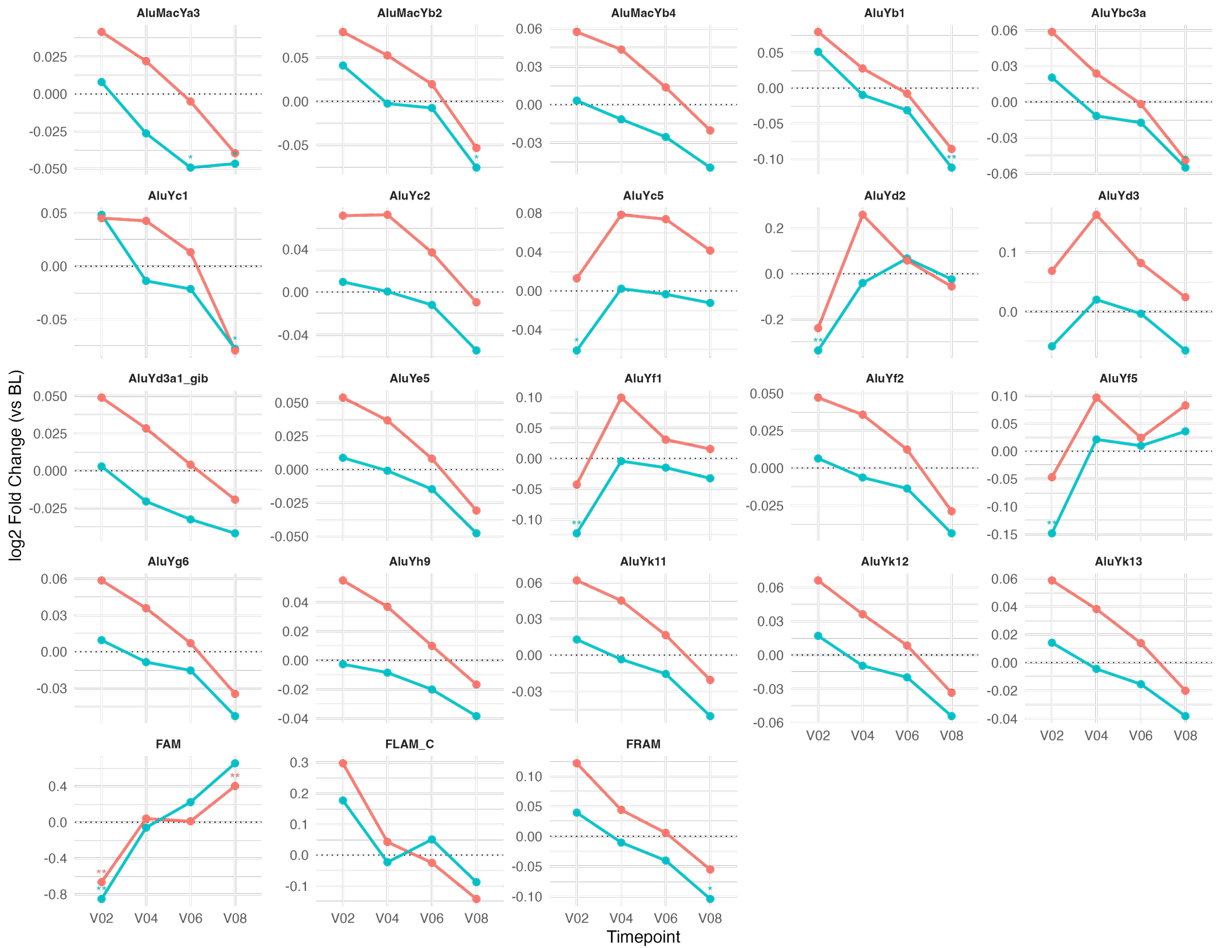


S3B. CR1


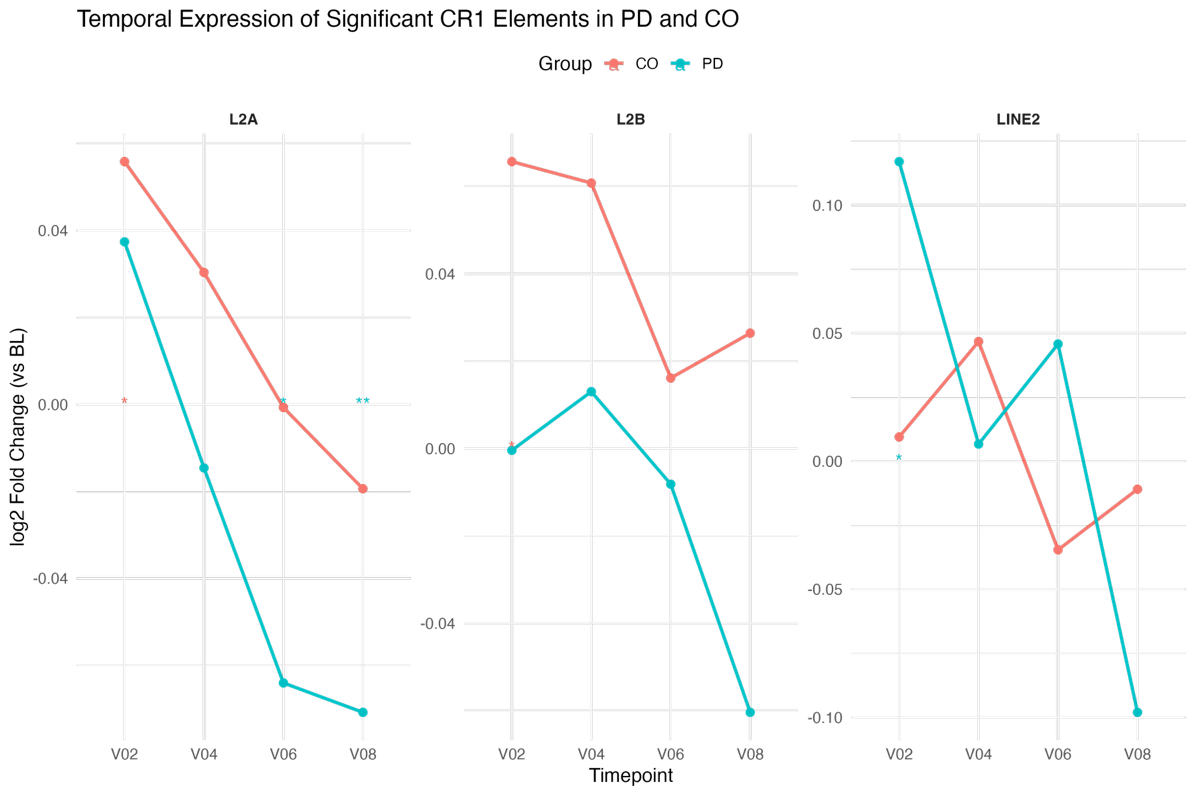


S3C. ERV1


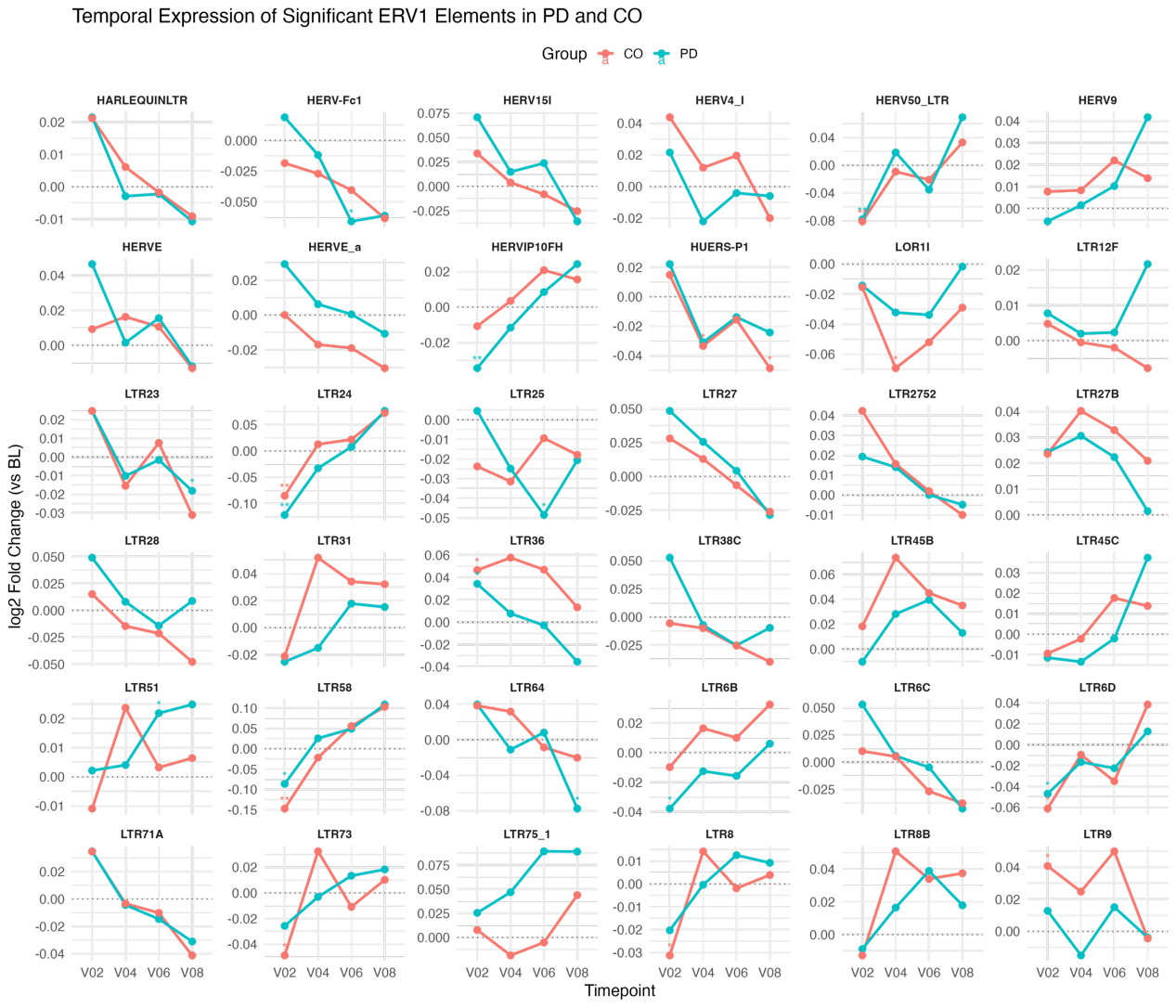


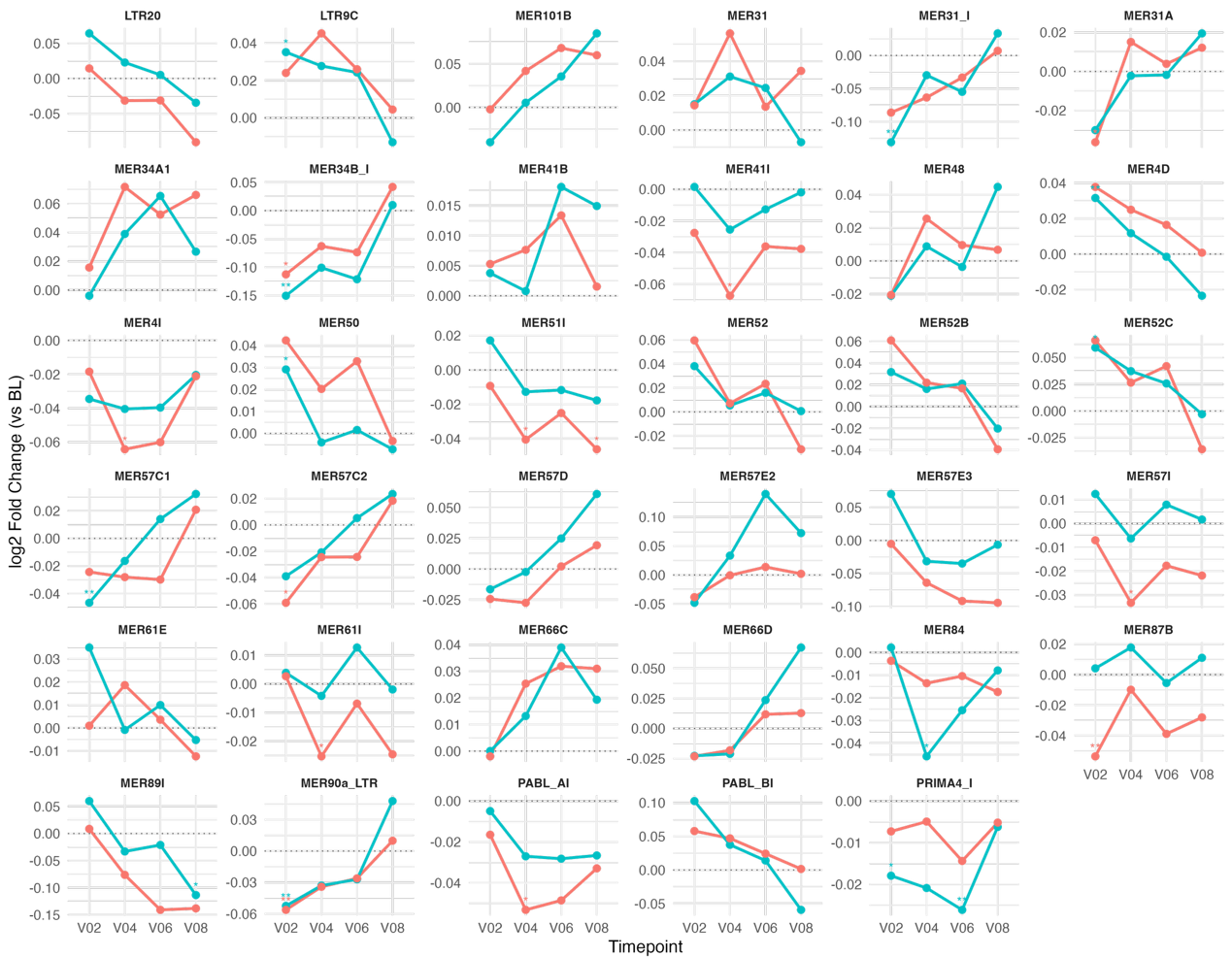


S3D. ERV2


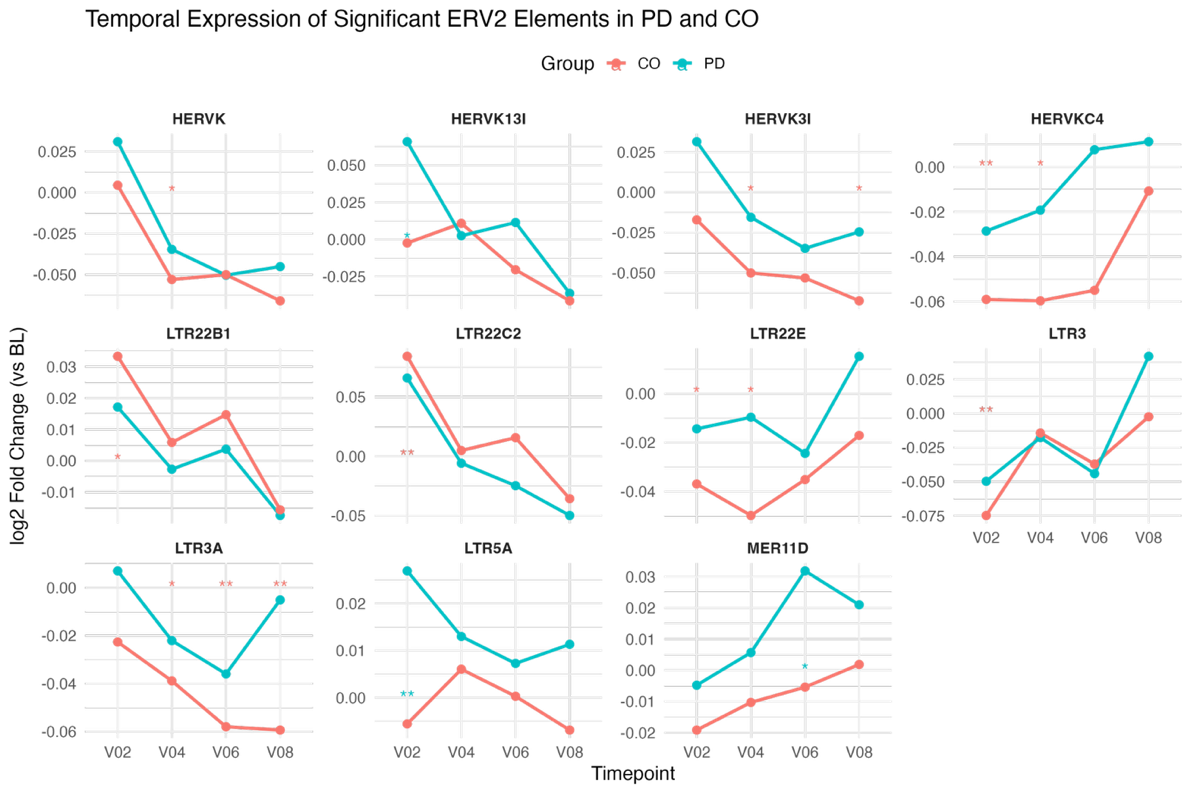


S3E. ERV3


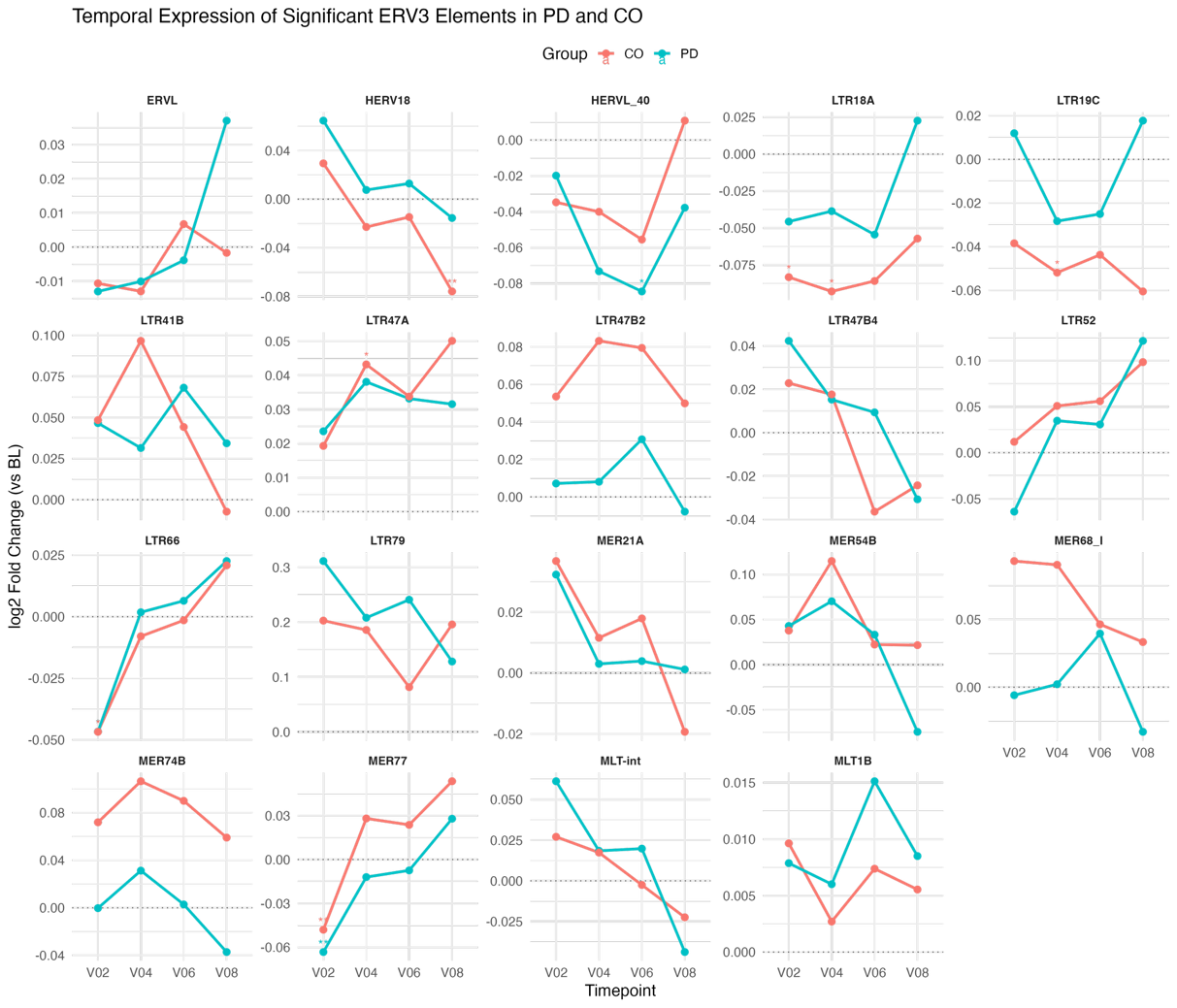


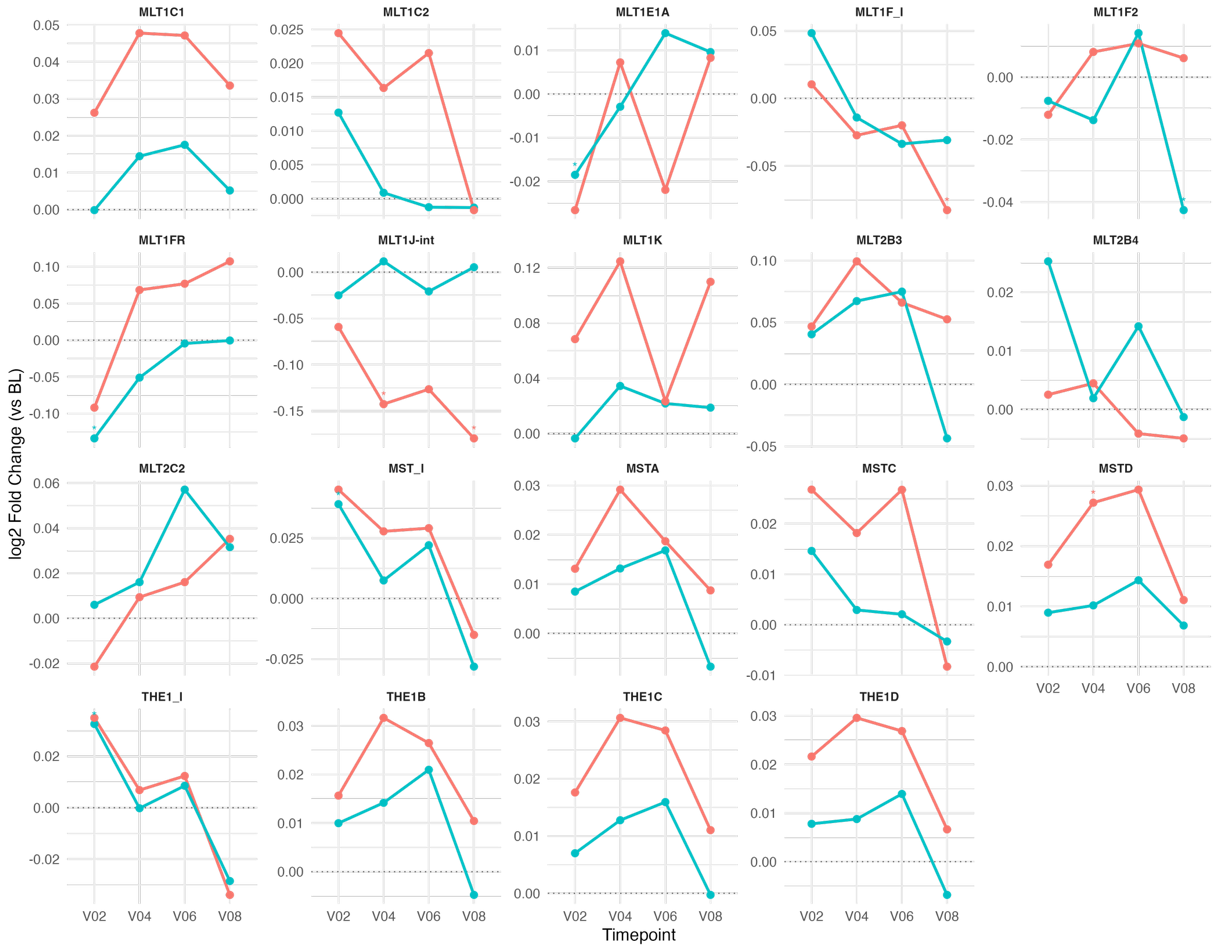


S3F. hAT


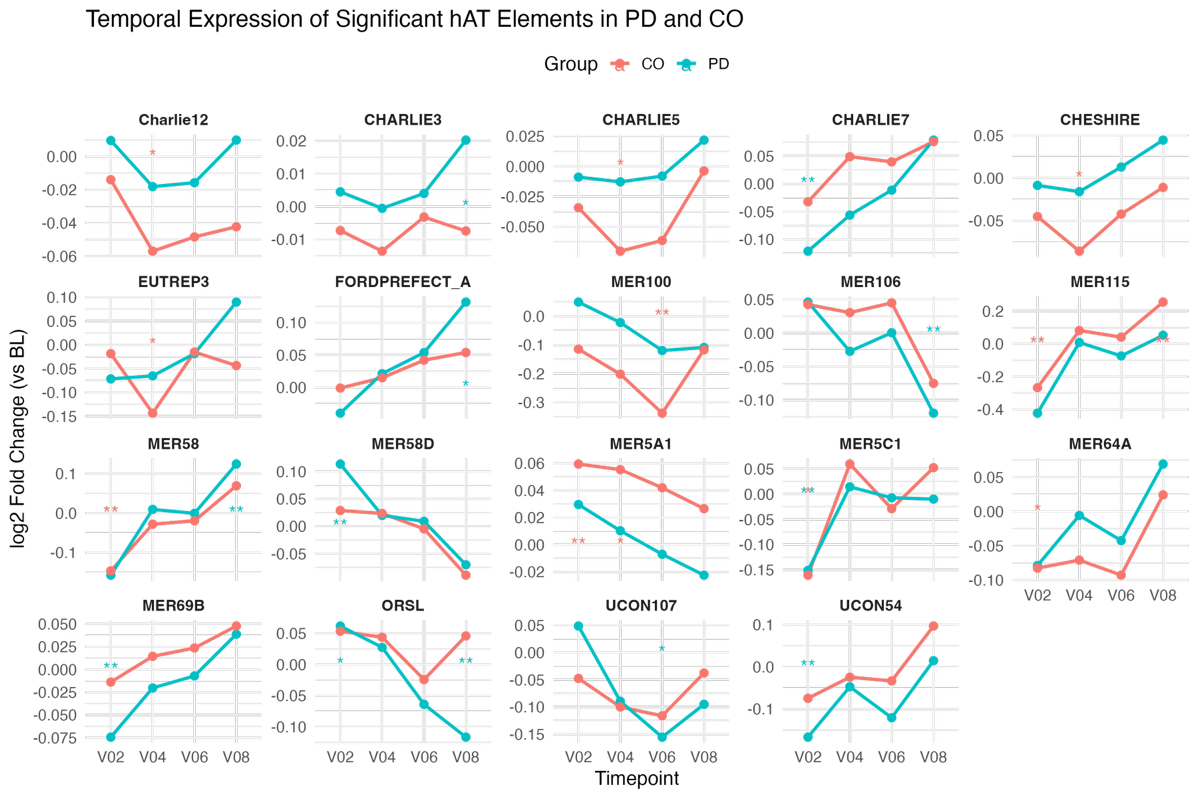


S3G. L1


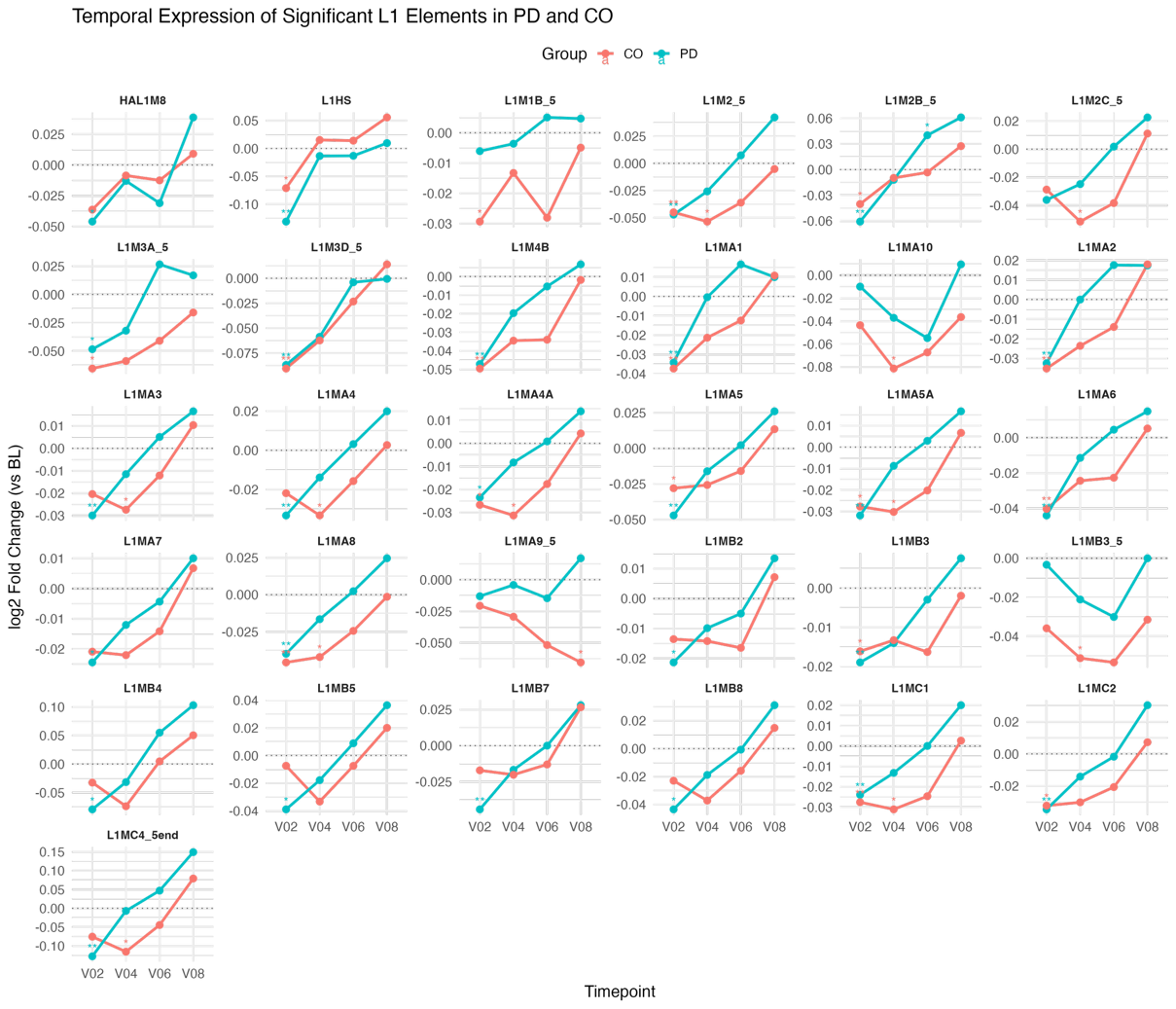


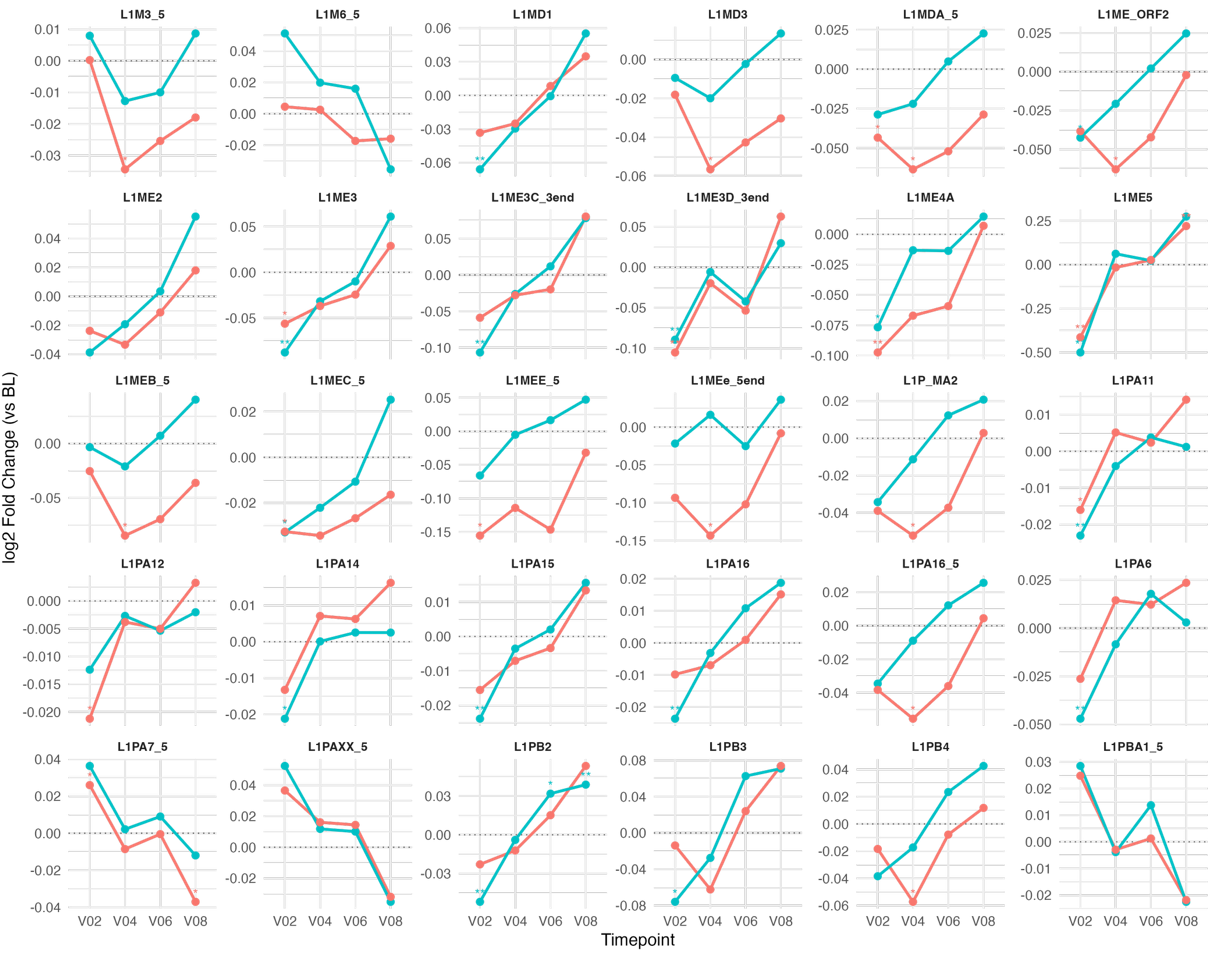


S3H. Mariner_Tc1


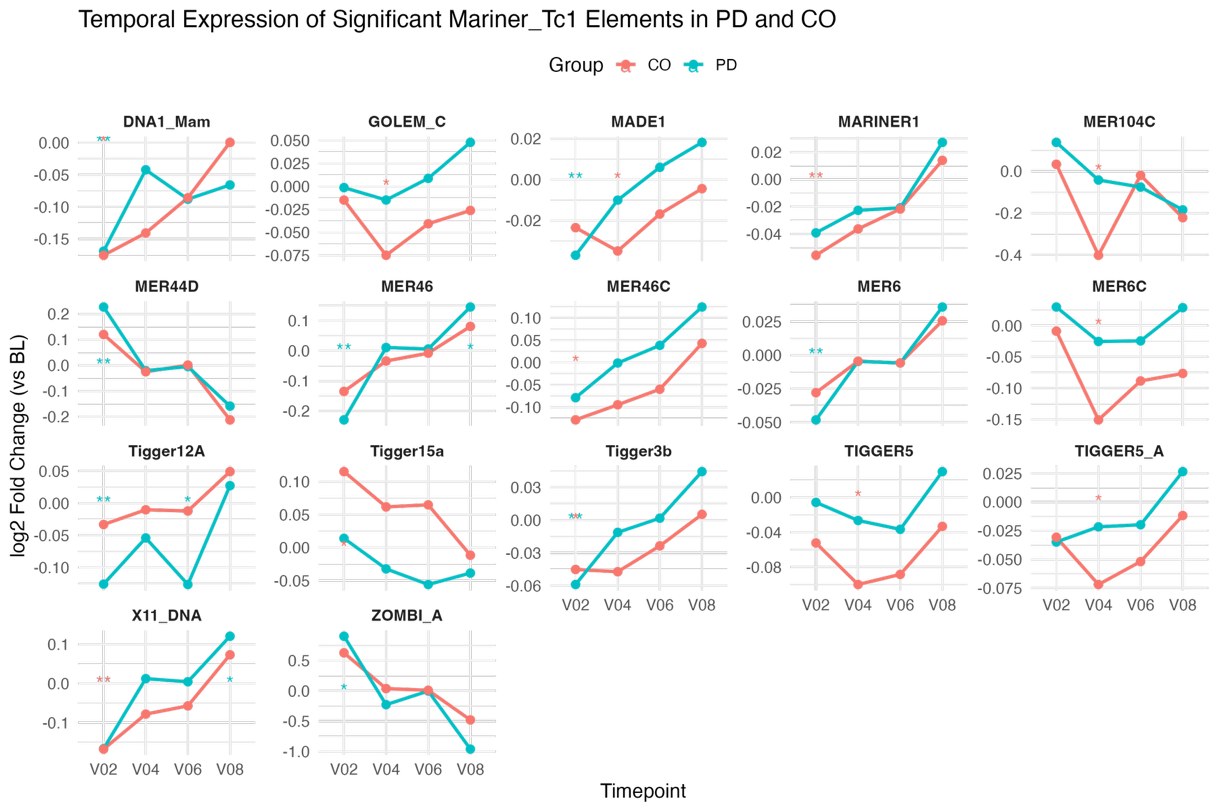


S3I. Other DNA elements


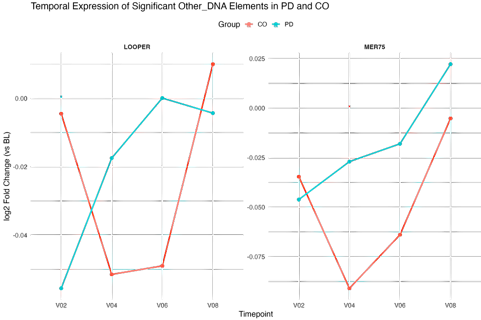


S3J. Satellite elements


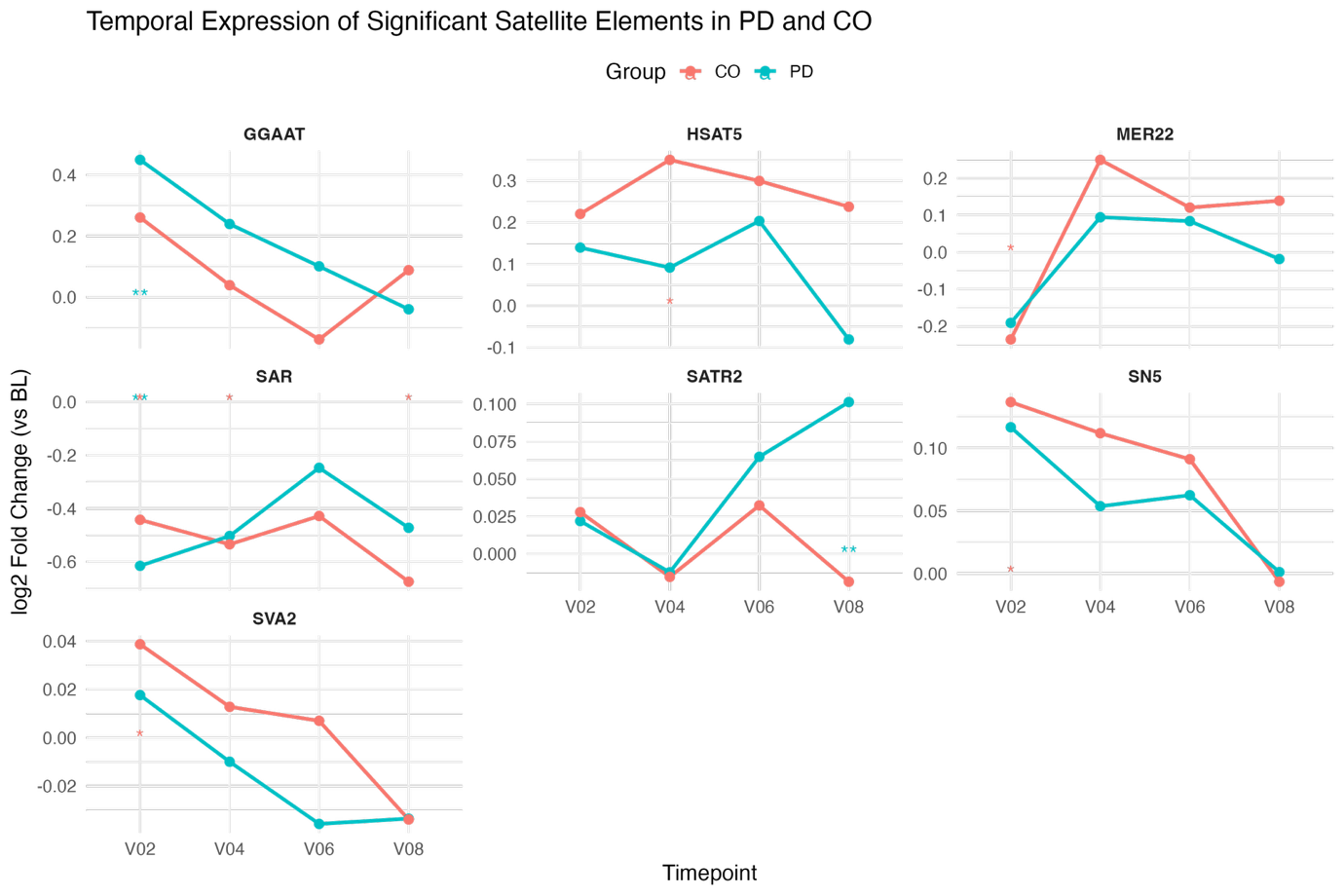


S3K. Simple Elements


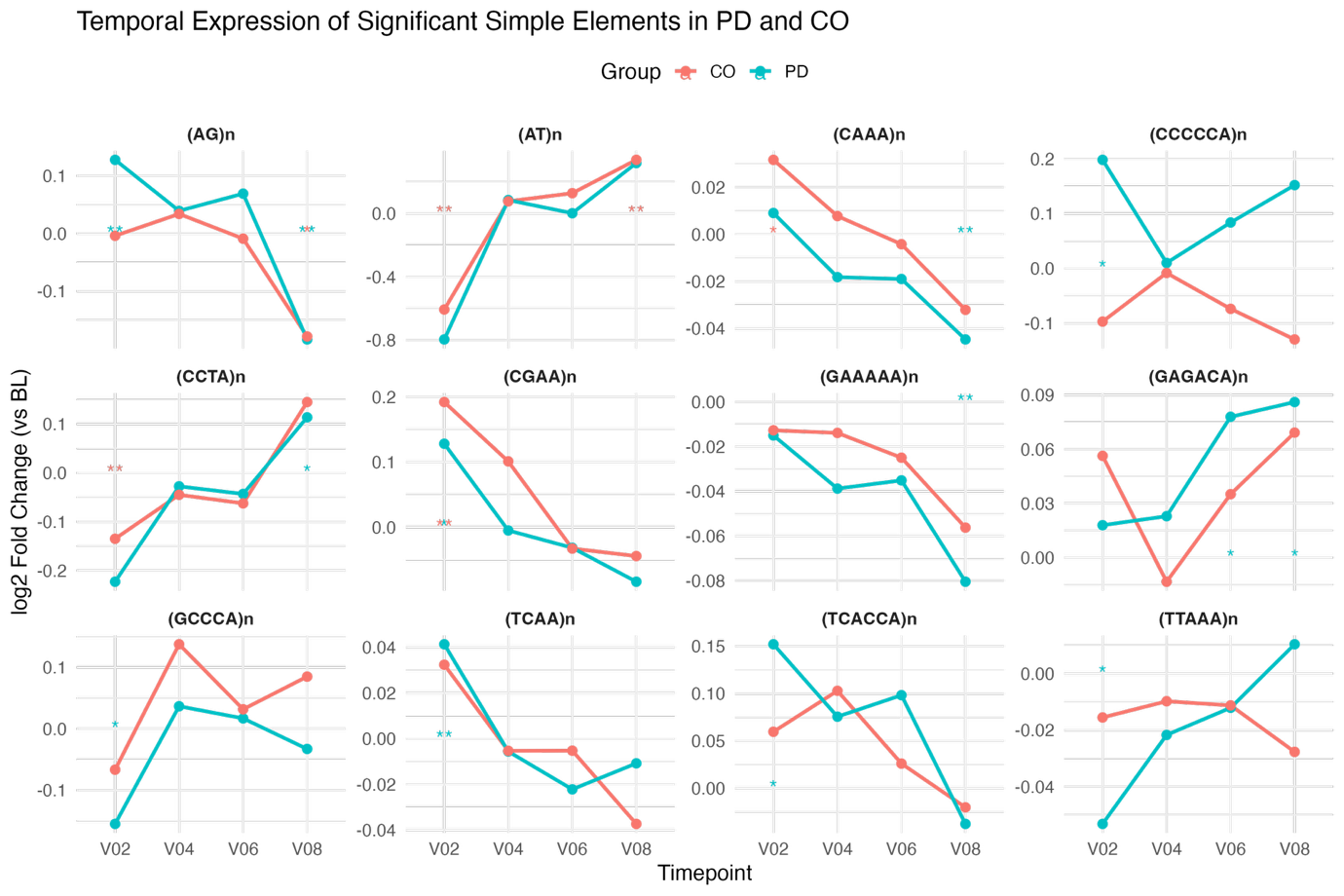


S3L. SVA


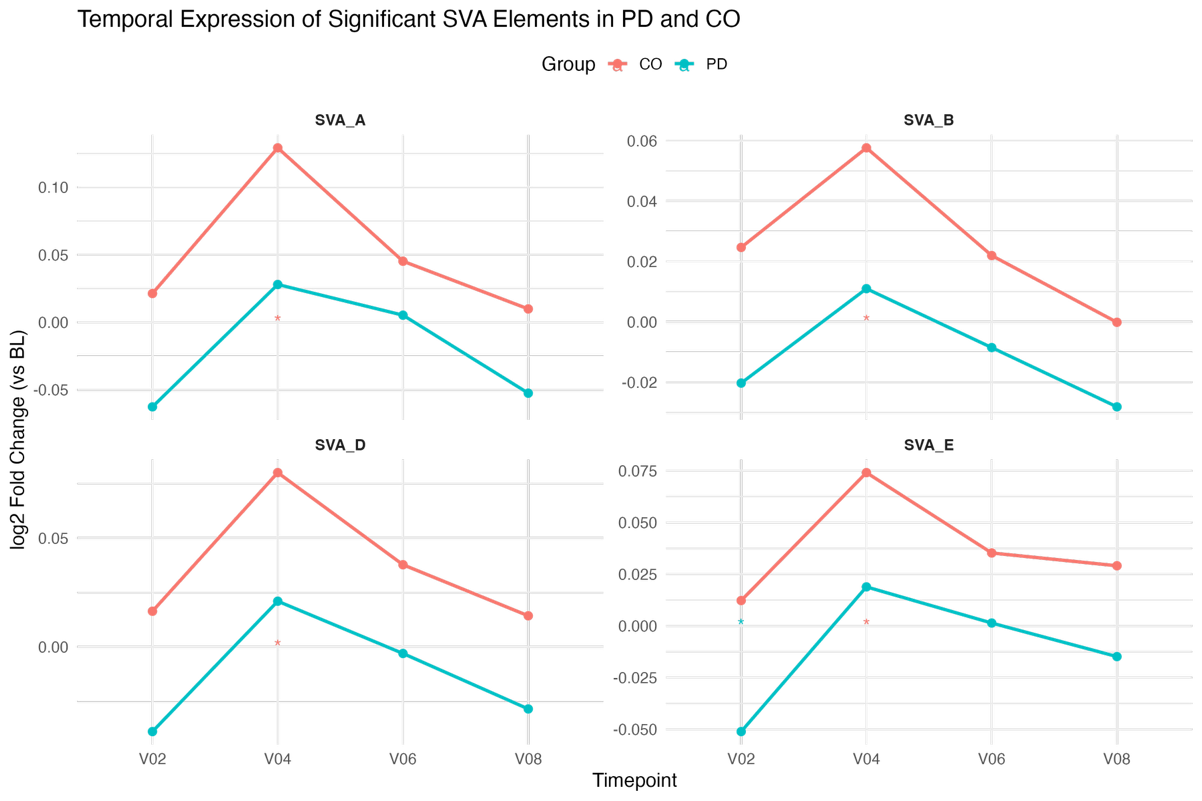


S3M. tRNA


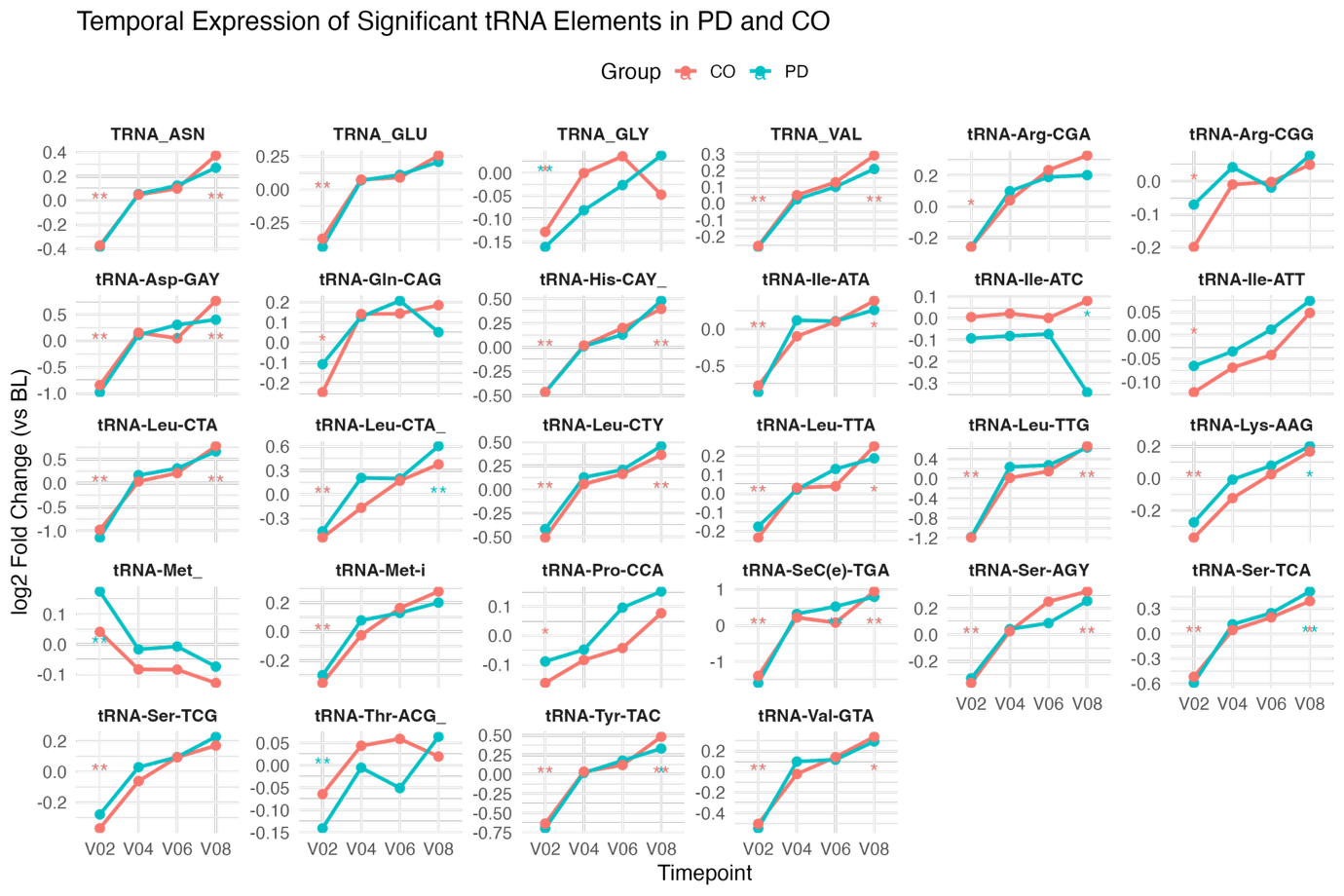


**Figure S3a-m**. Temporal changes in expression of different repeat subfamilies in peripheral blood cells of PD v BL and CO v BL groups at four timepoints (V02, V04, V06, V08) relative to baseline (BL). Line plots show the log₂ fold change in expression of the following Repeat subfamilies: a, Alu; b, CR1; c, ERV1; d, ERV2; e, ERV3; f, hAT; g, L1; h, Mariner_Tc1; I, Other-DNA; j, Satellite; k, Simple repeats; l, SVA; and m, tRNA. Each facet represents a different Repeat subfamily member. Only elements that showed a statistically significant change (adjusted p < 0.05) at one or more timepoints are included. Data points are colored by group (PD or CO), with asterisks indicating statistical significance (padj < 0.05: *, < 0.01: **, < 0.001: ***). Line plots of log₂ fold change were generated using R ggplot2.

**Supplementary Figure 4a-p**. Heatmaps for differential expression patterns of 15 different Repeat subfamilies in peripheral blood cells of PD v BL and CO v BL groups at visits V02 (6 months), V04 (12 months), V06 (24 months), and V08 (36 months): a, Alus; b, CR1s, c, ERV1s, d, ERV2s; e, ERV3s; f, hATs; g, L1s; h, Mariner_Tc1s; I, Other-DNA elements; j, rRNAs, k, Satellites; l, scRNAs; m, Simple repeats; n, snRNAs; o, SVAs; and p, tRNAs. Heatmaps show log₂ fold change in expression for each element at each timepoint with a significant change in expression at least at one visit. Asterisks indicate statistical significance: padj < 0.05 (*), padj < 0.01 (**), padj < 0.001 (***). Heatmaps were created using the pheatmap R package. See Supplementary Materials3.RScripts

S4a, Alus


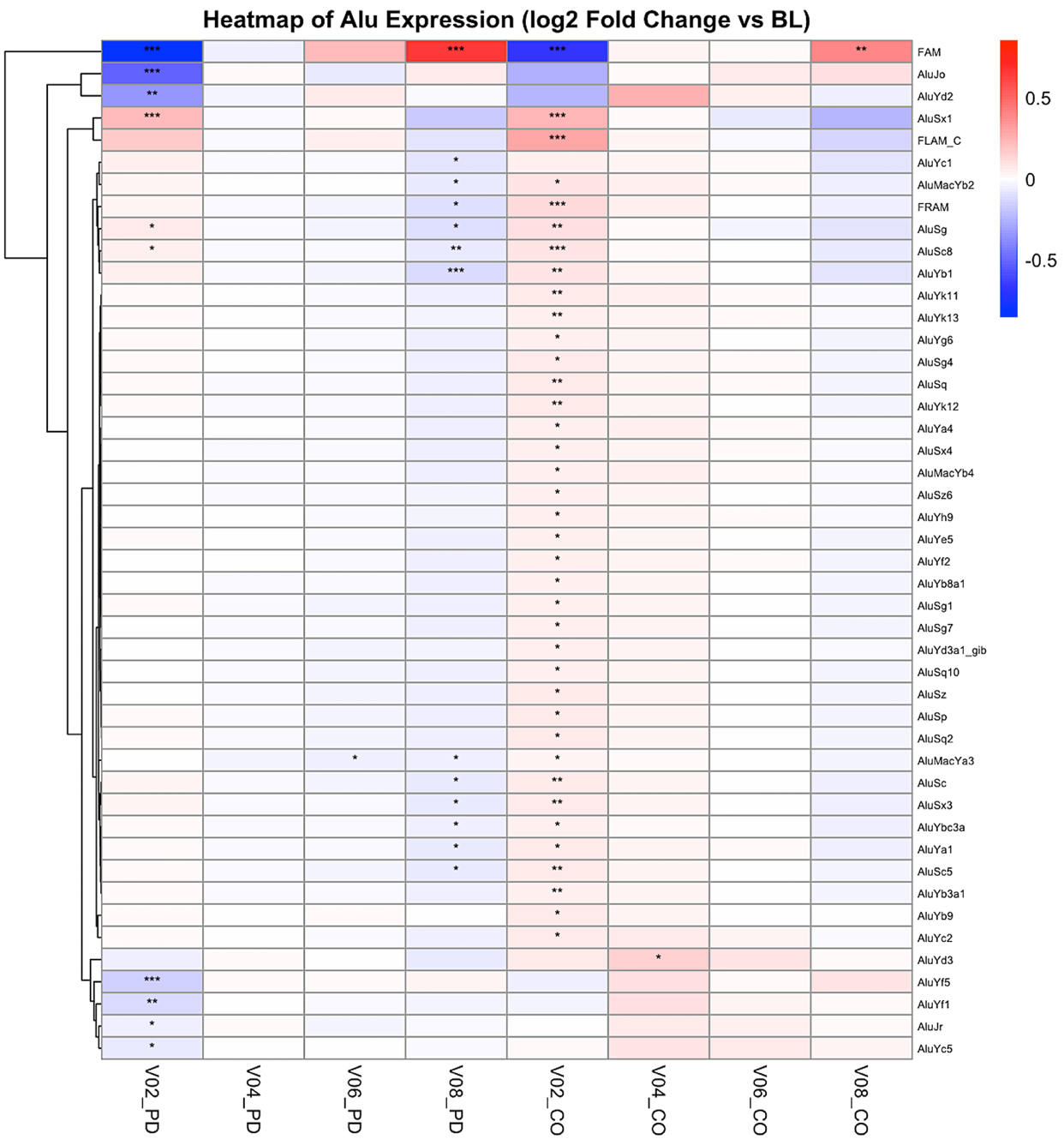


S4b, CR1s


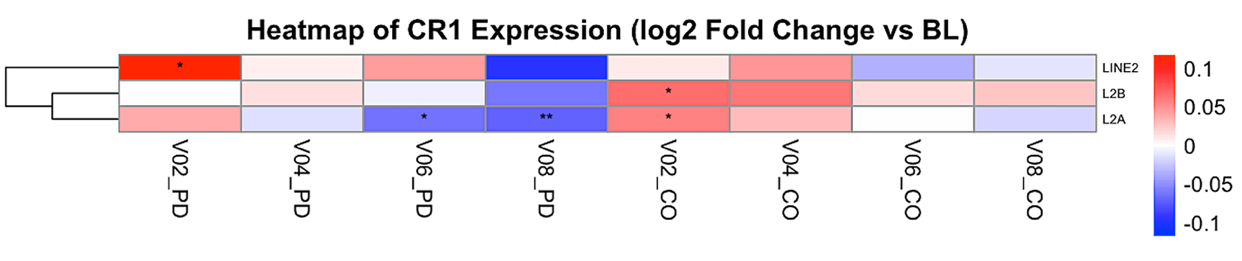


S4c, ERV1s


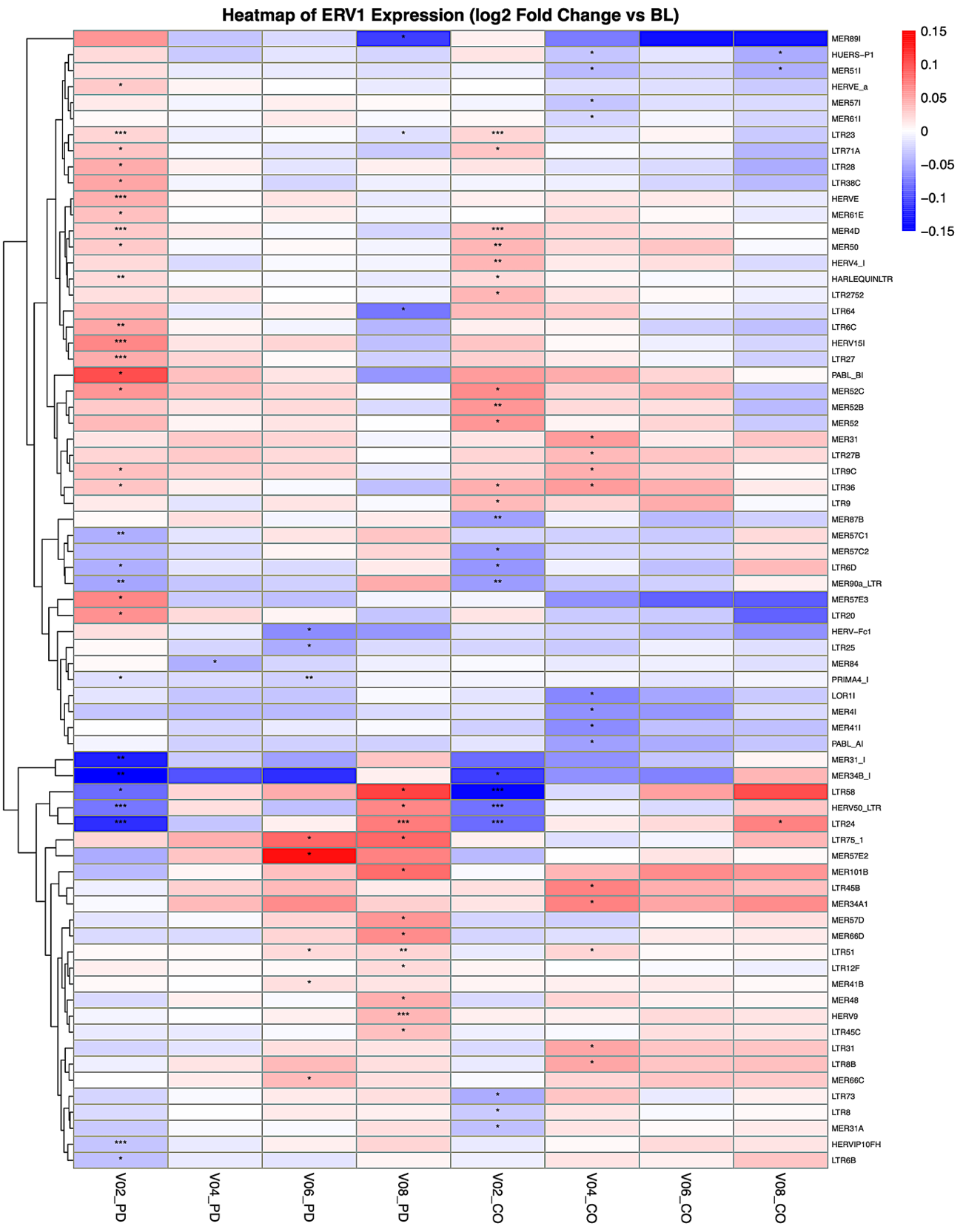


S4d, ERV2s


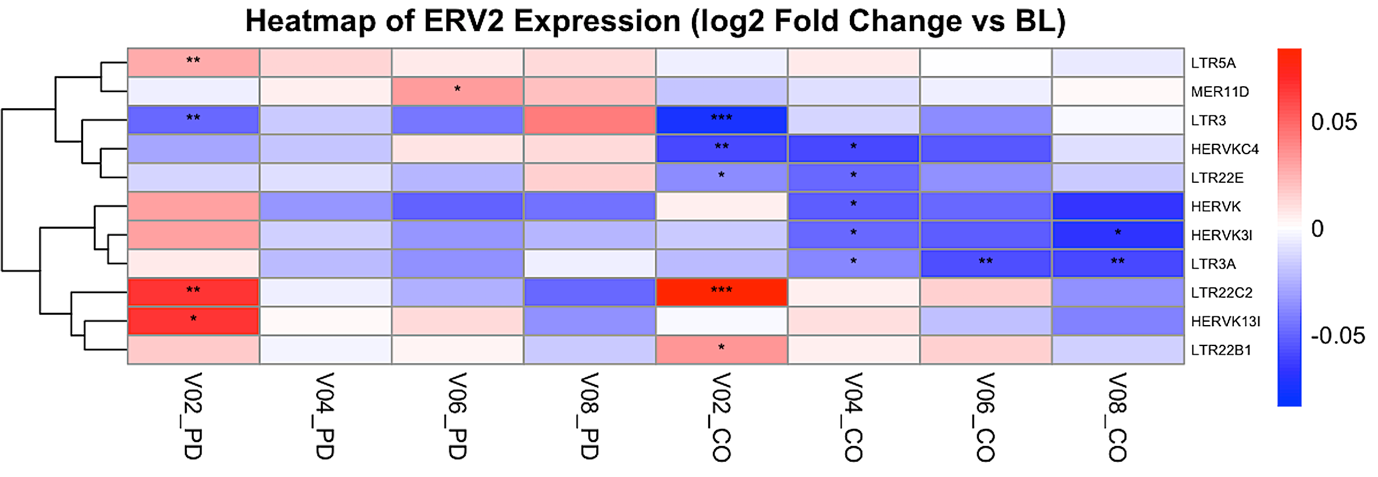


S4e, ERV3s


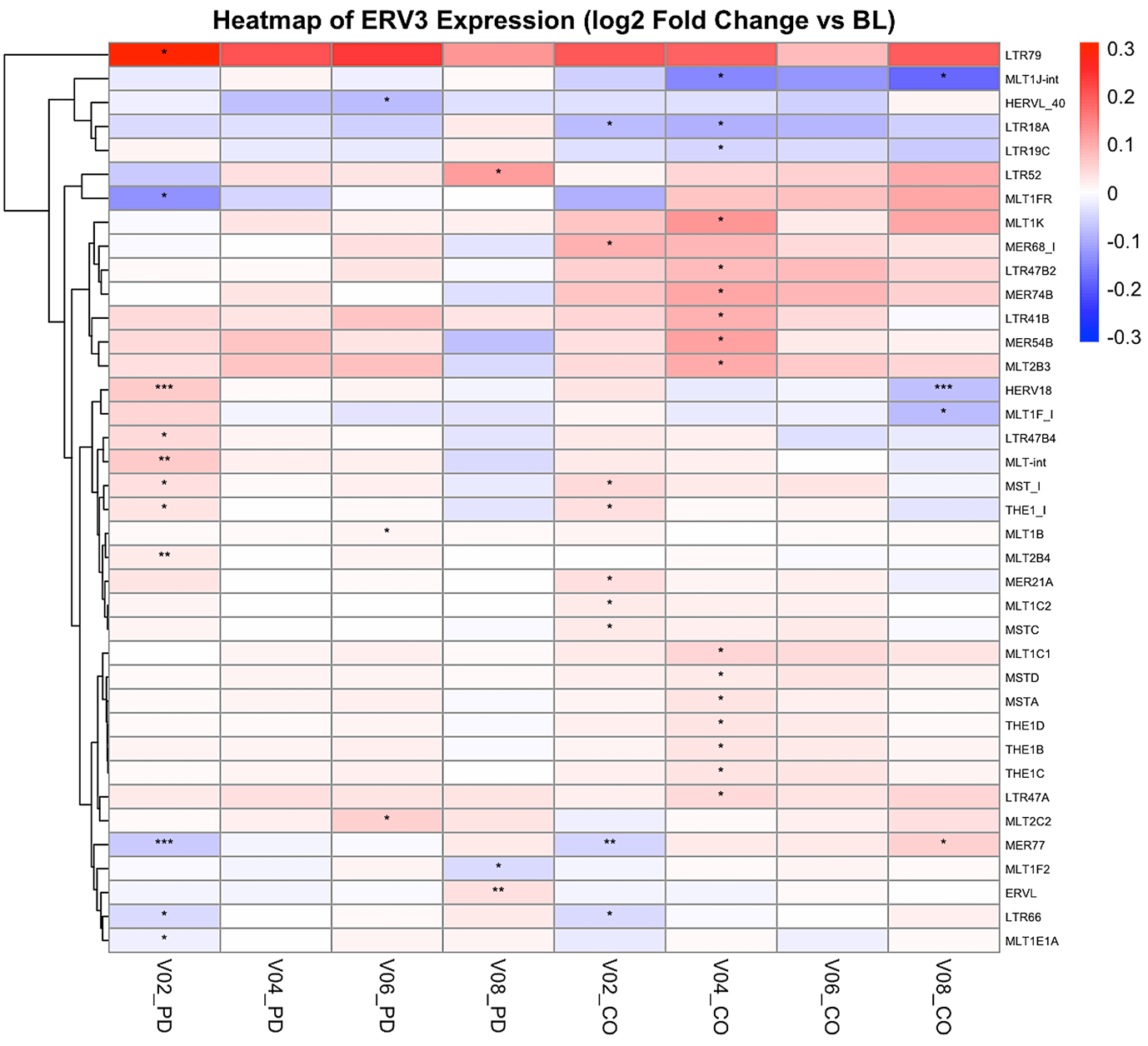


S4f, hATs


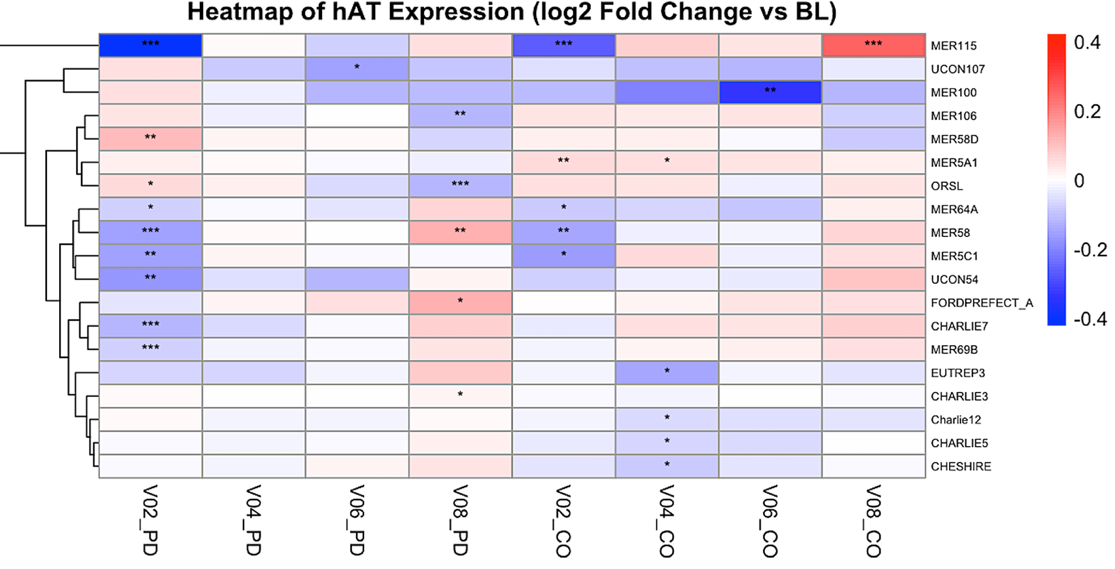


S4g, L1s


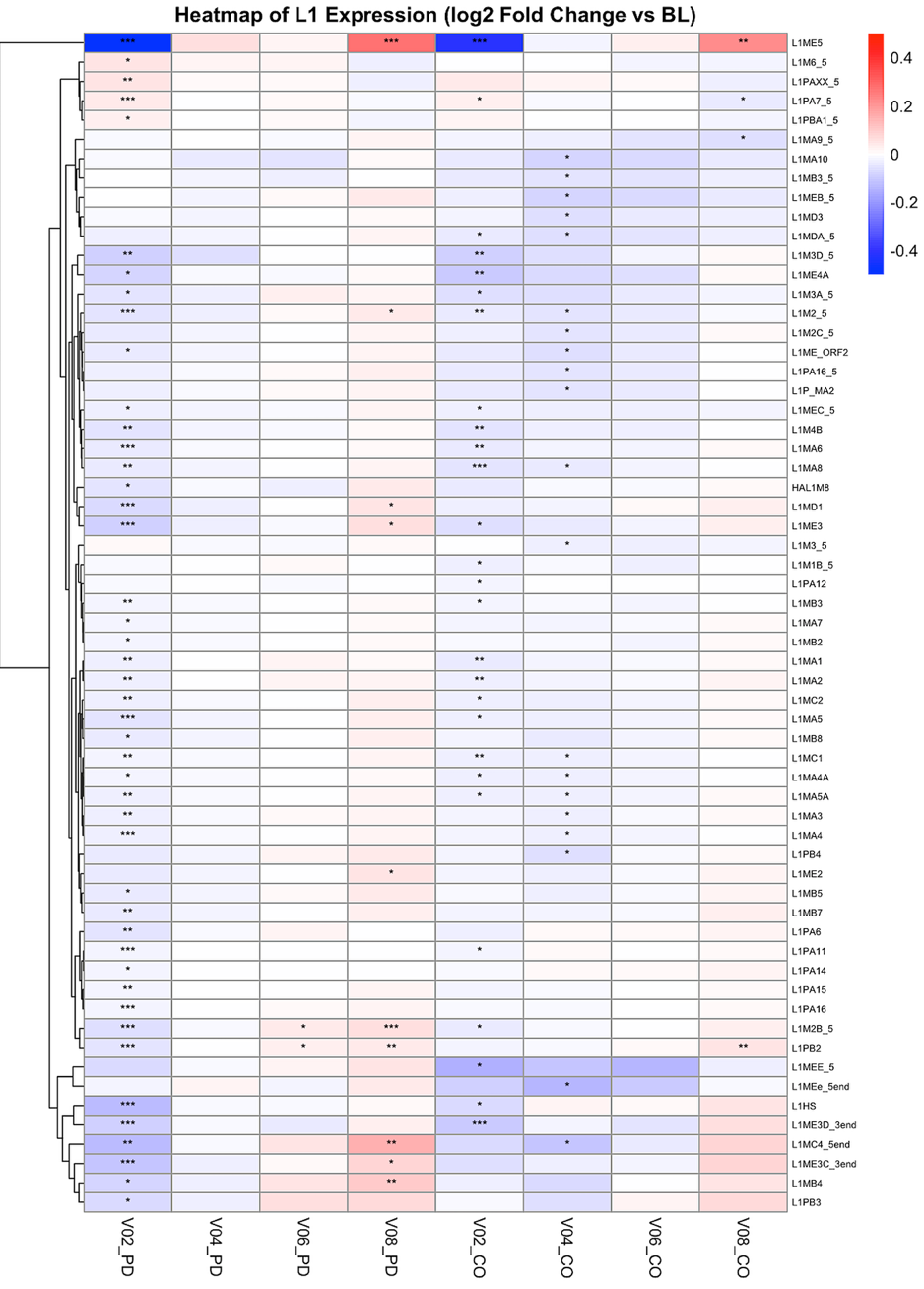


S4h, Mariner_Tc1s


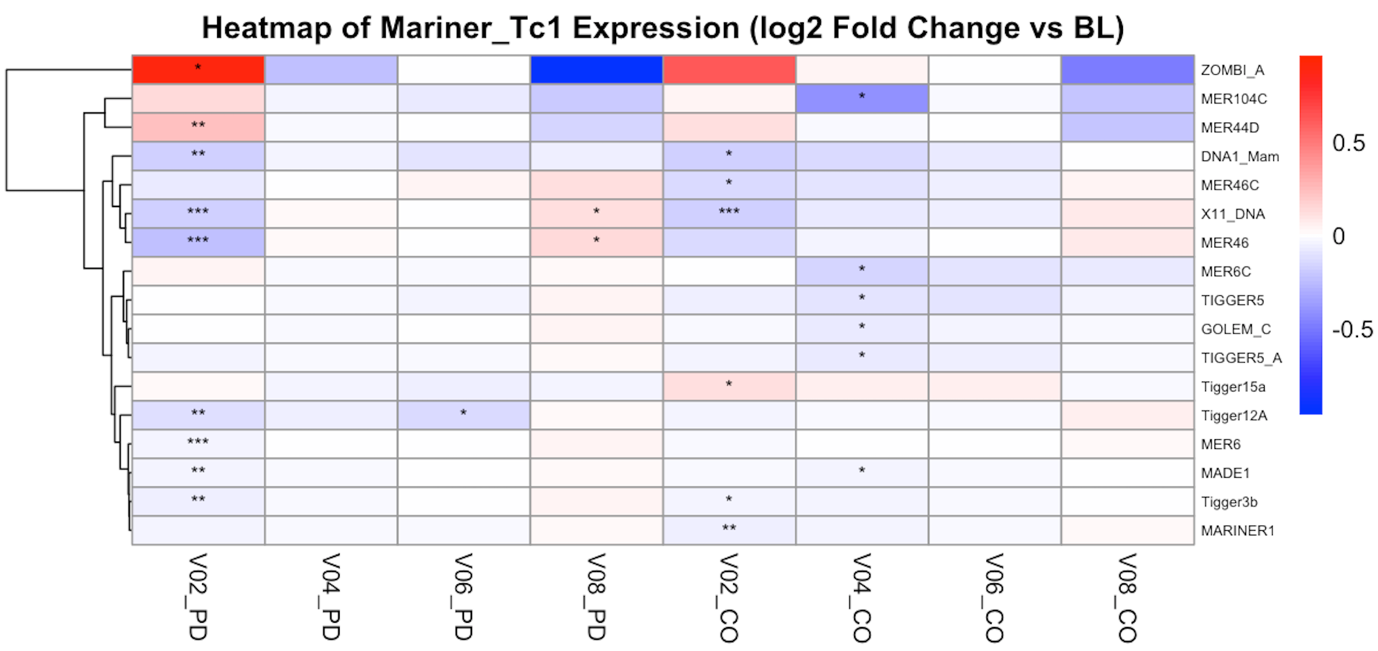


S4i, Other DNA elements


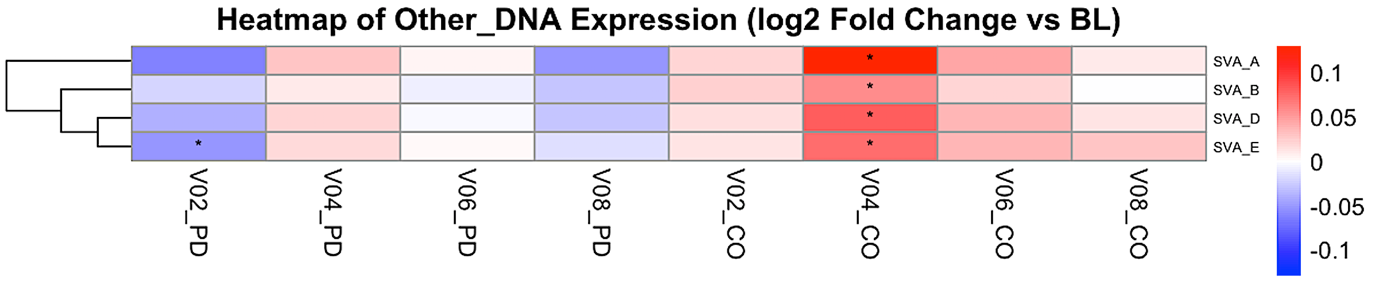


S4j, rRNAs


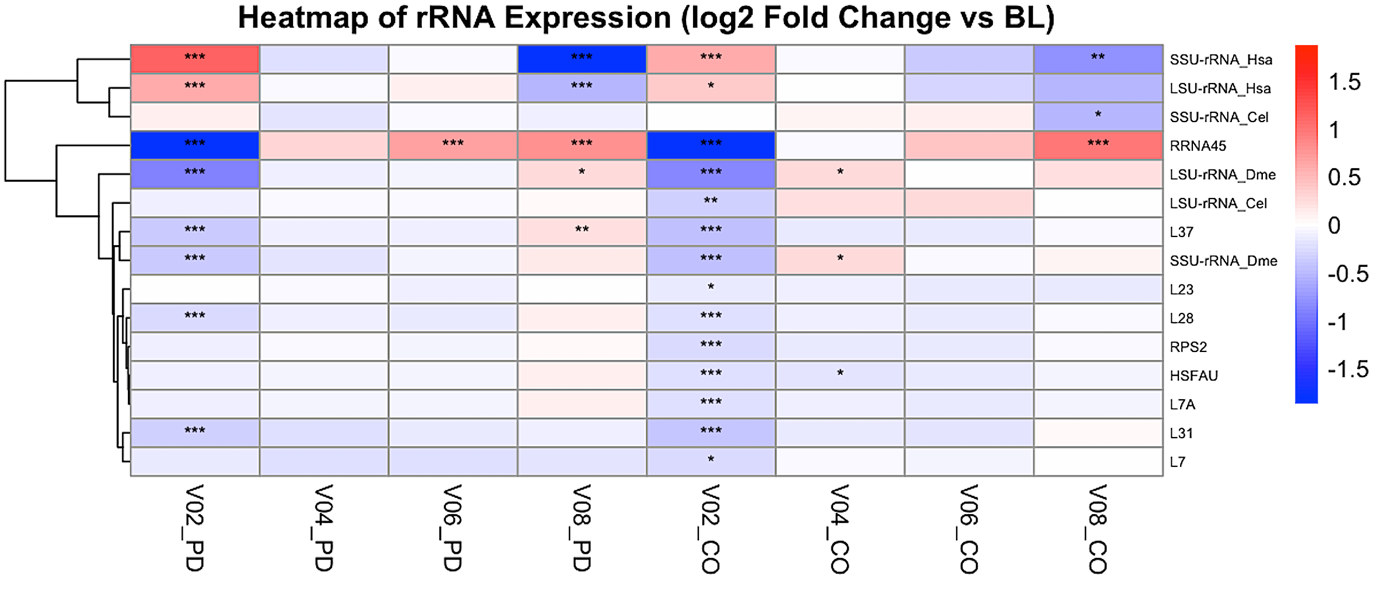


S4k, Satellites


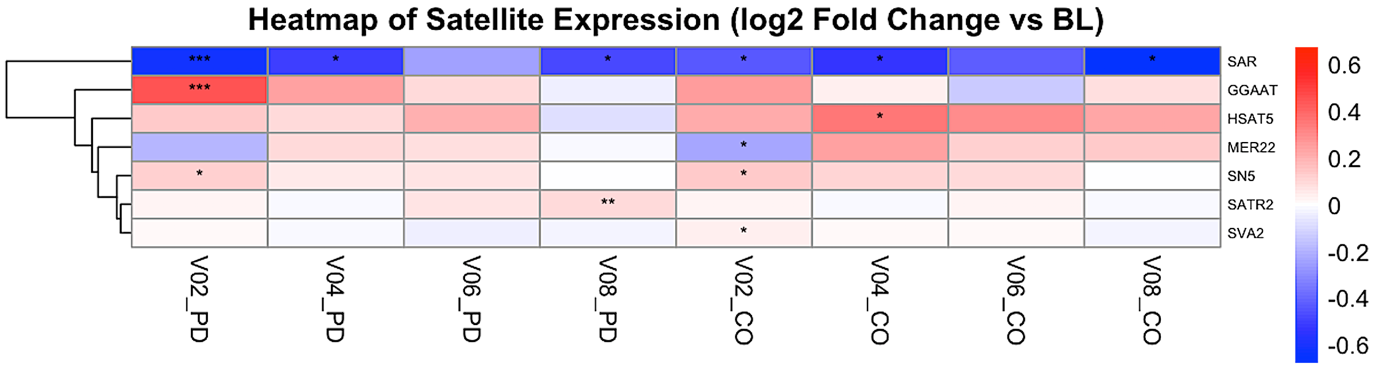


S4l, scRNAs


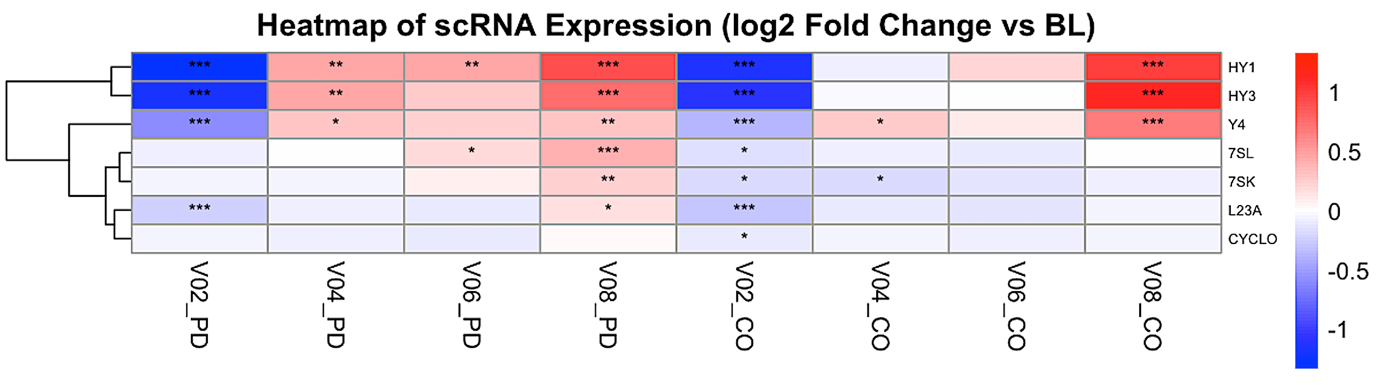


S4m, Simple Repeats


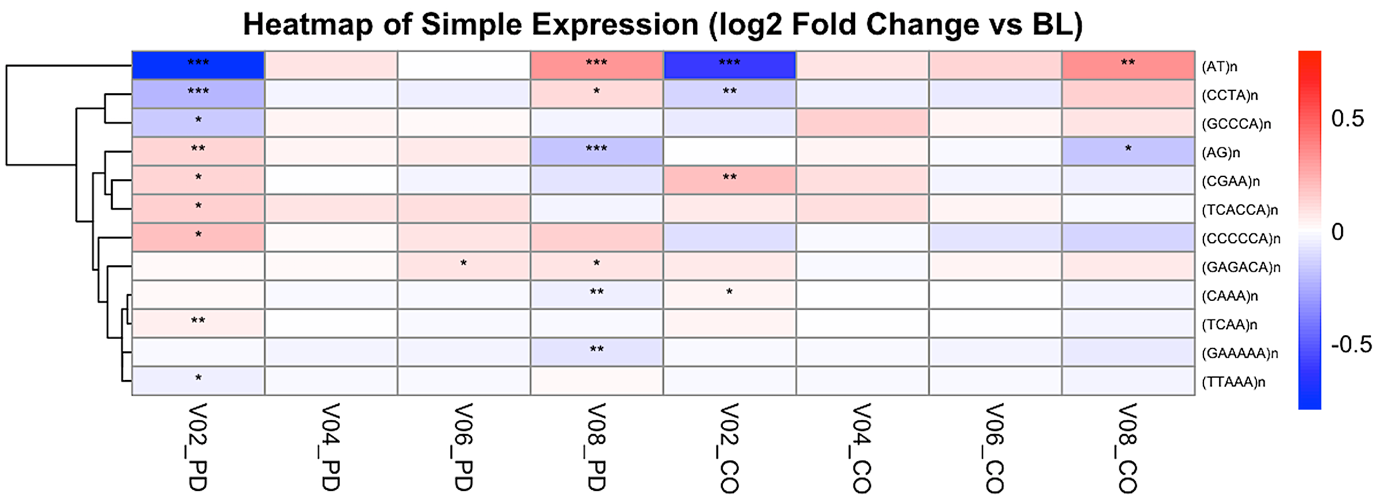


S4n, snRNAs


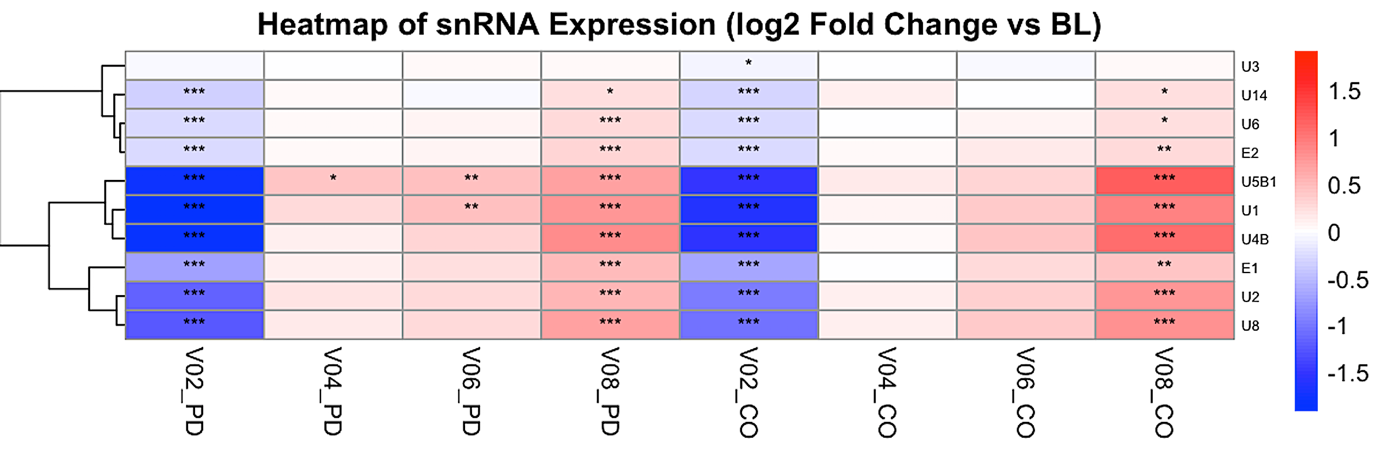


S4o, SVAs


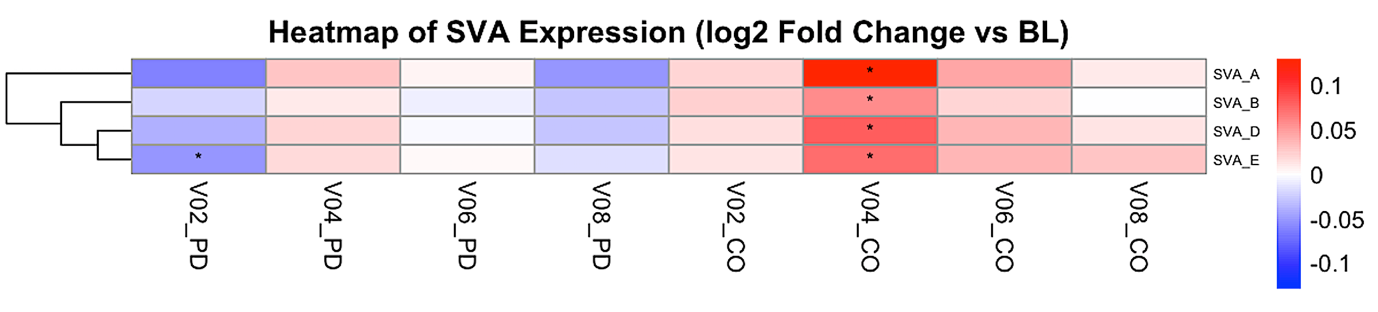


S4p, tRNAs


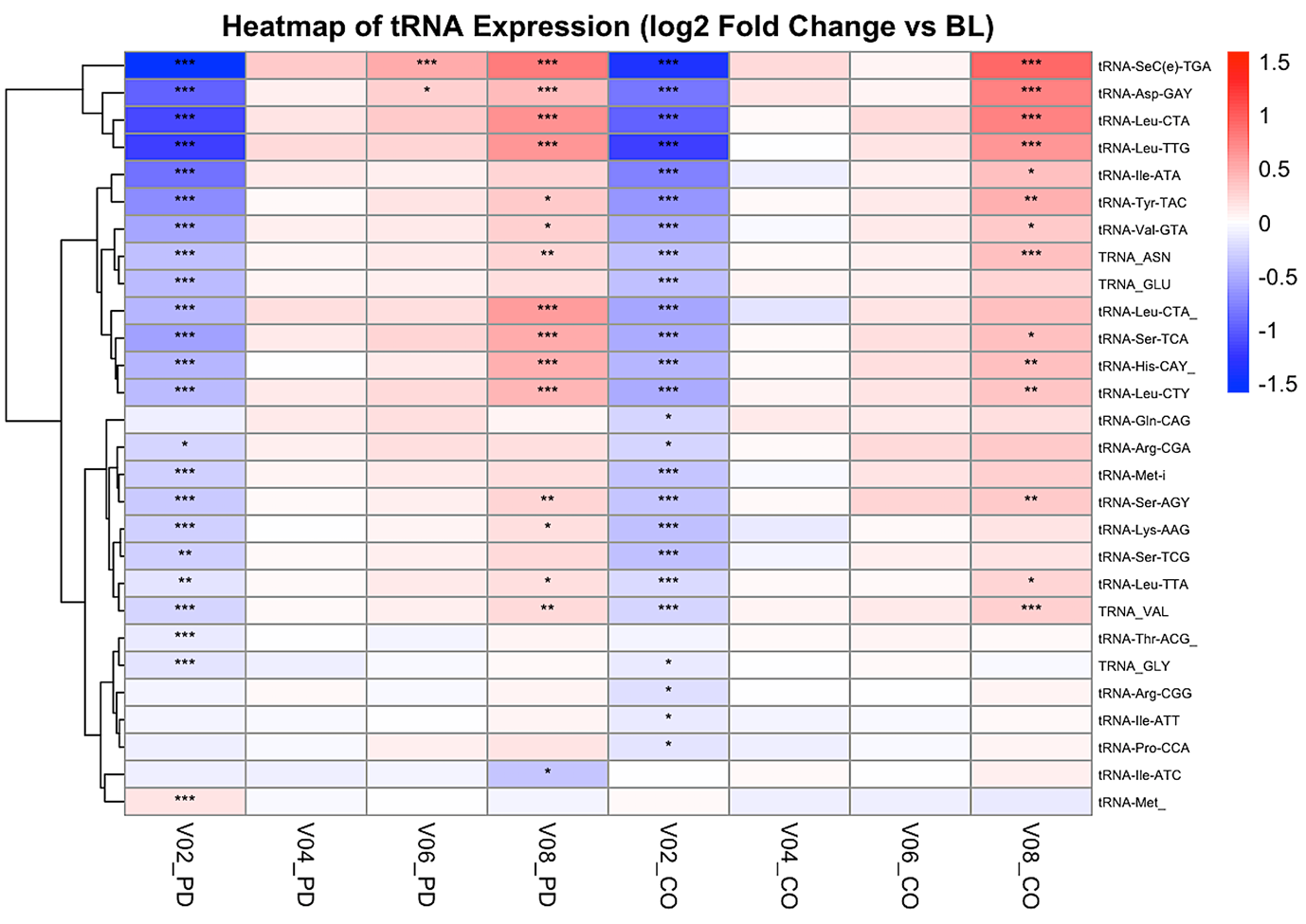


**Supplementary Figure 4a-p**. Heatmaps for differential expression patterns of 15 different Repeat subfamilies in peripheral blood cells of PD v BL and CO v BL groups at visits V02 (6 months), V04 (12 months), V06 (24 months), and V08 (36 months): a, Alus; b, CR1s, c, ERV1s, d, ERV2s; e, ERV3s; f, hATs; g, L1s; h, Mariner_Tc1s; I, Other-DNA elements; j, rRNAs, k, Satellites; l, scRNAs; m, Simple repeats; n, snRNAs; o, SVAs; and p, tRNAs. Heatmaps show log₂ fold change in expression for each element at each timepoint with a significant change in expression at least at one visit. Asterisks indicate statistical significance: padj < 0.05 (*), padj < 0.01 (**), padj < 0.001 (***). Heatmaps were created using the pheatmap R package. See Supplementary Materials3.RScripts
